# How do forelimb long bones adapt in rhinoceroses? An in‐depth examination of their microanatomy

**DOI:** 10.1111/joa.70180

**Published:** 2026-06-01

**Authors:** Cyril Etienne, Axel de Jesus, Vincent Fernandez, Alexandra Houssaye

**Affiliations:** ^1^ UMR 7179, Mécanismes Adaptatifs et Evolution, Muséum National d'Histoire Naturelle, Centre National de la Recherche Scientifique Paris France; ^2^ Imaging and Analyses Centre Natural History Museum London UK; ^3^ European Synchrotron Radiation Facility Grenoble France

**Keywords:** bone adaptation, compact bone, limb bones, trabecular bone, weight‐bearing

## Abstract

Rhinoceroses, as the second heaviest extant land mammals, show a skeleton highly modified for weight bearing. However, the limbs of these animals, which are still capable of galloping, do not have the columnar organisation of the more massive graviportal taxa, such as elephants. In this context, studying how the limb bones manage to meet their functional requirements can provide a better understanding of how bones adapt to biomechanical constraints. This study provides a detailed investigation of the inner structure of the three forelimb long bones of the five modern rhino species. For that, it resorts to the combination of two approaches for in‐depth descriptions and comparisons of the microanatomical organisation along the entire volume of the bones: 2D longitudinal and transverse sections and a recent 3D approach with a new methodology for visualising and quantifying trabecular bone density and anisotropy in addition to compact bone distribution throughout the bone. By comparing the microanatomical features with the forces exerted on the limb long bones of a rhinoceros at rest obtained through musculoskeletal modelling, this study seeks to highlight the extent to which the microanatomy within each entire bone and among these three long bones with varying shapes and functions reflects the biomechanical stresses imposed on them. The sample is of unprecedented size for studying the microanatomy of such large animals. This study originally shows how microanatomy varies within the bones, between the different limb bones and between the species in regard to the forces exerted on these bones, thereby improving our understanding of the bone form–function relationships. This study highlights the strong correspondence between the forces acting on the bones and their microanatomical structure and thus highlights the great potential of detailed study of bone microanatomy to improve our understanding of musculoskeletal adaptation in extinct taxa and, consequently, our palaeoecological inferences.

## Graphical abstract

Here, we study the bone structure in the forelimb long bones of the five extant species of rhinoceroses. By combining traditional description of virtual sections made on the bones (A) with in‐depth cartographies of the variation of bone compactness and anisotropy (B), we show how bone tissue can adapt to extreme forces and constraints in the second heaviest land mammals on Earth, such as in the ulnar olecranon shown here, subject to intense cranioproximal forces by the triceps brachii.

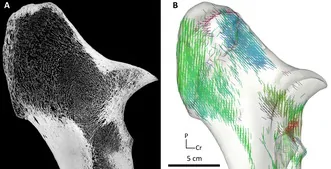

## 1 INTRODUCTION

The vertebrate skeleton is deeply linked to the lifestyle and morphology of the animal, and thus, its study is extremely informative to understand adaptations to specific functional constraints. Moreover, being often the only material (with teeth) preserved in the fossil record, bone is essential for reconstructing the lifestyle of past vertebrates (Cobb & Sellers, 2020; DeGusta & Vrba, 2003; Pintore et al., 2022). Bone shape and how it has evolved to accommodate the great variety of habitats, locomotion modes and sizes observed today in vertebrates are extensively studied with increasingly complex techniques (Belyaev et al., 2024; Meloro et al., 2013; Mitteroecker & Schaefer, 2022). In addition to bone shape, bone microanatomy, that is, the distribution of osseous tissues inside the bones, also bears a strong functional signal (Canoville et al., 2022; Carter & Beaupré, 2000; Currey, 2002; Houssaye & Botton‐Divet, 2018; Laurin et al., 2011). Cortical bone thickness varies depending on many parameters, such as body mass and lifestyle (Currey, 2002; Houssaye, 2009; Houssaye & Botton‐Divet, 2018; Wall, 1983). Trabecular bone varies notably in trabecular density and number and in trabecular orientation, which tends to be aligned with the principal stresses going through the bones, that is, the forces experienced (Currey, 2012; Dumont et al., 2013; Kivell, 2016). Bone microanatomy can thus be a powerful tool in functional morphology, bringing additional information compared with external morphology alone.

Very large land vertebrates (i.e. weighing several tons) are known to present specific adaptations, such as straighter limbs and more robust bones, to help reduce the stresses experienced by their skeleton (Alexander, 1985; Biewener, 2005; Hutchinson, 2021). They also present specific microanatomical adaptations, especially a thicker cortex and an extension of the trabecular bone inside the medullary cavity, that appear to vary depending on the clade studied (Bader et al., 2025; Currey & Alexander, 1985; Houssaye et al., 2016; Lefebvre et al., 2023; Wall, 1983). In rhinoceroses, the second heaviest extant land mammals on Earth and the heaviest capable of galloping (Alexander & Pond, 1992; Garland, 1983; Wilson & Mittermeier, 2011), bone shape and muscular adaptations have been extensively studied (Etienne et al., 2021; Mallet et al., 2019, 2020, 2022), but not the microanatomy. Only a handful of transverse sections and one longitudinal section have been published; they show a relatively thick cortex, with a trabecular bone filling the medullary cavity (de Buffrénil et al., 2010; Houssaye et al., 2016; Wall, 1983). This is overall consistent with what is observed in other heavy animals such as hippopotamuses, elephants and heavy dinosaurs (Bader et al., 2025; Houssaye et al., 2021; Lefebvre et al., 2023; Nganvongpanit et al., 2017), but differences can be expected. Rhinoceroses are close relatives of horses and tapirs and, like them, can adopt a galloping gait during which all four limbs are off the ground at a given point of the locomotion cycle (Alexander & Pond, 1992). They retain flexed legs, and thus, their muscles work at a greater mechanical disadvantage than elephants with their straighter (columnar) limbs (Biewener & Patek, 2018). This is bound to require specific adaptations on their bones, notably in their microanatomy. Their analysis will enable us to understand the trade‐off between locomotor and weight‐support requirements.

Before the advent of computed tomography (CT), notably based on X‐ray, studying microanatomy required destructive techniques that made it impossible to see the whole microstructure in 3D and prohibited its study on rare and large animals. CT scanning of complete bones of large animals is still complicated (for weight and vertical movement limitations inside the scanner, in addition to expensive scanning time), so that the resulting CT data are often partial (e.g. only the diaphysis for large hippos; Houssaye et al., 2016) or of too low resolution to distinguish individual trabeculae (Nganvongpanit et al., 2017), for such long bones. Thus, how microanatomy varies inside one bone alone (e.g. variations in cortical thickness along the diaphysis, changes in trabecular anisotropy and density in association with muscle insertion areas and contact areas) or between different bones with varying functions is largely undocumented.

This is regrettable, as X‐ray CT also allows for much greater quantification of anatomically and mechanically relevant metrics in 3D. Trabecular density can be directly measured as bone volume fraction (BVF; the proportion of total volume actually occupied by bone tissue) and anisotropy as a degree of anisotropy representing the main vector of orientation of the trabeculae (Deckers et al., 2022; Kivell, 2016; Maquer et al., 2015). Such metrics can be calculated directly on the entire bone or trabecular space, which would be, however, rarely meaningful given the high degree of variation usually observed in one bone (Houssaye et al., 2021; Lukova et al., 2024; Turcotte et al., 2026). However, they can also be calculated inside small regions of interest (ROI), presumably homogeneous in terms of trabecular architecture. They can be handpicked by the user, in order to make them as homologous as possible between different bones. For example, trabecular architecture of the femoral head is widely studied (Amson & Kilbourne, 2019; Georgiou et al., 2019; Greenwood et al., 2018; Mielke et al., 2018; Ryan & Shaw, 2013; Whitmarsh et al., 2019). Another alternative is to distribute many ROIs, often evenly spaced, inside bone tissue. Several studies produced detailed cartographies of BVF in hominoid bones, showing that BVF is higher underneath contact areas between the different skeletal elements than in the diaphysis and using these results to infer on the behaviour of fossil hominoids (Deckers et al., 2022; Dunmore et al., 2020; Gross et al., 2014; Skinner et al., 2015; Tsegai et al., 2017). Bishop et al. (2018), in a very detailed study, were able to characterise the anisotropy in fossil and extant theropod dinosaurs and to make inferences on the locomotor behaviour of the extinct taxa. Such computations, however, can be challenging because they require more computing power the more ROIs are studied. Moreover, although a greater number of ROIs typically gives a greater precision in the description of the microanatomical variations, having too many ROIs in one bone can make it hard to visualise these variations in practice. Recently though, these kind of computations have been facilitated by the Dragonfly software (Object Research Systems). Its Bone Analysis plugin can compute BVF and anisotropy inside evenly spaced spherical ROIs all across a bone automatically, resulting in potentially several million ROIs giving a clear, three‐dimensional mapping of the direction of the trabeculae (Lennie et al., 2021; Piché et al., 2018; Reznikov et al., 2022).

Such a precise analysis of the microanatomy of rhinoceros limb bones could be extremely interesting to better refine our understanding of bone functional anatomy. This would be highly appropriate now, as the direction and magnitude of the forces exerted on the limb long bones of a rhinoceros at rest have recently been studied in 3D through musculoskeletal modelling (Etienne et al., 2024), and could thus be compared to the microanatomy. The forelimb has a greater role in body support than the hindlimb, which has a greater role in body propulsion in ungulates, including horses and very likely, rhinos (Dutto et al., 2006; Witte et al., 2004). Therefore, this study investigates the three forelimb long bones to analyse the relationship between their microanatomy and the forces associated with high weight bearing in these cursorial animals. These constraints have been shown to impact the morphology of these bones (Mallet et al., 2019, 2020) and the associated musculature (Etienne et al., 2021) and should affect bone microanatomy as well. Moreover, rhinos comprise five different species, with varying mass, limb proportions and habitats. The lightest species, the Sumatran rhinoceros *Dicerorhinus sumatrensis*, weighs 600–950 kg and lives in highly forested habitats, whereas the heaviest species, the white rhinoceros *Ceratotherium simum*, weighs 1350–3500 kg and favours open plains (Dinerstein, 2011). This variation could also be reflected in the forelimb bone microanatomy. The microanatomy of a *Ceratotherium* humerus and of a radius and tibia of *Ceratotherium* and *Dicerorhinus* have indeed shown features associated with compressive strength, with differences between these two species (Etienne et al., 2025; Houssaye et al., 2024).

We anticipate seeing highly dense bones for rhinoceroses overall, with a medullar area filled by spongious bone and a thicker cortex, according to the previous microanatomical studies conducted on heavy taxa mentioned above. Moreover, we expect to observe higher bone density and anisotropy in areas subject to the highest forces. More generally, we expect trabecular microarchitecture to reflect the functional role of each bone, and thus to be more varied in the humerus since it is subject to a greater number of muscular insertion areas (Etienne et al., 2021). The microanatomy of more massive rhinos should also reflect the higher forces exerted on their bones.

Our study is innovative in proposing to combine intraindividual (between the three bones), intraspecific and interspecific scales of investigation, to characterise the form–function relationships in the microanatomy of these limb long bones. Here, using X‐ray micro‐CT, we have imaged entirely all the three forelimb long bones from 23 specimens of rhinos, belonging to all five species, at a resolution sufficient to clearly distinguish individual trabeculae. This results in 69 bones, an unprecedented sample size for the microanatomy of such large animals. We combine 2D longitudinal and transverse sections to describe the microanatomical organisation along the whole bones in the entire data set. We then use a recent method (Lennie et al., 2021) with a new approach to create 3D cartographies of the variations of bone volume fraction and anisotropy of trabecular bone inside the bones for one specimen of each species. We expect those cartographies to help us better characterise the 3D organisation of the trabecular bone and refine the descriptions made from the sections. We notably expect to be able to identify clear sets of anisotropic trabeculae associated with the spreading of the most intense forces exerted on the bones. This approach could also potentially reveal the patterns of trabecular architecture that were not visible on the 2D sections. We compare 2D sections and the cartographies with the available data on the relative forces acting on each of these bones, in conjunction with muscle attachments and contact areas, in a modelled rhinoceros standing at rest (from Etienne et al., 2024). This provides a unique opportunity to document the relationship between trabecular structure and the constraints acting on a bone throughout its entire volume. The combination of these two approaches should enable us to see if, and above all how, the microanatomy of these bones is adapted to the forces to which they are exposed.

If the link between forces and the microanatomy of these bones emerges clearly, this would increase our understanding of bone form–function relationships and of its adaptation but also highlight the great potential of using bone microanatomy to infer forces and muscle insertion areas in the bones of extinct taxa, which can be of great use to palaeontology (Aguirre et al., 2020; Bishop et al., 2018; Macchiarelli et al., 1999).

## 2 MATERIALS AND METHODS

### 2.1 Material

Our sample consists of the three forelimb long bones (humerus, radius and ulna) from the five living species of rhinos. Those are the white rhinoceros (*Ceratotherium simum*, body mass 1350–3500 kg), the black rhinoceros (*Diceros bicornis*, 800–1300 kg), the Indian rhinoceros (*Rhinoceros unicornis*, from 1350 to 2100 kg), the Javan rhinoceros (*Rhinoceros sondaicus*, 1200–1500 kg) and the Sumatran rhinoceros (*Dicerorhinus sumatrensis*, 600–950 kg; Dinerstein, 2011). A total of 69 bones belonging to 23 different specimens were studied here, with between three and six individuals per rhinoceros species (Table 1), for availability reasons. Adult specimens were systematically preferred over subadult ones (to avoid bias linked to ontogenetic variation), but were not always available, notably for *R. sondaicus* and *R. unicornis*. We would have preferred to use only wild specimens (to avoid biases related to differences in activity and food intake, in particular), but the choice was limited by the availability of the specimens. X‐ray micro‐CT was performed at two facilities: Specimens from the MNHN, RBINS and NMB were scanned at the MNHN's AST‐RX platform (UAR 2047; Baker Hughes Phoenix∣X‐ray v∣tome∣xs 240) and reconstructed with Phoenix datos|x. Specimens from the NHM were scanned at the NHM's micro‐CT laboratory (Nikon HMX 225 ST system) and reconstructed with the CT‐agent software (Nikon Metrology, Leuven, Belgium) (see Supplementary Data S1). Voxel size varies between 67 and 156 μm, but is usually close to 100 μm.

**TABLE 1:** Specimens studied and their provenance.

Species	Institution	Col. number	Sex	OS	Origin
*Ceratotherium simum*	MNHN	ZM‐MO‐2005‐297	M	A	C
*Ceratotherium simum*	NHM	2018.143	U	A	U
*Ceratotherium simum*	RBINS	1904	M	S	W
*Ceratotherium simum*	RBINS	35208	U	A	U
*Ceratotherium simum*	NMB	8029^a^	M	A	W
*Diceros bicornis*	MNHN	ZM‐AC‐1936‐644	F	S	Ci
*Diceros bicornis*	MNHN	ZM‐AC‐1944‐278^a^	M	A	C
*Diceros bicornis*	RBINS	9714	F	A	W
*Diceros bicornis*	NMB	10594	U	A	Ci
*Rhinoceros unicornis*	MNHN	ZM‐AC‐1960‐59^a^	M	A	C
*Rhinoceros unicornis*	NHM	ZD_1972.822	U	S	U
*Rhinoceros unicornis*	NHM	ZE_1950.10.18.5	M	S	W
*Rhinoceros unicornis*	NHM	ZE_1961.5.10.1	M	S	W
*Rhinoceros unicornis*	NMB	7351	M	S	W
*Rhinoceros unicornis*	NMB	n.N.009	M	S	U
*Rhinoceros sondaicus*	NHM	ZD_1871.12.29.7	M	S	W
*Rhinoceros sondaicus*	RBINS	1205F^a^	U	S	W
*Rhinoceros sondaicus*	NMB	10885	U	S	W
*Dicerorhinus sumatrensis*	MNHN	ZM‐AC‐1903‐300	M	A	W
*Dicerorhinus sumatrensis*	NHM	ZE_1948.12.20.1	U	A	U
*Dicerorhinus sumatrensis*	NHM	ZE_1949.1.11.1	U	S	W
*Dicerorhinus sumatrensis*	RBINS	1204^a^	M	A	W
*Dicerorhinus sumatrensis*	NMB	10529	F	A	C

Abbreviations: A, adult; C, captive; Ci, circus; F, female; M, male; MNHN, Muséum National d'Histoire Naturelle; NHM, Natural History Museum; NMB, Naturhistorisches Museum Basel; OS, ontogenetic stage; RBINS, Royal Belgian Institute of Natural Sciences; S, subadult; U, unknown; W, wild.

^a^ Specimen also studied with the 3D approach.

### 2.2 Methods

#### 2.2.1 2D sections

Virtual microanatomical sections were generated using the VGSTUDIO MAX software (v. 2.2, Volume Graphics GmBH). Each bone was oriented in an anatomical position following previous work (Barone, 1999; Mallet et al., 2019). At least three perpendicular section planes were defined: transverse, coronal and sagittal. The transverse section plane was defined as being perpendicular to the long axis of the bone and cutting through its centre of ossification, identified as the region where the nutrient canal reached the centre of the bone (Houssaye & Prévoteau, 2020; Nakajima et al., 2014). The coronal and sagittal sections were defined as cutting through the middle of the medullary space. For the ulna, the centre of ossification was located quite proximally, so that a second transverse section was added at mid‐diaphysis. Additionally, a second sagittal section was added, defined as cutting through the centre of the olecranon. Specimens were attributed an age class (adult or subadult; Table 1) based on the fusion of their epiphyses with the rest of the bone, with any kind of remnant of an epiphyseal line in the CT data being taken as indicating a subadult individual. All anatomical terms come from previous work by Mallet et al. (2019). All the sections were thoroughly compared on an intraindividual, intraspecific and interspecific basis.

#### 2.2.2 3D cartographies

The three forelimb long bones of only one specimen by species (15 bones) were used for 3D cartographies since the process is lengthy for such bones (see below), and since intraspecific variation appeared limited based on the virtual sections. The chosen specimens were *Ceratotherium simum* NMB 8029, *Diceros bicornis* MNHN ZM‐AC‐1944‐278, *Dicerorhinus sumatrensis* RBINS 1204, *Rhinoceros sondaicus* RBINS 1205F and *Rhinoceros unicornis* MNHN ZM‐AC‐1960‐59. Specimen selection was complicated, since many of them were drilled into to be displayed in museums, which makes a complete quantification impossible. Others had bone pathologies or were subadult with only partial epiphyseal fusion. Ultimately, all chosen specimens were adult males (except for the *R. sondaicus* specimen, of unknown sex) and wild bred (except for the *D. bicornis* individual, captive bred).

Segmentation was done with two approaches. The first method was manual segmentation with VGSTUDIO MAX software (Volume Graphics GmBH; for the humerus and radius of *R. unicornis* and the humerus of *D. bicornis*). The second method was semi‐automatic, using the Trainable Weka segmentation plugin of ImageJ (Arganda‐Carreras et al., 2017; Hall et al., 2009). This machine learning technique uses pre‐segmented image fragments to create a classifier based on a random forest algorithm that can be extrapolated for the segmentation of each section (Supplementary Data S2). Usually, one classifier was used per bone, but it was possible to use one classifier for two or three bones if they were scanned together. The raw and segmented stacks were then imported on VGSTUDIO MAX for verification and correction. Mainly, a ‘Refine region of interest’ operation was performed, in order to fit better the segmentation to the 3D shape of the trabeculae. This was necessary on these bones because the WEKA segmentation works in two dimensions, one section at a time. All three dimensions of the bone are thus not treated in the same way, which means that trabeculae oriented mainly along the transverse planes can be missed by the segmentation.

Following Lennie et al. (2021), the segmented bones were imported into the Dragonfly software (Object Research Systems 2021), in order to make cartographies of bone volume fraction (BVF) and anisotropy of all the bones with the BoneAnalysis module. The software creates small spherical regions of interest (ROI) whose centre is located along a three‐dimensional grid and calculates BVF and anisotropy inside those spheres. The following parts of the method describe the innovative approach for 3D visualisation of the whole bones we used here for the first time. For each species, the spacing of the ROIs and their radius was calculated based on the thickness and spacing of the trabeculae inside the humerus. To do this, trabecular and cortical bone in the humerus were manually separated using the Avizo software following the process used by Houssaye et al. (2018). Average trabecular thickness and spacing were then calculated across the entire trabecular space using the BoneAnalysis plugin of Dragonfly. Then, spacing of the ROIs was determined as the sum of trabecular thickness and trabecular spacing, and the radius of the spheres was twice their spacing (resulting in a significant overlap between the spheres: see Supplementary Data S3). Initially, trabecular thickness and spacing were also calculated for the radius and ulna, but were close enough between the bones that we chose to use the values for the humerus for the entire individual (e.g. spacing of 1.219, 1.268 and 1.478 mm, respectively, for the humerus, radius and ulna of *C. simum*). Even between the different species, the values of spacing are remarkably close (Table S2.1).

All the calculations were run on a Dell Precision workstation with 192 gigabytes of RAM, two Intel Xeon E5‐2643 v4 processors (6 cores/12 threads each, 3.40 GHz max refresh rate) and an Nvidia Quadro M5000 graphics card. Calculation of both BVF and anisotropy could be lengthy (up to 20 h of computation time for BVF alone depending on bone size), and some artefacts were associated with the initial calculation. Indeed, Dragonfly computes BVF on a single material, usually the segmentation of bone tissue. It considers BVF as the ratio of the number of voxels inside the material over the total number of voxels in the spherical ROI. This disregards whether or not all voxels in the ROI are actually part of bone space; indeed, for ROIs located at the edge of the bone, a significant proportion of the voxels is outside bone space. This artificially reduces the BVF of ROIs close to the edge of the bone (Supplementary Data S3). This was corrected by dividing the BVF of each ROI on the edge of the bone by the proportion of that ROI's voxels that are actually located within bone space, computed by using the BVF algorithm on a material of the whole bone (bone tissue plus bone marrow). ROIs whose centres were outside of the whole bone space were set to 0.

Dragonfly proposes two methods for anisotropy computation: a standard Mean Intercept Length algorithm, as found in other software (e.g. the BoneJ plugin of ImageJ), and a custom algorithm called Surface normals. That algorithm creates a 3D mesh from the segmented bone tissue voxels and smooths it to avoid sharp angles between former voxels. It then uses the normal vectors of the faces of the mesh to determine the preferred orientation of the trabeculae inside the ROI. The output result is a degree of anisotropy, ranging between 0 and 1, with 0 indicating an isotropic structure and 1 an anisotropic structure, with the direction of the anisotropy as a vector. This algorithm yielded results that were very coherent with the anisotropy observed in the 2D sections, contrary to the MIL algorithm that resulted in a lot of noise between ROIs, possibly because of the low number of voxels per trabecula (the Surface normals algorithms probably avoided that problem thanks to its smoothing step). The Surface normals algorithm is, however, extremely RAM intensive, due to having to generate a 3D mesh for a very high number of trabeculae.

Fortunately, Dragonfly only makes its calculations inside computation boxes, which usually encompass the entire bone but can be divided to lower necessary RAM. Some bones were separated in up to 11 different parts that were computed one by one. This resulted in up to 11 anisotropy cartographies, which were stitched together. That division did not impact the anisotropy values, as when calculating anisotropy in two overlapping computation boxes, anisotropy values were identical in the overlapping part. Dragonfly's anisotropy cartographies only give an information about the degree of anisotropy in each ROI, not the direction of that anisotropy, which is stored in a vector table (or several tables if the calculation was done in separate parts) that also contains the location of the centre of the ROI. The vector tables cannot be stitched directly in Dragonfly, so they were exported in CSV format and stitched using a custom Python script written by C.E. (Supplementary Data S3). The BVF and anisotropy cartographies were exported as a stack of .tiff images. Another Python script (also written by C.E.; Supplementary Data S4) combines the cartographies and the vector file and creates a final microanatomy CSV table. It consisted in a list of all the ROIs of the bone with their BVF, anisotropy, direction of the anisotropy vector, angle of the anisotropy vector with the principal axes of the bone (i.e. proximodistal, craniocaudal, mediolateral) and position. Depending on the bone, the files consisted of between 219,201 and 2,752,712 ROIs.

The microanatomy data set was imported into R, along with a 3D model of the entire bone exported from Dragonfly. At this point, the microanatomy data set can be filtered using any value, for instance keeping only ROIs below 0.85 BVF or above 0.5 anisotropy. Overall, we focused on trabecular architecture. To separate ROIs in trabecular bone from ROIs in cortical bone, we considered all ROIs with a BVF below 0.85 as trabecular, in accordance with Currey (2002) and as that value corresponded well to the separation as seen on the virtual stacks. On those trabecular ROIs, we computed the average of anisotropy and BVF, to compare bones and specimens. Histograms were extracted to visualise the distribution of BVF and anisotropy. Additionally, 2D histograms were made showing the correlation between BVF and anisotropy. In all those histograms, only the intensity of the anisotropy is considered, not its direction (e.g. proximodistally vs. craniocaudally oriented trabeculae). To visualise the ROIs, 3D cartographies were generated for each bone (Figure 1; see Supplementary Data S4 for the R code). The ROIs were displayed as vectors, pointing in the direction of the anisotropy. For the signal to be clearly visible, only the 25% most anisotropic regions were displayed. The number of ROIs was also downsampled by 2 in each dimension, again for better visibility. The 3D model of the bone was displayed above the ROIs, with transparency in order to see better the location of each ROI in the bone. The vectors could be coloured according to the direction of anisotropy, with green representing proximodistally oriented trabeculae, blue representing craniocaudal trabeculae and red representing mediolateral trabeculae, in order for their direction to be as clear as possible. When relevant, we instead colour‐coded them according to bone volume fraction in order to see variations of BVF in anisotropic regions, or according to the intensity of the anisotropy itself to better distinguish between high anisotropy and medium anisotropy regions.

**FIGURE 1:**
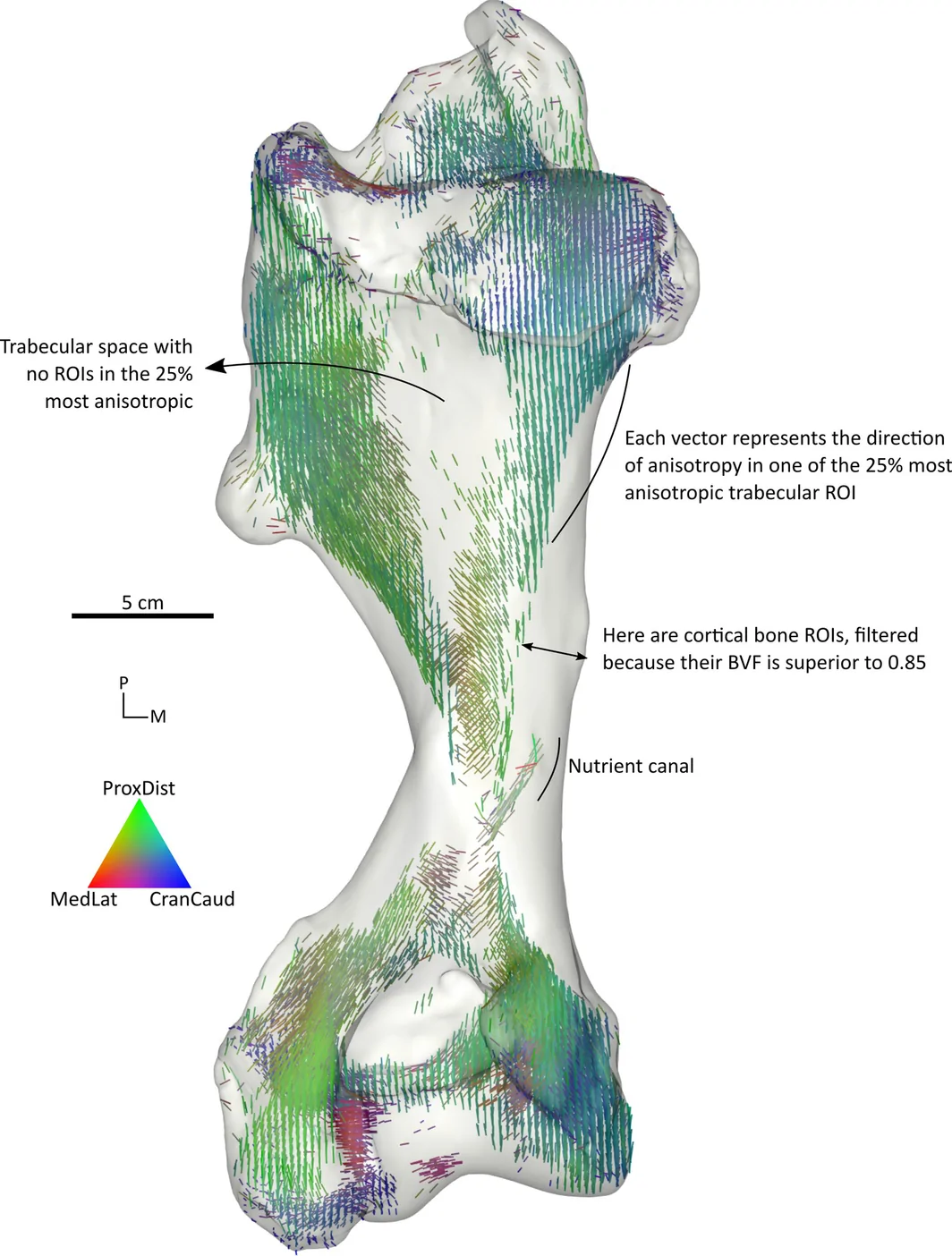
Example of a 3D cartography of trabecular anisotropy, with the main observable features described. Humerus of *Diceros bicornis* in caudal view. ROIs were first filtered according to their BVF, with ROIs with BVF >0.85 removed in order to keep only ROIs inside trabecular bone. Then, only the 25% most anisotropic ROIs were kept. They are displayed as vectors pointing in the direction of anisotropy. They are colour‐coded according to the direction of the vector. M, medial; P, proximal.

The patterns were compared to the forces exerted on the bones estimated based on the musculoskeletal model and simulation of *Ceratotherium simum* standing at rest in a previous study (Etienne et al., 2024; Figure 4; Tables 2, 3, 4). We present the general case in *C. simum*, the species for which a musculoskeletal model is available, and then compare it to the other rhinos.

## 3 RESULTS

In the section, we describe the microanatomical structure of each bone one by one. We begin by describing and comparing the virtual anatomical sections of all 23 specimens, in order to establish firmly what the general microanatomy of these bones looks like (with intraspecific variation presented in Supplementary Data S5). We then present in detail the results for the 3D cartographies in the *C. simum* individual analysed in order to complete the understanding and describe interspecific variation (with additional comparisons in Supplementary Data S6). We tentatively associate the microanatomical features observed with the forces exerted on the bones (Tables 2, 3, 4).

**TABLE 2:** Hypothesised association between forces exerted (based on Etienne et al., 2021, 2024), morphological features of the bones (as described in Mallet et al., 2019) and microanatomical features on the humerus.

Force(s) exerted	Morphological features	Trabecular features	Cortical features
Contact forces at the humeral head	Enlarged articular facet and humeral head spreading forces and on larger area	Anisotropic trabecular bone in the caudal aspect of the bone, linking articular facet to thick cortical bone (set a)	Thin cortex underneath the contact area, but sometimes thicker than the rest of the epiphysis Thick cortex in the diaphysis
Contact forces at the trochlea	Enlarged articular facet and distal humerus spreading forces and on larger area	Anisotropic trabecular bone in the medial part of the trochlea, linking articular facet to thick cortical bone (set g)	Thin cortex underneath the contact area, but sometimes thicker than the rest of the epiphysis Thick cortex in the diaphysis
*Subscapularis*	Convexity of the lesser tubercle	None observed	None observed
*Supraspinatus* and *infraspinatus*	Greater tubercle, significantly enlarged compared with other ungulates	Anisotropic trabecular bone going from the supraspinatus insertion to thick cortical bone	None observed
*Latissimus dorsi*	Tuberosity of the teres major	None observed	Slight thickening of the cortex due to the tuberosity.
*Deltoideus*	Deltoid tuberosity	Some, very superficial, mediolaterally anisotropic trabecular bone in the distal part of the tuberosity	None observed
Superficial pectorals	Humeral crest	Some proximomedially to caudolaterally orientated trabecular bone (set f), along what appears to be the entire insertion	Cortical bone is very thick, due to the insertion area being very close to the growth centre
Flexors of the carpus and digits	Medial epicondyle	Some anisotropic trabecular bone, proximodistally oriented (set i)	None observed
Extensors of the carpus and digits	Lateral epicondyle	Anisotropic trabecular bone from the tip of the epicondyle to the central cortex (h)	None observed
*Ulnaris lateralis*	Distalmost part of the lateral epicondyle, enlarged in rhinos (except *Dicerorhinus*)	Anisotropic trabecular bone from the tip of the epicondyle to the central cortex (h). Probably some craniocaudally oriented trabeculae due to compressive forces (k)	None observed
*Biceps brachii* reaction at the bicipital grove	Bicipital grove, relatively wider in heavier rhinos	Craniocaudally oriented trabeculae from underneath the grove almost to the humeral head	None observed

**TABLE 3:** Hypothesised association between forces exerted (based on Etienne et al., 2021, 2024), morphological features of the bones (as described in Mallet et al., 2019) and microanatomical features on the radius.

Force(s) exerted	Morphological features	Trabecular features	Cortical features
Contact forces at the articulation with the humerus	Enlarged articular facets, especially medially for larger rhinos	Anisotropic trabecular bone linking articular facet to thick cortex (sets a and b). More anisotropic on the medial side	Generally thin cortex underneath the contact area, but sometimes thicker medially Thick cortex in the diaphysis
Contact forces at the articulation with the carpus	Mediolaterally enlarged epiphysis	Anisotropic trabecular bone in the whole bone below the growth centre, much less present in the epiphysis (set c). Balanced between medial and lateral sides	Thin cortex underneath the contact area, but sometimes thicker than the rest of the epiphysis Thick cortex in the diaphysis
*Biceps brachii*	Radial tuberosity, significantly enlarged compared with other ungulates	Plate‐shaped trabeculae near the contact area might provide resistance to the cranioproximal pull of the *biceps* as well	Thicker than expected cortical bone for the proximal epiphysis

**TABLE 4:** Hypothesised association between forces exerted (based on Etienne et al., 2021, 2024), morphological features of the bones (as described in Mallet et al., 2019) and microanatomical features on the radius.

Force(s) exerted	Morphological features	Trabecular features	Cortical features
Contact forces at the articulation with the humerus	Mediolaterally enlarged articular surface with the humerus	Some anisotropic trabecular bone perpendicular to the contact, although very limited compared to what is observed between radius and humerus	Generally thicker cortex underneath the contact than in surrounding areas
Contact forces at the proximal articulation with the radius	Mediolaterally enlarged epiphysis	Some mediolaterally oriented trabeculae (set c)	Generally thicker cortex underneath the contact than in surrounding areas
Contact forces at the distal articulation with the radius	Bony tuberosity	Some anisotropic trabecular bone directed towards the articulation in the distal ulna (set f)	Some thickening of the cortex compared with surrounding areas
Contact forces at the articulation with the triquetrum	Some craniocaudal enlargement of the distal epiphysis	Some proximodistally oriented trabeculae in the epiphysis. Large set of anisotropic trabecular bone in the distal half of the cranial ulna (e)	Some thickening of the cortex compared with surrounding areas
*Triceps brachii*	Olecranon, longer and craniocaudally wider in rhinos than other ungulates, with also a mediolaterally enlarged olecranon tuberosity	Two large groups of anisotropic trabeculae, probably reacting to tension (a) and compression (b). Those are crossing in the middle of the extremity of the olecranon One large group of anisotropic trabeculae in the distal ulna (mediocaudal part, set d)	Some thickening of cortical bone, particularly the cranial aspect of the olecranon

For all rhinos, the three bones are strongly compact. Compact bone is usually thick in the diaphysis, especially around the growth centre. Conversely, it is thin in the epiphyses, particularly underneath the articular facets, usually barely thicker than an individual trabecula. There is no open medullary cavity, trabecular bone filling all the medullary area.

### 3.1 Humerus

#### 3.1.1 2D sections of the humerus

The growth centre is located approximately three quarters of the way down the diaphysis; its location is the same in all individuals. The nutrient canal may enter the bone through either the cranial or caudal side. Around the growth centre is the region where cortical bone is the thickest; it progressively becomes thinner moving away from the growth centre, until the metaphysis (Figure 2A–D). The medullary space thus has a double cone shape, similar to an hourglass, centred on the growth centre. Cortical bone is thicker in the medial side of the diaphysis than in the lateral side, and as thick on the caudal as on the cranial side (Figure 2A–D,K–O). Cortical bone in transverse section is the thickest in *D. bicornis*, then in *C. simum*, *R. sondaicus* and *R. unicornis* and the thinnest in *D. sumatrensis* (Figure 2K–O). Compact bone in the epiphyses is always much thinner than in the diaphysis and sometimes as thin as an individual trabecula (i.e. below 400 μm). Nonetheless, a few areas of the epiphyses may be marked by a thicker compact layer than surrounding areas: under the trochlear groove in sagittal view, and under the medial trochlea in coronal view (Figure 2B,D,H–J), although this is less visible in *D. sumatrensis*. Compact bone is thicker than the surrounding area as well under the crest of the lesser tubercle and under the bicipital groove (although several specimens of *D. sumatrensis* and *C. simum* are exceptions). Underneath the humeral head, some specimens present a thickening of the cortex (all *D. bicornis*, all *R. sondaicus*, some of each other species), and some do not (Figure 2B,D,E–G).

**FIGURE 2:**
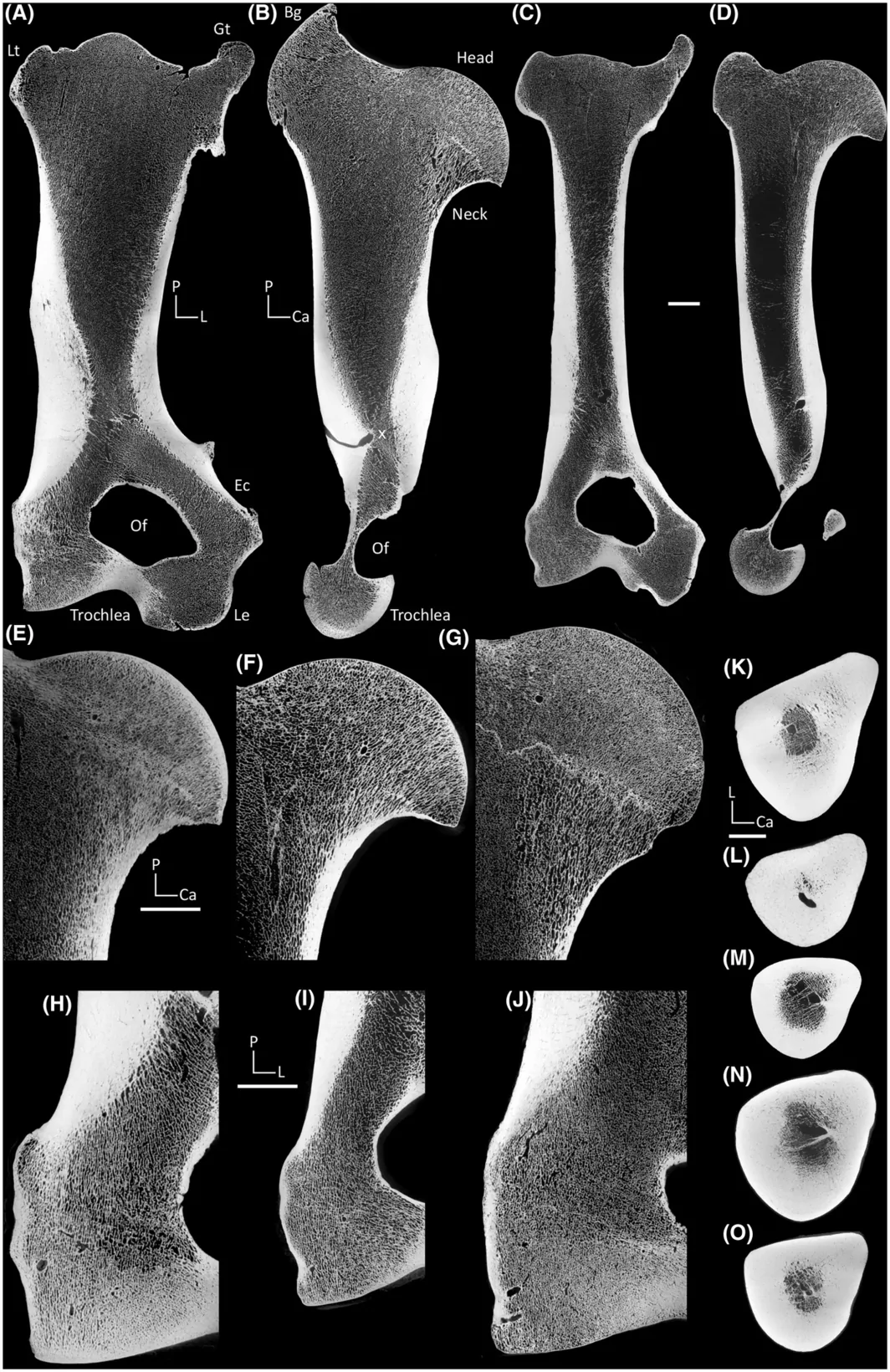
Virtual sections of the humeri. (A–D) coronal (A, C) and sagittal (B, D) sections of *C. simum* NMB 8029 (A, B) and *D. sumatrensis* MNHN ZM‐AC‐1903‐300 (C, D). (E–G) zoom on the humeral head, in sagittal sections, of *D. bicornis* RBINS 9714 (E), *D. sumatrensis* MNHN ZM‐AC‐1903‐300 (F) and *R. unicornis* NMB 7351 (G). (H–J) Zoom on the medial trochlea, in coronal sections, of *D. bicornis* NMB 10594 (H), *D. sumatrensis* MNHN ZM‐AC‐1903‐300 (I) and *R. unicornis* NMB 7351 (J). (K–O) transverse sections at the growth centre of *C. simum* NMB 8029 (K), *D. bicornis* MNHN ZM‐AC‐1944‐278 (L), *D. sumatrensis* MNHN ZM‐AC‐1903‐300 (M), *R. unicornis* NHM ZD 1871.12.29.7 (N) and *R. sondaicus* NHM ZE 1961.5.10.1 (O). Scale bars are two centimetres. Bg, bicipital groove; Ca, caudal; Ec, epicondylar crest; Gt, greater tubercle; L, lateral; Le, lateral epicondyle; Lt, lesser tubercle; Of, olecranon fossa; P, proximal. X (in B) indicates the approximate position of the growth centre.

Trabecular bone is moderately anisotropic in the greater tubercle, with trabeculae oriented mostly along a mediolateral axis, although variations between individuals occur. Under the bicipital groove trabeculae are oriented along an inclined craniocaudal axis in the proximocaudal part and along an inclined proximodistal axis in the distocranial part (Figure 2B). This is clearly visible in *C. simum* and *D. bicornis*, but less so in *Rhinoceros* and almost not at all in *D. sumatrensis*. Trabeculae are anisotropic and present very high trabecular density in the humeral head, particularly in the caudal part, orienting perpendicular to the border of the articular facet (Figure 2E–G). Trabeculae are even more anisotropic in the neck, aligned along the parasagittal plane and looking particularly plate‐like. In sagittal view, they curve progressively to align themselves with the proximodistal axis in the distalmost part of the neck, where the cortical bone begins to thicken (Figure 2E–G). The separation between the trabeculae of the neck and of the head is clearly visible in *Ceratotherium* and *Rhinoceros*, but much less visible in *Diceros* and not at all in *Dicerorhinus*, where the trabeculae of the head look very much like those of the neck (Figure 2). Trabecular density in the proximal epiphysis is the highest under the humeral head (especially the caudal half), then at the lesser tubercle and under the bicipital groove, and is lower elsewhere, although still higher than in the diaphysis. In the latter, the medullary space is entirely filled with spongiosa. In the part proximal to the growth centre, next to the cortex, trabecular density and anisotropy are high, especially on the caudal side of the bone. In the centre, both trabecular density and anisotropy gradually reach their lowest point in the entire bone; in two *D. sumatrensis* individuals, a remnant of empty medullary space is even present (Figure 2C,D). In the centre of the diaphysis, anisotropic trabeculae going from one side of the cortex to the other can be found, which in coronal view is much more visible in *D. sumatrensis* and *D. bicornis* (Figure 2D). In the part of the diaphysis distal to the growth centre, the trabeculae present a higher trabecular density than in the proximal part. In sagittal view, above the olecranon fossa, the trabeculae are highly anisotropic, running proximocranially from the border of the fossa. On the medial side of the metaphysis and epiphysis, trabeculae are highly anisotropic and plate‐shaped, aligned in the parasagittal plane and leading to the medial trochlea (Figure 2H–J). Under the medial trochlea itself, the trabeculae are slightly less anisotropic with a much higher trabecular density, an arrangement reminiscent of that observed under the humeral head and neck. Again, that arrangement is not visible in *D. sumatrensis*, but is visible in *D. bicornis* as in the other species. In the epicondylar crest, the trabeculae are anisotropic, oriented at an angle between the proximodistal axis and the principal axis of the crest (Figure 2A,C). The trabeculae in the lateral trochlea present no particular anisotropy, with a much lower trabecular density than those in the medial trochlea.

#### 3.1.2 3D cartographies of the humerus

The humerus of *C. simum* that we studied in 3D enables us to observe in the entire bone the distribution of anisotropy and BVF (Figure 3; Figure 7.1 in Supplementary Data S7). The latter are consistent with the descriptions from 2D sections in that low anisotropy regions are characterised by low BVF as well. This supports, at the scale of the entire bone, the hypothesis that these regions are subject to relatively weak forces. Conversely, regions with a higher BVF are usually characterised by a higher anisotropy, meaning that they are probably subject to higher forces (see Supplementary Data S8).

**FIGURE 3:**
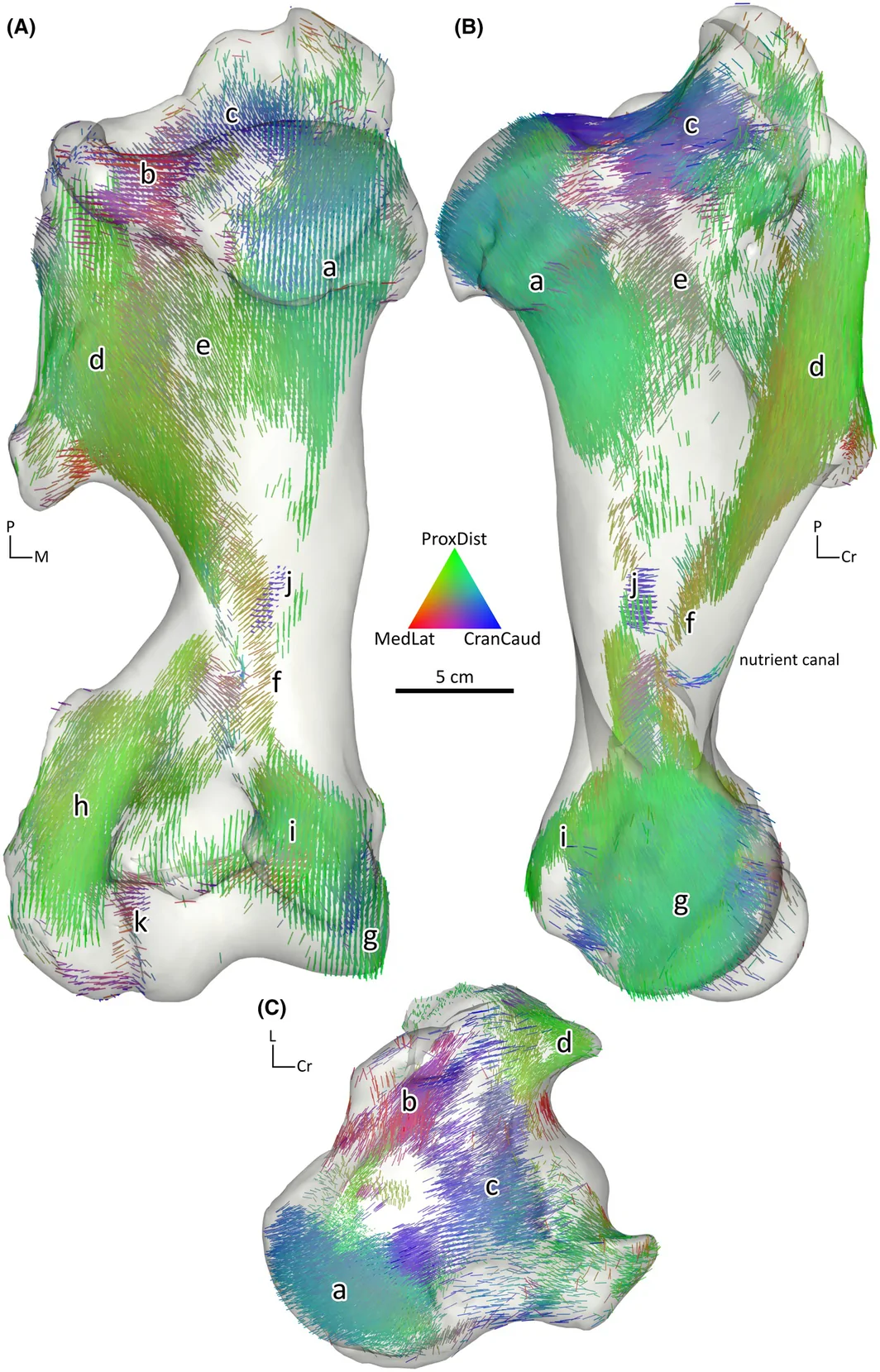
Microanatomy of the humerus in *C. simum*. (A) 3D cartographies of the 25% most anisotropic trabecular ROIs, in caudal (A), medial (B), and proximal (C) views. ROIs are represented as vectors pointing in the direction of the anisotropy and are additionally colour‐coded according to their direction. Lower case letters indicate sets of anisotropic trabeculae described in the main text. Cr, cranial; M, medial; P, proximal.

As was observed in the virtual sections, the humerus is more complex in terms of microstructure than the other bones. It presents a great diversity of anisotropic trabeculae. Most of them seem associated either with muscular or joint reaction forces (see Etienne et al., 2021, 2024 for muscle insertion areas and force magnitude and direction) (Figure 4; Table 2). Under the humeral head is a large set of anisotropic trabeculae (noted ‘a’ in Figure 3), that first begin near the contact area (with the scapula) at a 45° angle with the proximodistal axis and progressively become more vertical in more distal regions. Those trabeculae arise from the contact area and carry the forces to the thick cortex. A similar set is observed underneath the medial humeral trochlea (noted ‘g’). Those are particularly medially placed, confirming observations from the sections. Trabecular bone shows a markedly higher BVF in the epiphysis than in the metaphysis (Figure 5E). Several muscular insertions generate sets of anisotropic trabeculae, presumably aligned with the tensions exerted by the muscles. Most prominent are the *supraspinatus* and *infraspinatus* insertions in the craniolateral part of the proximal half of the bone (noted ‘d’), and the *ulnaris lateralis* in the lateral epicondyle (noted ‘h’). Those muscles are among those that deliver the greatest forces and insert on the epiphyses, where no thick compact bone is present to spread the forces over a large surface. A great amount of anisotropic trabecular bone was thus expected. Set ‘d’ of anisotropic trabeculae could also help resist forces from the *omotransversarius* and *brachiocephalicus* that are likely highly active for limb protraction during locomotion (Etienne et al., 2021). Trabeculae from set ‘h’ could also help resist to the pull from the other muscles inserting on the lateral epicondyle, namely the digital and carpal extensors, but those have highly different functions compared to the *ulnaris lateralis*; notably, they have no antigravity action (Etienne et al., 2021, 2024). A similar arrangement, although less marked, is observed for the carpal and digital flexors in the medial epicondyle (‘i’). In the sections, we did not observe anisotropic trabeculae underneath the insertion for the superficial pectorals, but we do observe such anisotropic trabeculae here, all along the insertion of the superficial pectorals (noted ‘f’ in Figure 3). These trabeculae appear to be either outside or perpendicular to the section planes; that is why they were not observed on the virtual sections.

**FIGURE 4:**
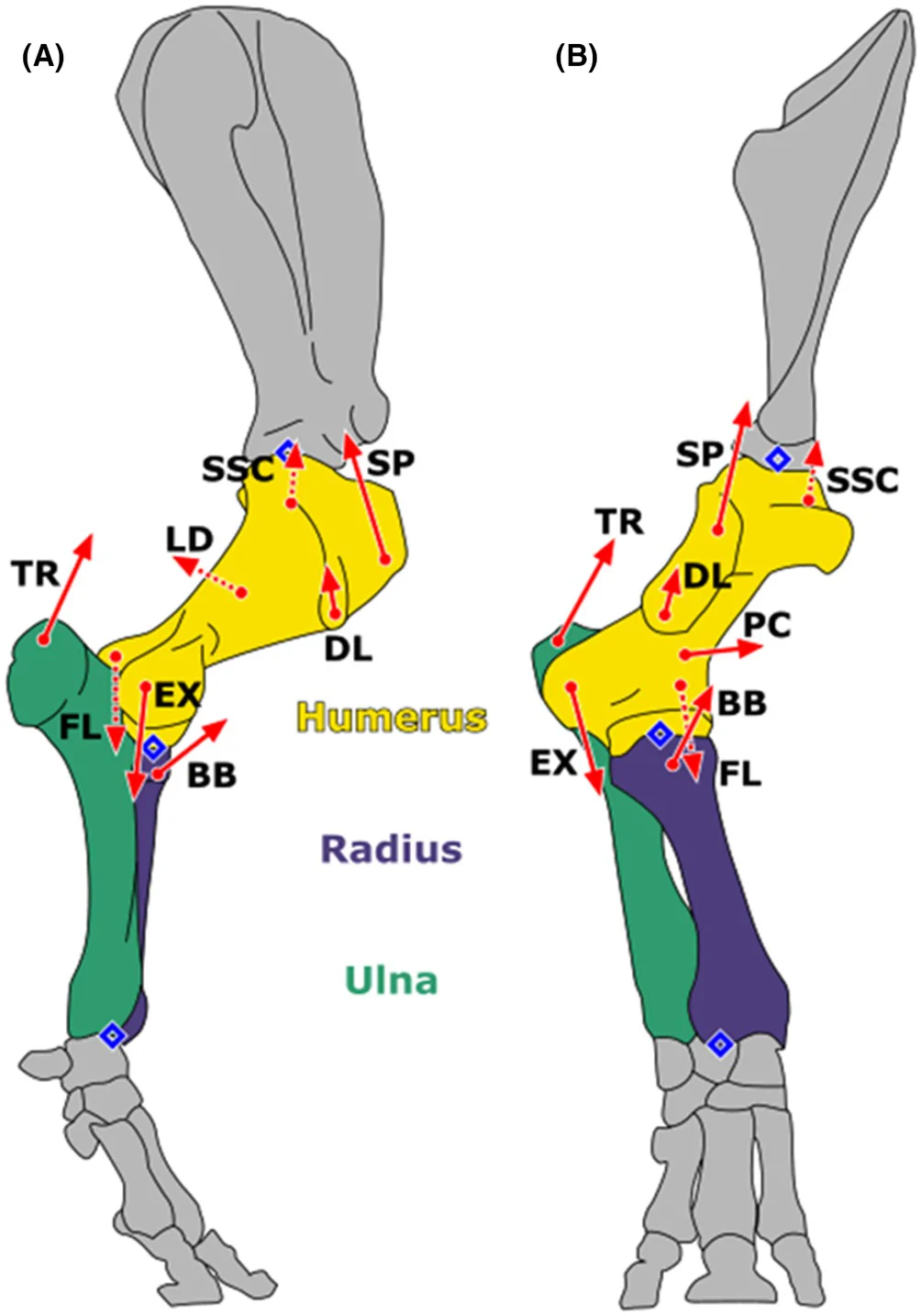
Muscular forces exerted on the limb long bones. Summary of the maximal forces that can be exerted by the principal muscle groups on the long bones of the forelimb of a rhinoceros. Lateral (A) and cranial (B) views. The skeleton is that of *C. simum*. Arrow length is proportional to the average of the relative maximal isometric force of the muscles in *C. simum* and *R. unicornis*, as calculated in Etienne et al. (2021). Force direction is based on Etienne et al. (2024). Dashed arrows represent muscles that insert on the opposite side of the bone. Forelimb: BB, *biceps brachii*; DL, *deltoideus*; EX, extensors of the digits and carpus, and *ulnaris lateralis*; FL, flexors of the digits and carpus; LD, *latissimus dorsi*; PC, superficial pectorals; SP, *supraspinatus* and *infraspinatus*; SSC, *subscapularis*; TR, *triceps brachii*. Blue diamonds indicate the approximate position of joint reaction forces at the articulation between each limb segment (approximately in the centre of the joints).

**FIGURE 5:**
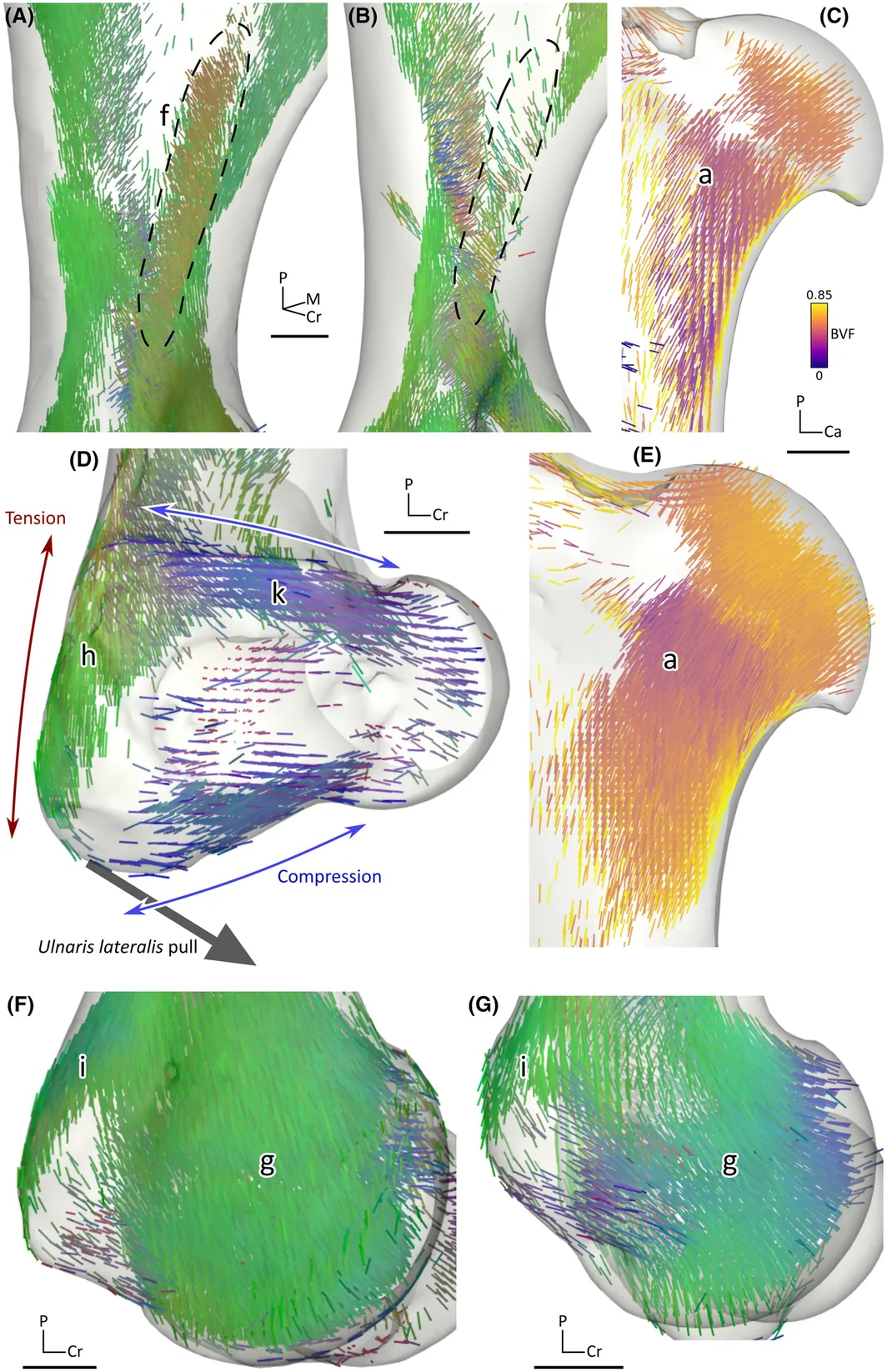
Comparisons of the microanatomy of the humerus between selected specimens. The 25% most anisotropic ROIs are displayed as vectors pointing in the direction of the anisotropy. (A, B) Centre of the humerus in *R. unicornis* (A) and *R. sondaicus* (B), craniolateral view. C, E. Humerus of *D. sumatrensis* (C) and *C. simum* (E), lateral view. Only the medial half of the bone is displayed, in order to clearly show the trabeculae in the core of the humeral head. (D) Lateral epicondyle of *D. bicornis* in medial view, with arrows representing muscle pull and the hypothesised resulting tensions and compressions in the bone. The medial half of the bone has been cut off in order to only show the medial epicondyle. (F, G). Medial part of the trochlea in *R. unicornis* (F) and *D. sumatrensis* (G), medial view. Specimens A, B, D, F and G have their ROIs colour coded according to direction as in Figure 3, specimens c and e have their ROIs colour coded according to BVF. Lower case letters indicate sets of anisotropic trabeculae described in the main text. Scale bar is 2 cm. Ca, caudal; Cr, cranial; M, medial; P, proximal.

It seems that some forces can be intense or frequent enough (probably both in the case of the superficial pectorals, given their role in limb stabilisation) to generate anisotropic trabeculae even below ~2 cm of cortical bone. Muscles, by pulling in one direction, can generate a tension on one side and a compression on another side (or several). We can identify such patterns here, which could not be identified with 2D sections alone. The vast network of trabeculae from the *supraspinatus* and *infraspinatus* insertion (‘d’ in Figure 3), loaded in tension, is complemented by two other sets (‘b’ and ‘e’ in Figure 3) loaded in compression, which comprise less anisotropic trabeculae. Set ‘b’ could also serve to resist forces from the *infraspinatus*, which inserts right next to it. Set ‘e’ is very close to set ‘a’, located underneath the contact area, loaded in compression as well and eventually seems to merge with it inside the thick cortex in the centre of the bone. Set ‘e’ could also provide some resistance against the pull of the lateral head of the *triceps brachii*, as it inserts on the tricipital line immediately proximolateral to the beginning of set ‘e’.

The anisotropic trabeculae from the superficial pectorals insertion (‘f’) seem to present a complementary set of trabeculae loaded in compression (‘j’), although it appears much less extended proximodistally than set ‘f’. The trabeculae originating beneath the *ulnaris lateralis* insertion (‘h’) seem also to present a small set of anisotropic trabeculae (‘k’) resisting the compression that is probably generated at the mediocranial border of the lateral epicondyle. Not all trabeculae whose arrangement seems associated with particular muscles are situated underneath an insertion area. The bicipital groove presents a set of anisotropic trabeculae (‘c’), which probably counteracts the pressure exerted by the *biceps* tendon on the bicipital groove when the muscle contracts (Etienne et al., 2021). Some regions are notable by not presenting anisotropic trabeculae where they would have been expected based on the musculoskeletal model, for example, the insertion areas of the deep pectorals (*subclavius* and *pectoralis ascendens*), and the *teres major* tuberosity (which is also the insertion of the *latissimus dorsi*).

Overall, of all those sets of anisotropic trabeculae, the two sets underneath the contact areas (‘a’ and ‘g’ in Figure 3) and the two sets underneath, respectively, the insertion of the *supraspinatus* and *infraspinatus* (‘d’) and *ulnaris lateralis* (‘h’) are by far the most extensive in size. They also present a higher anisotropy than the others. It is striking how similar they are: they all run from the area where the forces are applied in the epiphysis to the thick cortex, where the forces can be spread on a great bone surface.

Overall, few differences are observed between the different rhinos. *D. bicornis* is generally very similar to *C. simum*. It does, however, present a different orientation of the trabeculae in the humeral head and medial trochlea, with trabeculae more inclined relative to the proximodistal axis. *D. bicornis* also presents relatively more trabeculae in some sets that seem associated with a compression caused by muscle pull, rather than a tension, such as sets ‘b’ and ‘k’. It also presents another set in the distal border of the lateral epicondyle, probably serving the same function as set ‘k’ (Figure 5D). It has almost no anisotropic trabeculae in its deltoid tuberosity, contrary to all the others which present at least some.

The humerus of *R. unicornis* is strikingly similar to that of *C. simum*. With a few exceptions, it presents the same sets of anisotropic trabeculae in the same locations. The set of trabeculae originating from the *ulnaris lateralis* (‘h’) seems smaller, expanding less cranially and laterally. It is very visible in this species that this set of trabeculae originates from the tip of the lateral epicondyle, truly at the insertion of the *ulnaris lateralis* and not at the insertion of the other muscles inserting around the lateral epicondyle (Etienne et al., 2021). In the very thin part of bone between the coronoid fossa and the olecranon fossa, *R. unicornis* presents many proximodistally anisotropic trabeculae that are not present in the other species, except in *C. simum*, although to a much lesser extent, but this is because other species (*R. sondaicus*, *D. bicornis*, *D. sumatrensis*) do not have trabecular bone there, only compact bone. Some anisotropic trabeculae may also be positioned differently because of bone shape differences. For instance, the greater tubercle is more cranially developed in *R. unicornis*; as a result, the trabeculae from set ‘b’ (compressive forces associated with the *infraspinatus* insertion) are more cranial as well and more craniocaudally orientated than in *C. simum* and *D. bicornis*. That pattern is shared by *R. sondaicus* and *D. sumatrensis*, which also present a cranial development of the greater tubercle (Mallet et al., 2019).

*R. sondaicus* is very close to other rhinos in general. A set of trabeculae next to the insertion of the infraspinatus is well visible in *R. sondaicus* and *D. sumatrensis*, and partially in *C. simum*, but not in the others. The main difference seems to be that *R. sondaicus* hardly presents any anisotropic trabeculae underneath the insertion of the superficial pectorals (set ‘f’; Figure 5A,B), whereas those are very well marked in *R. unicornis*.

*D. sumatrensis* is similar to the other rhinos. It presents some artefactual ROIs with a high anisotropy near its medullary cavity, since the borders of the cavity are obviously anisotropic. Underneath the contact area at the medial trochlea (set ‘g’), its trabeculae clearly change orientation depending on their position (Figure 5F,G); they are proximodistal below the distal part of the articulation and craniocaudal below the cranial part. This change is observed in the other species but to a much lesser extent. *D. sumatrensis* also presents more anisotropic trabeculae in the medial epicondyle (set ‘i’) than the others. A second set of anisotropic trabeculae is even visible (Figure 5G, distocaudal part of the bone), probably loaded in compression by the muscles inserting on the medial epicondyle. This species does not share the development of the lateral epicondyle of the others, which gives the *ulnaris lateralis* muscle a relatively large lever arm in elbow extension and makes it so useful for resisting gravity. Perhaps, this results in some of the functions of the ulnaris lateralis being carried by the muscles inserting on the medial epicondyle (functionally similar to the *ulnaris lateralis*), resulting in more anisotropic trabeculae there.

In all bones, the nutrient canal is clearly visible, cutting through cortical bone. This is artefactual: the nutrient canal contains no trabeculae and is technically part of cortical bone. However, it is not bone tissue, so its presence drops the BVF of ROIs near it below 0.85, and being a canal, it is highly anisotropic, which is why it appears here. It enters the humerus through the cranial aspect in *C. simum*, but through the caudal aspect in all the others.

### 3.2 Radius

#### 3.2.1 2D sections of the radius

The growth centre is located one‐third of the way down the diaphysis and does not vary in position. The nutrient canal enters the bone through the caudolateral aspect. As in the humerus, cortical bone is thicker around the growth centre and becomes progressively thinner when moving away from the growth centre (Figure 6A–D). However, in all species but *D. sumatrensis*, cortical bone is thicker than expected in the proximal third of the cranial part of the radius, under the radial tuberosity (Figure 6B,D). *D. bicornis* again has the thickest cortex in the whole diaphysis, with some specimens again presenting transverse sections with only cortical bone, followed by *C. simum*, *R. sondaicus* and *R. unicornis*. *D. sumatrensis* has once again the relatively thinnest cortical bone (Figure 6E–I). In coronal view, cortical bone is thicker on the medial part of the bones in *C. simum*, and to a lesser extent in *D. sumatrensis*, *R. sondaicus* and *D. bicornis*; medial and lateral parts are almost of equal thickness in *R. unicornis*. In sagittal view, in *C. simum* again, the cranial part of the cortex is markedly thicker than the caudal part; in all other species, this is the case with most individuals but far less marked, and some individuals present the same thickness in the cranial and caudal parts. In the proximal epiphysis, all species show a thickening of compact bone under the articular surface (Figure 6A,C,J). This is less visible in *R. unicornis*, but very well marked in *C. simum* and *D. bicornis*, for which that thickening is greater on the medial side of the contact area. In the distal epiphysis, a slighter thickening is also observed in most species, particularly around the articular facet for the scaphoid (Figure 6K,L). In *R. unicornis*, only the region around that facet is thickened.

**FIGURE 6:**
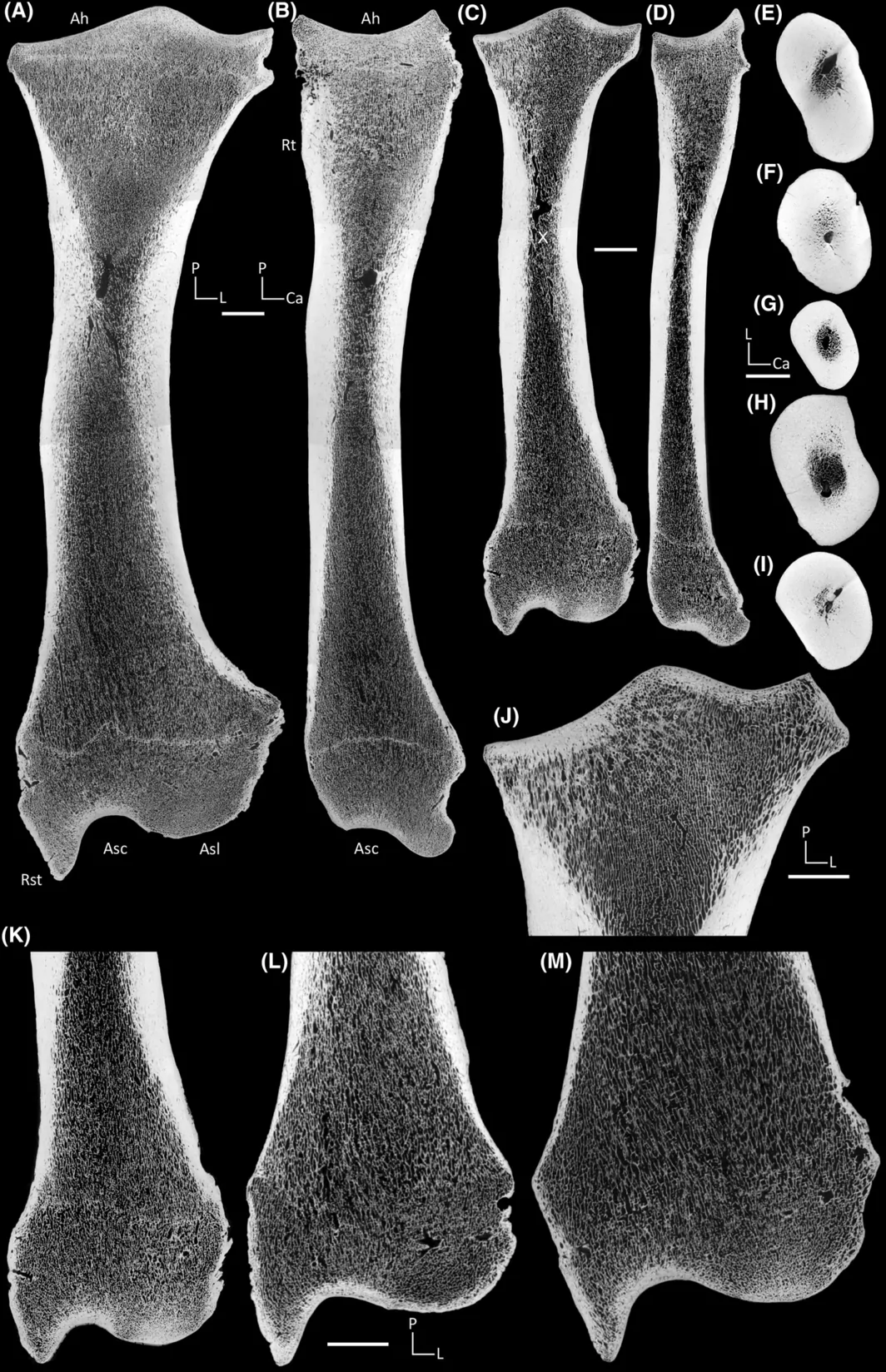
Virtual sections of the radii. (A–D) Coronal (A, C) and sagittal (B, D) sections of *R. unicornis* NMB n.N.009 (A, B) and *D. sumatrensis* ZM‐AC‐1903‐300 (C, D). (E–I) Transverse sections at the growth centre of *C. simum* NMB 35208 (E), *D. bicornis* NMB 10594 (F), *D. sumatrensis* MNHN ZM‐AC‐1903‐300 (G), *R. unicornis* NHM ZE 1950.10.18.5 (H) and *R. sondaicus* NHM ZE 1961.5.10.1 (I). (J) Zoom on the proximal epiphysis of C. simum NMB 8029, coronal section. (K–M): Zoom on the distal epiphysis, in coronal sections, of *D. sumatrensis* ZM‐AC‐1903‐300 (K), *D. bicornis* MNHN ZM‐AC‐1944‐278 (L) and *C. simum* NMB 8029 (M). Scale bars are two centimetres. Asc, articular surface for the scaphoid, Ah, articular surface for the humerus; Asl, articular surface for the semilunar; Ca, caudal; L, lateral; P, proximal; Rst, radial styloid process; Rt, radial tuberosity. X (in C) indicates the approximate position of the growth centre.

In all bones, the medullary space is entirely filled with spongiosa. Overall, trabecular anisotropy is higher and more homogeneous than in the humerus (Figure 6A–D,J–M). In coronal view, all over the bone, the trabeculae appear highly anisotropic and plate‐shaped, oriented nearly along the proximodistal axis (Figure 6J). The only region without those anisotropic trabeculae is the distal end of the distal epiphysis, where their organisation progressively becomes more random, and their trabecular density increases slightly. On the medial and central sides of the proximal part of the bone, the trabeculae are nearly perfectly aligned with the proximodistal axis; in the most lateral side, they are slightly inclined, going from proximolateral to distomedial (Figure 6J). Below the growth centre, trabecular orientation is at an approximately 10–20° angle to the proximodistal axis, this time from medial to lateral side. This angle is the highest in *C. simum* and the lowest in *D. sumatrensis* (Figure 6K–M). In sagittal view, trabeculae are again highly anisotropic all over the bone, and in contrast to the coronal view, they still appear anisotropic in the distal epiphysis, although less than in the rest of the bone.

In the proximal epiphysis, trabeculae radiate from the contact area with the humerus, joining the cortex. In the part of the bone distal to the growth centre, trabeculae mainly orient at a 15° angle from the proximodistal axis, going from the cranial to the caudal direction. At the caudal border however, in the distal third of the bone, trabeculae are more vertical and sometimes slightly inclined from the caudal to cranial direction. In the nearest part to the distal border, trabeculae are perpendicular to the contact zone. No major differences were observed between the species in terms of trabecular anisotropy. Trabecular density is overall the highest in the proximal epiphysis, especially on the medial part (Figure 6J). It is also high in the distal epiphysis, and it is the lowest in the middle of the diaphysis, especially in the part distal to the growth centre, where the medullary cavity would usually be located. *D. bicornis* presents significantly higher trabecular density than the other species.

#### 3.2.2 3D cartographies of the radius

The 3D cartographies confirm the much simpler general microanatomy of the radius observed in the virtual sections, in accordance with its more straightforward loading pattern than in the humerus. This is clearly visible on the 2D histogram of anisotropy and BVF, on which most regions comprise between 0.25–0.45 BVF and 0.4–0.7 anisotropy (Supplementary Data S8). Some ROIs, however, present a higher BVF, between 0.45 and 0.85 BVF, and are characterised by a high anisotropy, around 0.7. Those trabeculae are probably subject to the highest loads in the bone, hence the need for higher BVF. ROIs do not vary much in the orientation of their anisotropy throughout the bone, almost all of them being proximodistally aligned. Because of this, we coloured them according to BVF instead of anisotropy direction (Figure 7). Trabeculae present higher BVF in the proximomedial part of the bone, with a very dense set going straight from the medial part of the articular surface with the humerus to the thick cortical bone near the growth centre (set ‘a’ in Figure 7). However, contrary to the humerus, the radius also presents visible anisotropic ROIs underneath the lateral part of the articular surface (‘b’), although they are less anisotropic and present lower BVF than those in the medial part. Around the proximal contact with the ulna, some trabeculae seem to be directed towards the contact, probably helping resist contact forces between those bones (Table 3). The trabeculae in the centre of the bone are aligned with the vertical axis of the limb, but the trabeculae that are in a more caudal or cranial position are aligned with the long axis of the radius, and thus the forces at rest. Furthermore, regions are more anisotropic on the caudal border than in the centre of the bone and in the centre than in the cranial border.

**FIGURE 7:**
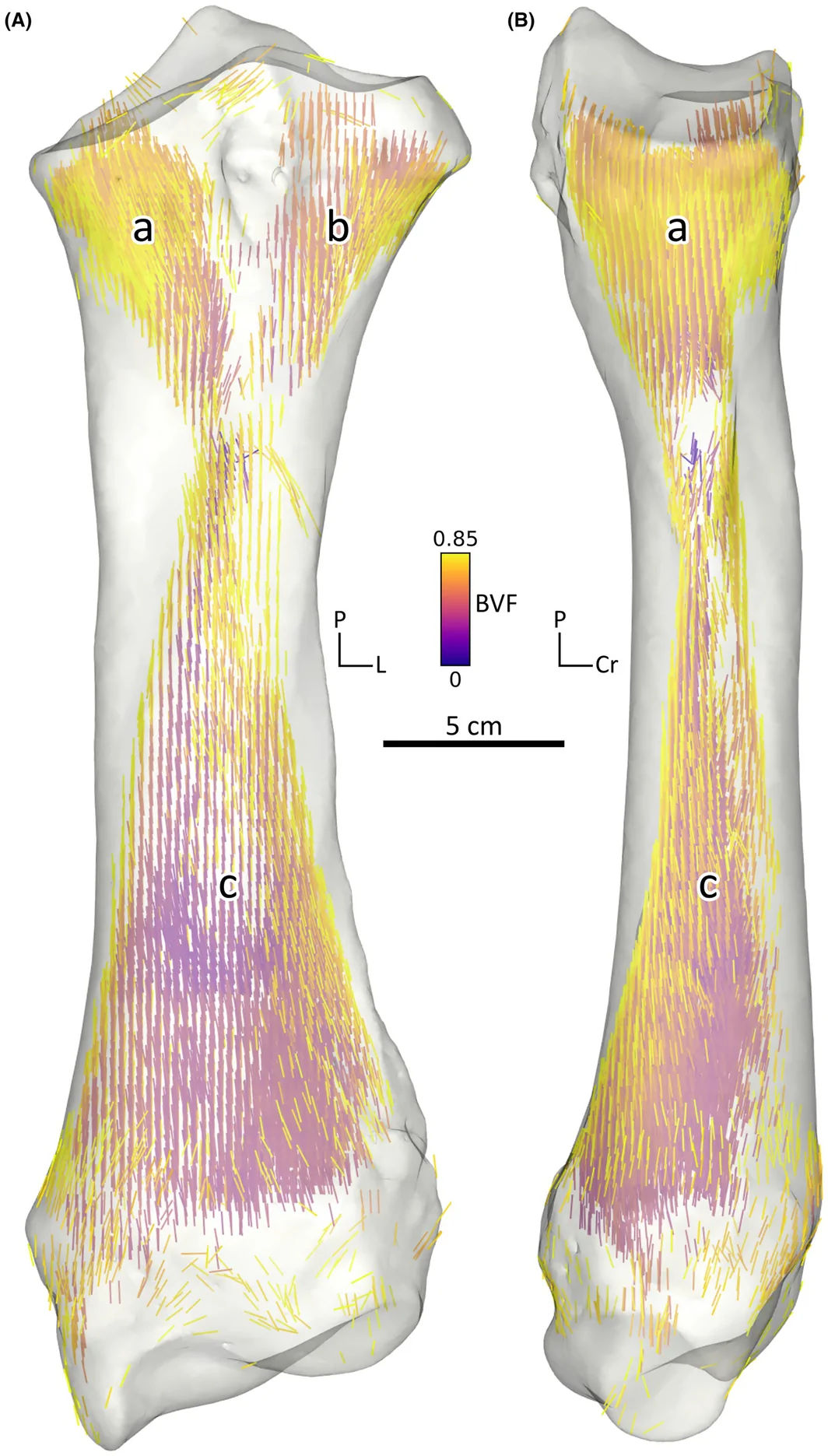
Microanatomy of the radius in *C. simum*. (A) 3D cartographies of the 25% most anisotropic trabecular ROIs, in cranial (A) and medial (B) views. ROIs are represented as vectors pointing in the direction of the anisotropy and are colour‐coded according to their BVF. Lower case letters indicate sets of anisotropic trabeculae described in the main text. Cr, cranial; L, lateral; P, proximal.

Overall, the distal two‐thirds of the bone are quite homogeneous (set ‘c’). Notably, as observed in the sections, the trabeculae are more homogeneous between the medial and lateral parts than within the proximal third. They also have a lower BVF, probably because this region of the bone is larger than the trabecular sets in the proximal third. As such, the forces will naturally spread on a greater surface of bone (both cortical and trabecular) without having to increase trabecular BVF. The biceps, despite its great force output exerted even at rest, is not associated with trabeculae directly in the direction of its pull. This is probably because trabeculae in this region are plate‐shaped, and even if they are primarily oriented proximodistally, they can still provide great resistance to a proximocranial tension. No other muscle is associated here with anisotropic trabeculae, which is consistent with the low maximal force output of these muscles (Etienne et al., 2021).

Very few variations are observed between the species. The main difference is that other species do not have as many anisotropic trabeculae underneath their lateral contact area with the humerus (Figure 8A,B). This mostly reflects that those trabeculae are less anisotropic, going below the 25% most anisotropic threshold we used, but the difference is in fact not as marked as is shown here with a hard 25% threshold. No trabeculae directed towards the proximal contact with the ulna were seen in other species than *C. simum*, even when lowering the 25% threshold. In the distal part of the bone, the arrangement where trabeculae in the centre of the bone are more inclined than trabeculae on the caudal part of the bone was well visible in all species. *R. unicornis* and *D. sumatrensis* stand out also by having more anisotropic trabeculae in the lateral part of the distal end of the radius, whereas lateral and medial parts are quite balanced in the other species (Figure 8D). This probably reflects higher forces coming from the lateral part of the wrist in these individuals/species. In *R. sondaicus*, the epiphyseal line is clearly visible in the distal part of the bone, producing a break in the anisotropy (Figure 8C); this could slightly lower the average anisotropy of *R. sondaicus*' bones. *R. unicornis* stands out by the lower BVF of its trabeculae, especially in the distal two‐thirds of the bone.

**FIGURE 8:**
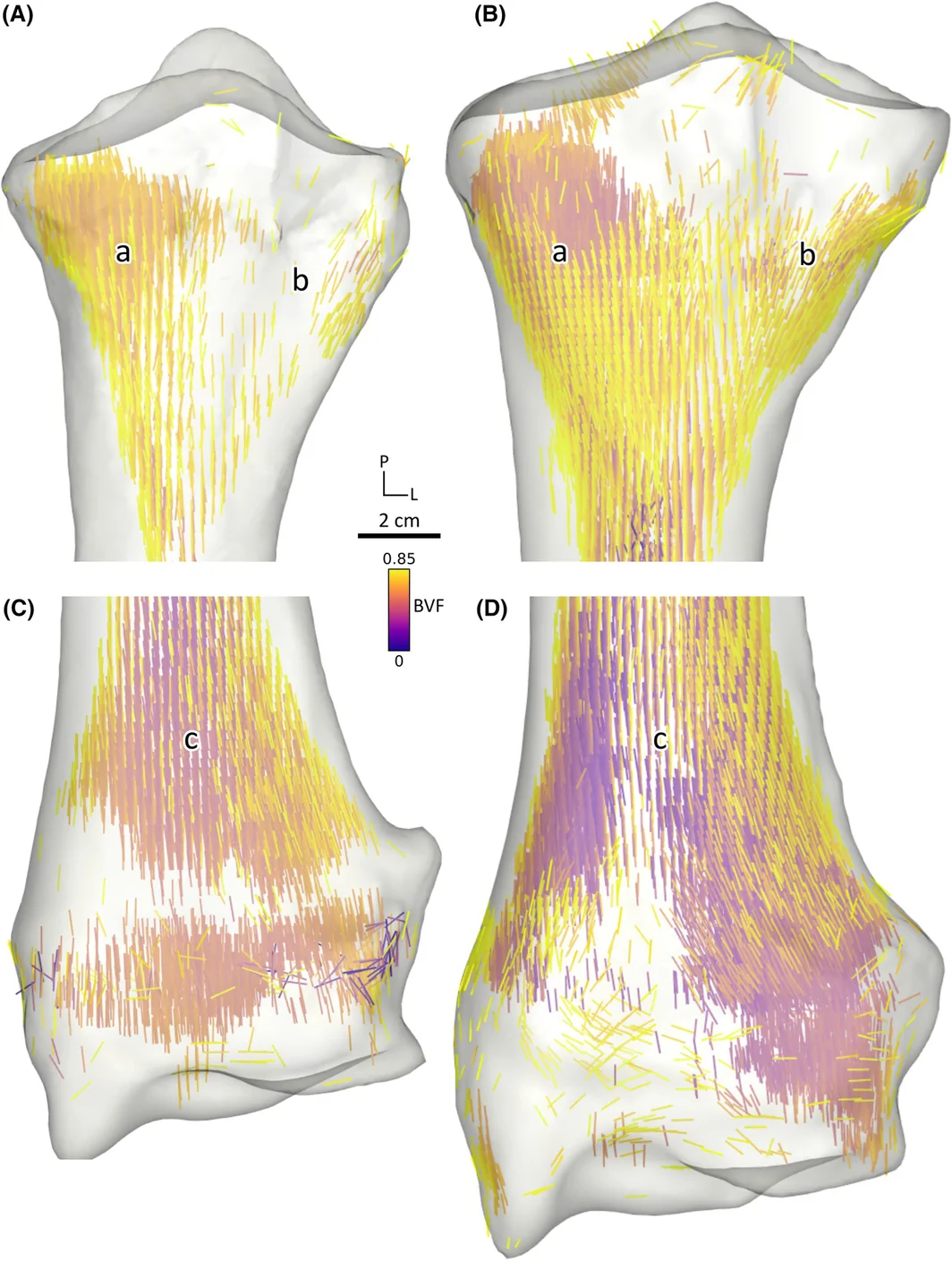
Comparisons of the microanatomy of the radius between selected specimens. The 25% most anisotropic ROIs are displayed as vectors pointing in the direction of the anisotropy, coloured according to BVF. (A, B) Proximal third of the radius in *D. bicornis* (A) and *R. unicornis* (B), cranial view. (C, D) Distal third of the radius in *R. sondaicus* (C) and *R. unicornis* (D), cranial view. Lower case letters indicate sets of anisotropic trabeculae described in the main text. L, lateral; P, proximal.

### 3.3 Ulna

#### 3.3.1 2D sections of the ulna

The growth centre is located between the diaphysis and the olecranon, caudal to the articular surface with the humerus. The nutrient canal enters the bone through the proximal part of the cranial aspect of the diaphysis and then travels proximally to join the growth centre. As for the humerus and radius, cortical bone is thicker at the growth centre, especially in the caudal part of the bone, at the caudal junction between the diaphysis and olecranon (Figure 9A–D). This part seems thicker in *C. simum* and *D. bicornis* than in the others, but this is difficult to judge because the position of the olecranon compared to the diaphysis varies, and even small shape variations may lead the cutting plane to cut into a part of the cortex that is not perpendicular to the sectional plane, making it appear thicker. On the transverse sections, at the growth centre, the cortex is thicker in *D. bicornis*, filling the entire medullary space, whereas in the other species some trabecular bone is always visible (Figure 9G–K). At mid‐diaphysis, the cortex is thicker in *D. bicornis* again, but this time it does not fill the entire section. The other four species are similar, although *D. sumatrensis* may have a relatively slightly thicker cortex than the others (Figure 9L–P). In the olecranon, in *D. sumatrensis*, the cortex seems thicker on the whole tuber olecrani. In *D. bicornis* and *C. simum*, only the proximal part of the tuber presents a clear, relatively thick cortex, with the caudal part showing a porous cortex that is hard to distinguish from dense trabeculae (Figure 9Q,R). In *Rhinoceros*, the whole olecranon presents such a porous cortex hard to distinguish from trabeculae. All species present a thickening of the cranial border of the olecranon, especially in the most distal part, but this is less marked in *R. unicornis*. Below the contact area with the humerus, cortical bone is usually thicker than in surrounding areas, but again this is less marked in *R. unicornis*. Cortical bone in the distal part of the bone remains quite thin, sometimes thickening a bit below the articular facet for the triquetrum (Figure 9F).

**FIGURE 9:**
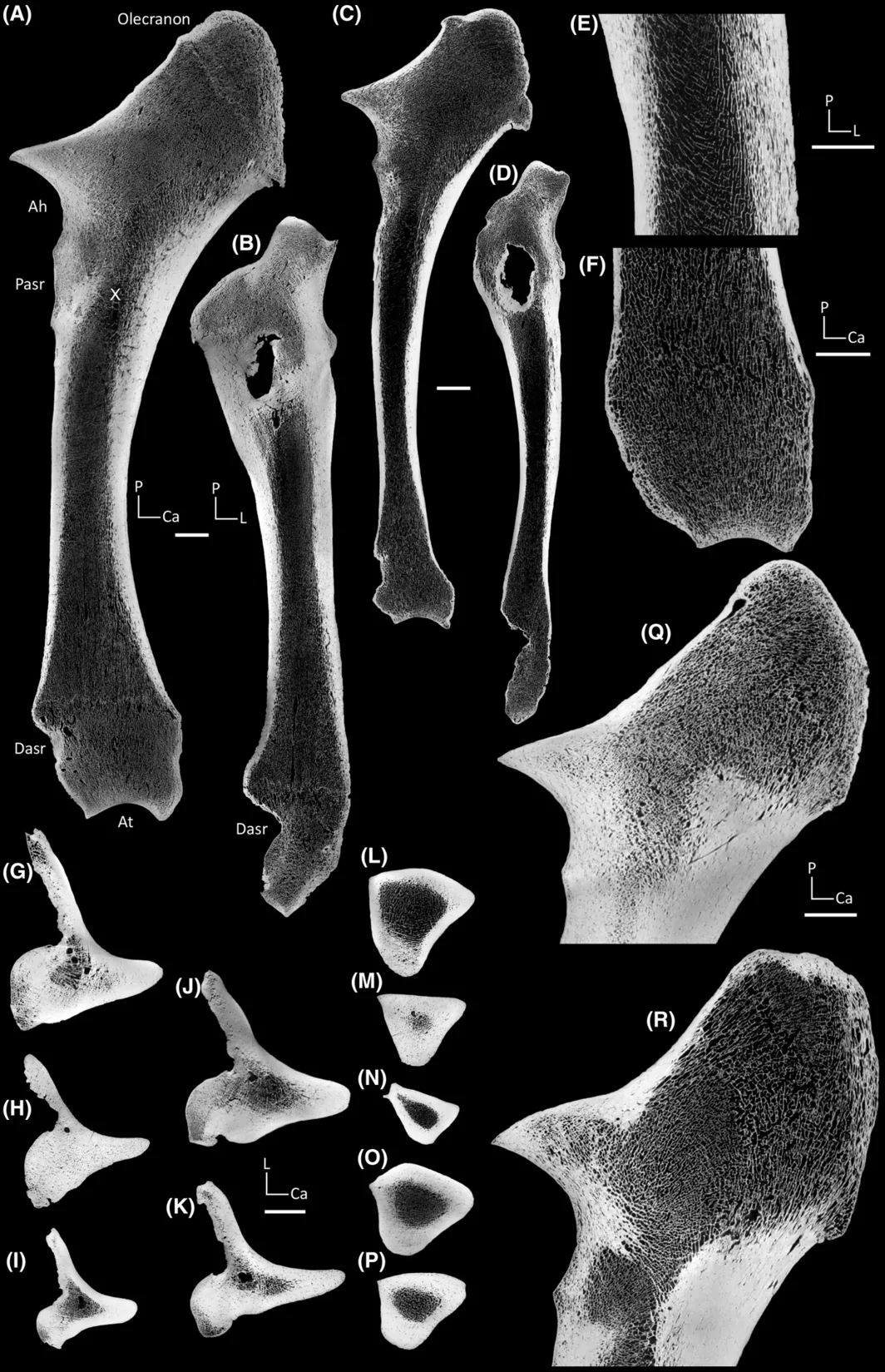
Virtual sections of the ulnae. (A–D) Sagittal (A, C) and coronal (B, D) sections of *R. unicornis* NHM ZE 1961.5.10.1 (A, B) and *D. sumatrensis* RBINS 1204 (C, D). (E) Zoom on the centre of the diaphysis of *C. simum* NMB 8029, coronal section. (F) Zoom on the distal epiphysis of *C. simum* NMB 8029, sagittal section. (G–P) Transverse sections at the growth centre (G–K) and at mid‐diaphysis (L‐P) of *C. simum* NMB 8029 (G, L), *D. bicornis* RBINS 9714 (H, M), *D. sumatrensis* NHM ZE 1948.12.20.1 (I, N), *R. unicornis* NHM ZE 1961.5.10.1 (J, O) and *R. sondaicus* RBINS 1205F (K, P). (Q–R): Sagittal sections at the olecranon of D. *bicornis* MNHN ZM‐AC‐1944‐278 (Q) and *C. simum* NMB 8029 (R). Scale bars are two centimetres. Ah, articular surface for the humerus; At, articular surface for the triquetrum; Ca, caudal; Dasr, distal articular surface for the radius; L, lateral; P, proximal; Pasr, proximal articular surface for the radius. X (in D) indicates the approximate position of the growth centre.

Again, the bones are entirely filled with spongiosa. Trabecular organisation presents a simple pattern. At the olecranon, in sagittal view, trabeculae in the caudal border are highly anisotropic, emerging from the thick cortex in the distocaudal part of the olecranon and then following the border of the olecranon up to the tip (Figure 9Q,R). In the cranial border, a similar arrangement is observed, with trabeculae oriented along the border. Both sets of trabeculae join and cross at the tip. This arrangement is clearly visible in *C. simum* and *D. sumatrensis*, but less marked in the other species. In *R. unicornis* in particular, the caudal trabeculae are highly anisotropic in the most caudal part of the olecranon, next to the thick cortex, but less so in the proximal part. Under the contact with the humerus, *C. simum* and *D. sumatrensis* present highly anisotropic trabeculae with high BVF, oriented perpendicular to the curved surface of the contact area (Figure 9r). This arrangement is less marked in *Rhinoceros*, and not visible at all in *D. bicornis*. *D. bicornis* presents a very high trabecular BVF in the olecranon. *C. simum* present sparser and slightly thicker trabeculae than *Rhinoceros* and *D. sumatrensis*, which all present a very tight spongiosa in the olecranon.

All species but *D. bicornis* present an area of low BVF in the upper part of the diaphysis, where the medullary cavity would lay in most terrestrial ungulates, measuring one‐third to one half of the length of the diaphysis (Figure 9A–D). Trabeculae become denser and more anisotropic when moving closer to the cortical bone, and in the distal part of the diaphysis and the epiphysis. Overall, trabeculae in the diaphysis and distal epiphysis are highly anisotropic, oriented along a proximodistal axis. In coronal sections, at mid‐diaphysis a group of trabeculae go from the medial border towards the lateral border, following a path that is at first more downwards but quickly curves towards the lateral direction (Figure 9E). This arrangement is especially visible in *C. simum*, and to a lesser extent in *R. unicornis*; it is not present in *D. sumatrensis*. Opposite to this arrangement, very close to the lateral border, is a set of trabeculae that are much more vertically oriented. More distally in the diaphysis, trabeculae are almost entirely vertical in both coronal and sagittal views, going from the contact area with the triquetrum to the thick cortical bone of the diaphysis (Figure 9F). In *C. simum* and *D. bicornis*, in coronal view, in the metaphysis, trabeculae are slightly oblique, much as was observed in the distal radius for all species. *D. bicornis* presents a higher trabecular BVF than the other species. Overall, trabecular BVF increases progressively, going from the middle of the diaphysis to the contact area for the triquetrum. Only *D. bicornis* is an exception, having a trabecular bone in the diaphysis that is as dense or even denser than that of the distal epiphysis.

#### 3.3.2 3D cartographies of the ulna

The ulna of *C. simum* that we studied in 3D presents a more complex pattern of distribution of anisotropy and BVF than the radius (Figure 10A). We can identify three patterns. As usual, ROIs with low anisotropy always have a relatively low BVF and are probably subject to relatively low forces (Table 4). These are located mainly in the centre of the bone, both in the diaphysis and epiphyses. Again, ROIs with high BVF always have a high anisotropy and are located mainly on the periphery of the trabecular bone space, close to the cortex. Those are probably subject to the most intense forces. In addition, many ROIs present high anisotropy and low BVF and are located in between the others, with a lot of them in the olecranon. They are likely subject to highly directional forces that are not intense enough to require a great increase of BVF.

**FIGURE 10:**
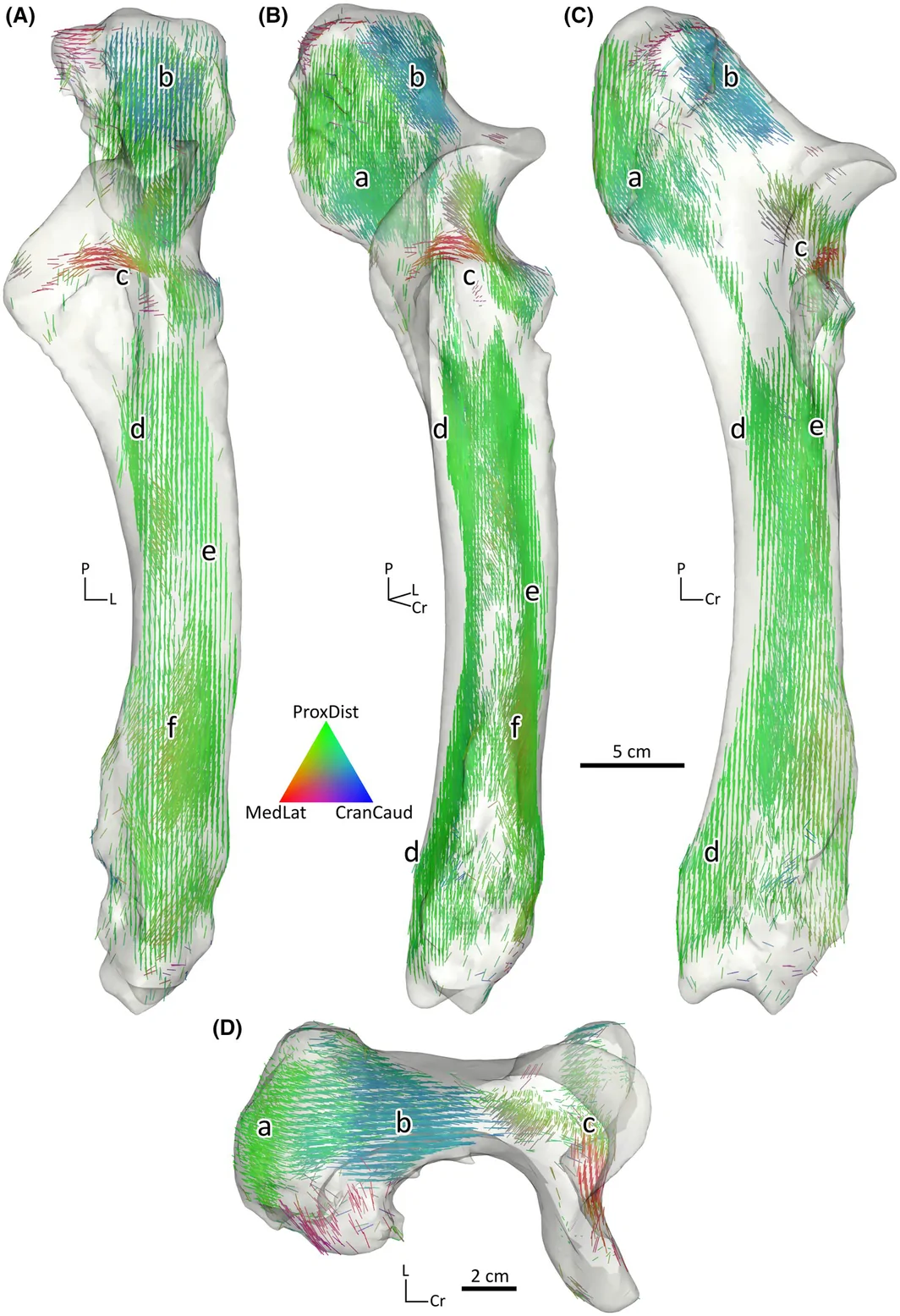
Description of the microanatomy of the ulna in *C. simum*. 3D cartographies of the 25% most anisotropic trabecular ROIs, in cranial (A), craniomedial (B), medial (C) and proximal (D) views. ROIs are represented as vectors pointing in the direction of the anisotropy and are additionally colour‐coded according to their direction. Lower case letters indicate sets of anisotropic trabeculae described in the main text. Cr, cranial; L, lateral; P, proximal.

Trabecular anisotropy in the ulna seems mainly related to the great force exerted by the *triceps brachii*, which pulls the olecranon in a cranioproximal direction (see Figure 4; Table 4). As observed in the sections, the olecranon presents two sets of anisotropic trabeculae. Set ‘a’ (Figure 10B–D) is composed of trabeculae located on the caudal border of the olecranon and is roughly parallel to this border. They answer to the tension directly produced by the *triceps* and merge into cortical bone when it becomes thicker near the growth centre. Set ‘b’ is located on the cranioproximal border of the olecranon, with trabeculae parallel to that border again. They answer to the compression produced in reaction to the tension exerted by the *triceps*. Near the contact with the humerus and radius is a group of high‐anisotropy ROIs (set ‘c’ in Figure 10) with varying orientation. Some of them are directed towards the contact, probably aiding in dissipating the forces; some are parallel to the border of the contact. In the diaphysis of the ulna, anisotropic trabeculae appear again on the caudo‐medial border when the cortex becomes thinner and continue until the distal epiphysis (set ‘d’); these likely answer still to the pull of the triceps brachii spreading distally, but also potentially to joint reaction forces at the wrist. Another set (‘e’) of proximodistally anisotropic trabeculae is located on the craniolateral border. The separation between these two sets is easily visible when looking at the bone in craniomedial view (‘d’ and ‘e’). Set ‘e’ is probably loaded in compression by joint reaction forces. Inside set ‘e’ is a group of trabeculae (‘f’) that are not proximodistally oriented, but instead oriented towards the distal contact with the radius, probably answering to contact forces between those bones and/or to ligament stretch at the junction.

As for the radius, very few interspecific differences are observed. Sets ‘a’ and ‘b’, in the olecranon, are identical in all specimens, although they are both split in two by the epiphyseal line in the subadult *R. sondaicus*. In Asian rhinos, the cortex is usually thinner near the growth centre. As such, sets ‘a’ and ‘d’ are in continuity, forming an uninterrupted large set of trabeculae running through the caudal border of the entire bone, parallel to it (Figure 11). This is particularly marked in *R. unicornis* (Figure 11A). Set ‘c’, near the contact area for the humerus, is similar in *C. simum* and *R. sondaicus*, but in the other species, its trabeculae are more vertical, still parallel to the contact surface, and separated in lateral and medial sets, the latter being directed towards the contact with the radius. Those trabeculae are even more vertical in *D. sumatrensis*, probably due to its ulna being much less mediolaterally expanded. Right below the base of the olecranon, *D. sumatrensis* and *R. sondaicus* present a craniocaudal set of trabeculae going from the caudal to the cranial border of the bone. Set ‘f’, anisotropic trabeculae going towards the distal contact with the radius, is not visible in any other specimen than our *C. simum*. Those trabeculae are actually present in *R. sondaicus* and *D. bicornis*, but are not among the 25% most anisotropic and are thus not displayed.

**FIGURE 11:**
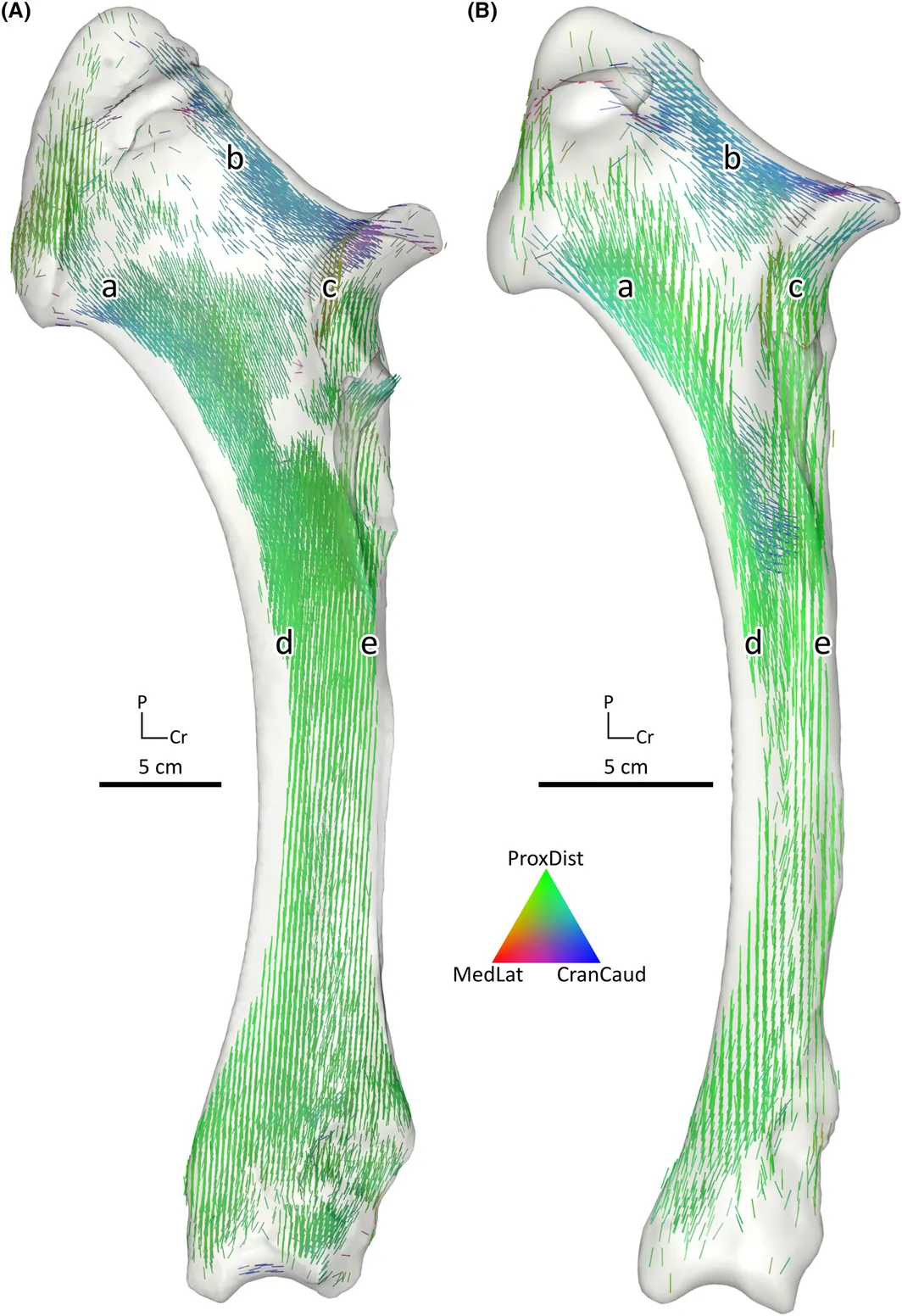
Comparisons of the microanatomy of the ulna between selected specimens. The 25% most anisotropic ROIs are displayed as vectors pointing in the direction of the anisotropy, coloured according to their direction as in Figure 3. (A) *R. unicornis* (B) *D. sumatrensis*. Medial view. Lower case letters indicate sets of anisotropic trabeculae described in the main text. Cr, cranial; P, proximal.

## 4 DISCUSSION

### 4.1 Common adaptations to high magnitude forces

#### 4.1.1 Cortical thickness in the diaphysis

All rhino forelimb long bones are characterised by thick cortex in the diaphysis. That great thickness increases the surface area on which the forces are spread through their limbs and thus decreases the stresses, presumably bringing them below the failure strength of rhino bones with a reasonable safety factor (Biewener, 2005; Currey, 2002; Currey & Alexander, 1985). In all bones, cortical thickness is not homogeneous across the diaphysis, being maximal around the growth centre and gradually reducing further along the diaphysis, which gives the medullary space a distinctive hourglass shape. This arrangement could be due to a thickening where the forces are the highest under loading, to reduce stresses where it is the most needed. Alternatively, this could be due to bone growth, the growth centre being where bone is first deposited during growth and thus where the inhibition of bone resorption can engender the hourglass shape (like in the sea otter; Houssaye & Botton‐Divet, 2018). These hypotheses are not mutually exclusive, so that the position of the growth centre, variable between bones and taxa in mammals (Houssaye & Prévoteau, 2020), could be linked to the functional need of having a thick cortex where it is biomechanically most needed. Further away from the growth centre, rhinos appear to rely on increased trabecular BVF and anisotropy; for instance, in the diaphysis near the cortex, it is often highly proximodistally anisotropic, and thus probably aligned with the main direction of the forces going through the bone. The fact that this arrangement of increased cortical thickness near the growth centre is less marked in *D. sumatrensis*, the lightest species, supports the hypothesis that this arrangement is linked with high body mass support in heavy rhinos, as recently suggested by a higher strength with increased cortical thickness for a rhinoceros humerus subjected to compressive load (Etienne et al., 2025).

#### 4.1.2 Force transmission at contact areas

As in all mammals, to our knowledge, compact bone at contact areas is relatively thin and much thinner than in the diaphysis (Bader et al., 2022, 2025). Thick bone at contact areas would, because it is too stiff, induce more stresses to the cartilage layers between bones (Currey, 2002). We do still observe a thickening of the cortex in some areas (e.g. in the proximal radius, at the medial part of the contact with the humerus), but it remains far thinner than in the diaphysis, and thin enough to allow some compliance. Trabecular bone in contact areas must still be highly resistant, which is usually achieved through a specific pattern: high BVF and moderate anisotropy in the epiphysis, moderate BVF and high anisotropy in the metaphysis (see Figure 2E,G), although this pattern is much less marked in *D. sumatrensis* which presents more homogeneous trabecular bone. As joint reaction forces can come from various directions depending on the position of each limb segment relative to one another during locomotion, such an arrangement appears well suited to cushion forces from various directions in the epiphysis and then transmit them efficiently to the thick cortex in the diaphysis via highly anisotropic trabeculae in the metaphysis. This arrangement likely originates from differences in development, here co‐opted to efficiently resist high forces. Indeed, bone in the metaphysis is formed by the primary ossification centre, where chondrocytes (cartilage generating cells, which generate the matrix from which bone tissue will be formed during endochondral ossification) tend to be organised in columns. Chondrocytes of secondary ossification centres, in the epiphyses, are more randomly organised (Carter & Beaupré, 2000).

#### 4.1.3 Force transmission at muscular attachment areas

The 3D quantification of trabecular anisotropy and bone volume fraction in the three bones of the five species of rhinoceroses confirm and illustrate the great complementarity between trabecular and cortical bone. Both muscular forces and joint reaction forces, when applied on the epiphyses, appear to be associated with sets of highly anisotropic trabeculae that carry the forces to cortical bone. This great anisotropy gives trabecular bone a greater stiffness in the appropriate direction (Currey, 2002; Kivell, 2016), and thus strength, resisting breakage. Cortical bone has an even greater stiffness and strength and can distribute forces over a greater surface, thus lowering the stresses even further. Overall, trabecular bone shows a remarkable adaptation to loads of varying magnitude, orientation and point of application (Etienne et al., 2024). The 3D cartographies, however, highlight the complexity of the distribution of force orientation (i.e. principal stresses) inside the bones. Trabeculae do not seem to directly align with the direction of muscle pull, but instead reorient constantly inside the bones, following principal stresses in tension and compression that result from complex bending loads. Notably, they are often perpendicular to the original pull, as in a cantilever (Li & Chen, 2010; see also Zhang et al., 2023 for distribution of principal stresses in a femur).

Muscular insertions, contrary to joint reaction forces, primarily exert a tension. Their magnitude can rival that of joint reaction forces even in old specimens (Etienne et al., 2024). In the epiphyses, muscles such as the *supraspinatus*, *infraspinatus* and *triceps brachii* seem linked with an anisotropy of the trabeculae, presumably providing a greater stiffness (Currey, 2002) and thus a greater strength in the direction of muscle tension (see caudal olecranon in Figure 9R). In the diaphysis, the *biceps brachii* seems to be associated with a thickening of the cortex in the radius (Figure 6). That thick cortex is, moreover, associated with anisotropic trabeculae oriented in the direction of muscle pull. In the radius, trabeculae in the proximal epiphysis are anisotropic and plate‐shaped, thus providing increased stiffness in both the direction of joint reaction forces and that of the *biceps'* pull. It is likely that muscles inserting on the diaphysis benefit from the close proximity of the primary growth centre and the associated thick cortex. Some muscles delivering a high tensile force, such as the superficial pectorals, insert on an even thicker cortex and do not seem to impact the underlying trabeculae.

### 4.2 Functional adaptation of the limb bones

Of the three bones studied here, the humerus bears the most diverse muscular forces and the highest total forces overall (Etienne et al., 2021, 2024). It notably shows highly anisotropic trabeculae under the bicipital groove, probably in reaction to the compression generated here by the *biceps brachii* tendon when the muscle contracts (Figure 2B,D). That such an anisotropy is so visible, for a muscle that merely wraps around the bone, is coherent with a quasi‐constant activation of the *biceps* due to its role as a shoulder extensor and overall stabiliser of both the shoulder and the elbow (Etienne et al., 2024). In the middle of the bone, above the growth centre, is the region of the lowest BVF and anisotropy. It thus seems that even if the medullary cavity is filled, the region subject to the lowest forces remains approximately the same. Overall, several microanatomical features indicate that the humerus bears more weight in its medial part: the cortex is thicker medially than laterally; the set of anisotropic trabeculae in the humeral head in positioned more medially (set ‘a’ in Figure 3); the medial part of the articular trochlea presents denser and more anisotropic trabeculae. The 3D analysis has shown that the great homogeneity of the orientation of the trabeculae underneath each articular surface of the humerus is consistent with the idea that the proximal forelimb does not move much during the stance phase of locomotion, merely supporting the body (Etienne et al., 2021, 2024). Trabecular bone under powerful muscles is usually clearly anisotropic and indicates the location of the muscular insertion. The location of the trabeculae answering to the superficial pectorals (set ‘f’ in Figure 3) thereby helps determine the exact location of those muscles' insertion, since it is not visible on the external morphology of the bone. The relationship between anisotropic trabeculae and muscle force is, however, far from being straightforward. Muscles in the epiphyses seem to cause greater sets of anisotropic trabeculae, often spreading until they reach the thick cortical bone. The insertion of the *supraspinatus* seems to spread onto the whole craniolateral border of the proximal humerus, but we know from muscle data (Etienne et al., 2021) that this is not the case. One should thus be careful when interpretating insertion areas, as stresses (and thus, anisotropic trabecular bone) can spread across large portions of bone far from the insertion area. Some regions also do not present anisotropic trabeculae where they would have been expected based on the musculoskeletal model (e.g. for the deep pectorals and the *teres major* tuberosity (which is also the insertion of the *latissimus dorsi*)), which probably indicates a lesser frequency of use for those muscles. The deep pectorals insert on a very well‐defined region (the lesser tubercle), but their great development might have more to do with providing the muscles with a longer lever arm than resisting muscular forces, which would explain why no concentration of anisotropic trabeculae is observed there. To a lesser extent, the same might be said of the *deltoideus* muscle, as there is only very few anisotropic trabecular bone in this area in most rhinos studied. These muscles might be used for additional stability during locomotion, but not during standing. The *latissimus dorsi* is essential for limb retraction and so is expected to be used frequently, but not as frequently as muscles active when standing still. Furthermore, it inserts on an even thicker cortex than the superficial pectorals, so that anisotropic trabecular bone might not be necessary to resist its force.

The radius microstructure seems to be primarily linked with weight bearing, resulting in homogeneous trabecular bone with trabeculae oriented approximately proximodistally (Figure 6). In its proximal third, it seems to bear more weight on the medial side, like the humerus, as the cortex tends to be thicker medially, except in *R. unicornis* where cortical thickness is symmetrical. Mallet et al. (2019) did identify relatively larger articular facets on the medial side of the elbow articulation in heavy rhinos as compared with lighter ones, which is indeed consistent with more forces going through the medial side. Nonetheless, there are more anisotropic trabeculae on the lateral side of the proximal radius than in that of the distal humerus, respective to the medial sides of these bones. This difference could be due to differences in bone orientation, as the radius is much more vertical than the humerus; it thus probably indicates a slight shift in the distribution of the stresses as they go through the elbow, from the medial side to the centre of the bone. This shift is more pronounced more distally in the radius, as cortical thickness and trabecular anisotropy progressively equalise between the lateral and medial sides in the distal two‐thirds of the bone. This is consistent with the contact between the carpus and the radius and ulna appearing parallel to the ground (Etienne et al., 2024; Figure 4). On the sections, trabeculae below the growth centre seem oriented not along the long axis of the bone, but along the vertical axis of the whole limb (Figure 6A,B), which is confirmed from the bone cartographies (Figure 7). This suggests that joint reaction forces in the wrist are not aligned with the bone's long axis, contrary to what was estimated in a standing at rest rhino in Etienne et al. (2024). We hypothesise that the trabecular alignment is useful during impacts, when joint forces become greater and are likely to be more vertical. Perhaps when the rhino is standing at rest, most of the forces go through the caudal border, where the trabeculae and forces at rest are aligned, and when the rhino is moving, more shocks come in a vertical direction in the centre of the bone.

The microanatomy of the ulna also appears dedicated to a main function, that is serving as a long lever arm for the *triceps brachii* (probably along with a small weight‐bearing role; Mallet et al., 2019; Etienne et al., 2024; Figure 9). Indeed, the microstructure reflects the pull on the olecranon by this muscle in the cranio‐proximal direction, generating tension all over the caudal border of the olecranon, and that spreads to the diaphysis and goes down to the distal epiphysis (Figure 9R). That tension is spread first over anisotropic trabeculae that follow the caudal border of the olecranon, and is then transmitted to thick cortical bone at the junction between the olecranon and the diaphysis, where the stresses probably concentrate. It also generates a compression on the cranial side of the olecranon, which is also associated with anisotropic trabeculae (that follow the direction of the cranial border of the olecranon; Figure 9R). Other adaptations on the ulna include anisotropic trabeculae with high BVF near the contact with the humerus, indicating some joint reaction forces passing through, in reaction to the pull of the *triceps* pressing the ulna against the humerus, and probably during locomotion. The arrangement of the trabeculae near the contact with the carpus is similar to that of the contact area in the radius, supporting the hypothesis that the ulna retains some weight‐bearing role (Etienne et al., 2024). The proximal arrangement of the ulna is very similar to that of the horse, although the ulna merges with the radius in the latter, so that its distal part is completely absent. The independent ulna in rhinos may allow for much greater forces to be exerted by their *triceps* (Etienne et al., 2021) and may also indicate that the ulna retains a weight‐bearing role, even if not as much as the radius.

### 4.3 Differences between rhinoceros species

Sumatran rhinos are the lightest species (600–950 kg; Dinerstein, 2011) and present several microanatomical characteristics coherent with their relatively low body mass. Their cortex is thinner relatively to their length (except in the ulna), and they usually retain an open medullary cavity. These microanatomical features are consistent with their long bones being slenderer than in the other rhinos (Mallet et al., 2019, 2020). However, the slenderness of these bones results in relatively smaller cross‐sectional areas, so that compactness around the growth canter is not necessarily lower in this species (Figures 2K–O, 6E–I, and 9L–P). We could have expected *D. sumatrensis* to also present a lower anisotropy than the other rhinos, but this does not appear to be the case. This species being the only one possessing diversely oriented trabeculae in the humerus underneath the contact area at the medial trochlea, it leads us to hypothesise that its elbow is more mobile, with forces coming from a greater variety of directions depending on the angle of the elbow, due to *D. sumatrensis* being lighter and perhaps less constrained to a particular position of the forelimb. *D. sumatrensis* is also the species that lives in the most closed environment, a tropical rainforest, and it is a good climber. It may have to deal with more obstacles in its natural habitat, leading to a more varied use of its forelimb resulting in forces coming from more diverse directions.

*D. bicornis* is the second lightest species (800–1300 kg), being only ~300 kg heavier than *D. sumatrensis*, twice as light as *C. simum* and *R. unicornis*. However, it presents the thickest cortex and densest spongiosa, as previously observed (Houssaye et al., 2016). Functional requirements demanding stronger bones for a lower mass and thus differing significantly from those of other rhinos are hard to imagine, as are developmental constraints. Phylogenetic heritage is unlikely as well, as black rhinos' fossil relatives are not particularly heavy (Mallet et al., 2022). Our sample is only of four individuals, including two coming from a circus, but all of them are denser than other rhinos, although perhaps the great density of the bones of *D. bicornis* is due to genetic processes, not plastic adaptations. One hypothesis is courtship, which is violent in perissodactyls in general and particularly violent in black rhinos (up to 50% of males and 30% of females of that species die of courtship‐related injuries; Dinerstein, 2011). However, three of our four individuals were captive bred, where violent courtship is likely not frequent. *D. bicornis* could also perhaps be faster than other rhinos and thus necessitate stronger bones (it was recorded at 45 km/h, whereas *C. simum* was only recorded at 27 km/h; Garland, 1983; Alexander & Pond, 1992), but data on maximal rhino running speed are extremely scarce, and heavier bones, particularly distal ones, are harder to move at fast speeds. Moreover, it is far from obvious that a faster running would engender the functional requirement of much thicker bones. Again, captive specimens, particularly in circuses, are unlikely to often run fast. Black rhinos do not swim (Dinerstein, 2011), thus increased bone density to decrease buoyancy is not an option. The great density of black rhino bones remains puzzling. It can also be noted that *D. bicornis* presents a high BVF but a low anisotropy, perhaps relying more on BVF than anisotropy to support its weight. Perhaps, its potential heritage of more robust bones than would be necessary (both in terms of outer morphology and bone volume fraction) would mean it experiences lower stresses that generate less anisotropic trabeculae. The trabeculae of the humeral head and medial trochlea are more inclined—relative to the proximodistal axis—in *D. bicornis*. Since we expect these trabeculae to align with the direction of the joint forces, at least in the part closest to the contact area. It could thus mean that the joint forces are more inclined in *D. bicornis*. Perhaps, the humerus itself is more inclined in this species, but this is difficult to quantify sufficiently precisely without radiographies of a whole standing rhino.

For the other species (*R. unicornis*, *R. sondaicus*, *C. simum*), interspecific differences are limited, except for some individuals, likely pathological (see Supplementary Data S5). Two of those species, *C. simum* and *R. unicornis*, are of very similar body mass (from 1350 to 2100 kg). The other, *R. sondaicus*, is the third heaviest, even though to a lower extent (1200–1500 kg); nonetheless, it is phylogenetically very close to *R. unicornis*, and such phenotypical similarities might also result from those phylogenetic affinities.

Despite their higher body mass (1350–3500 kg for *C. simum*, 1350–2100 kg for *R. unicornis*; Dinerstein 2011), neither *C. simum* nor *R. unicornis* show the highest trabecular density. This is surprising since we would expect more forces to pass through their limb bones, and thus, a higher BVF to be needed. Heavy rhinos present many adaptations to weight support in terms of bone shape already (Mallet et al., 2019). It is possible that these adaptations are sufficient to resist the intense loads they bear, without need for further microanatomical adaptations. These rhinos are also of varying limb proportions, with *C. simum* presenting relatively shorter limbs (body length to shoulder height ratio: 2.3 in *C. simum*, 1.8 in *R. unicornis*, 2.0 in *R. sondaicus*; Dinerstein, 2011). Their bone shape also varies, particularly between *Rhinoceros* and *C. simum*, but the musculature of *R. unicornis* and *C. simum* is quite similar (Etienne et al., 2021), which means that the forces experienced by the bones must be similar as well, which would explain their similar microanatomy. One slight possible difference is on the aspect of the spongiosa, which seems tighter in both *R. unicornis* and *R. sondaicus*, making trabecular anisotropy patterns less obvious. Another difference of note is the absence of an anisotropic trabecular set underneath the pectoral crest in our studied specimen of *R. sondaicus* (set ‘f’ in Figure 5A,B). This is surprising and could be due to our *R. sondaicus* being a subadult. Perhaps, those trabeculae develop later in life, when the animal reaches its adult body mass that necessitates a stronger adduction from the pectorals to keep the limb firmly against the body. In general, *C. simum* and *D. sumatrensis* present a clearer pattern of anisotropy than the others. It seems, however, more likely that this difference is due to the fact that almost all of our *Rhinoceros* specimens are subadults (see Supplementary Data 5.2). The fact that *R. unicornis* and *D. sumatrensis* have more anisotropic trabeculae in the lateral part of the distal end of the radius (whereas lateral and medial parts are quite balanced in the other species) probably reflects higher forces coming from the lateral part of the wrist in these individuals.

### 4.4 Differences with other large vertebrates

Horses, which generally weigh between 380 and 600 kg (Bongianni, 1988), present a similar general microanatomy in their limb long bones as rhinos, although with a thinner cortex and more limited extension of trabecular bone in the medullary region. This is consistent with the fact that horses and rhinos show relatively similar musculature and locomotor habits (Etienne et al., 2021). Comparing the organisation of trabeculae in the humerus of rhinos to the structural diagram of the humerus of the horse proposed by Barone (1999), points to similarities, notably underneath the contact surfaces, the bicipital groove, the greater tubercle (*supraspinatus* insertion) and the lateral epicondyle (*ulnaris lateralis*). Anisotropic trabeculae are thus found at the same places in rhinos and horses (Barone, 1999), where high forces are applied, whether it be in tension (e.g. humeral tubercles, caudal tuber olecrani) or in compression (e.g. articular surfaces, bicipital groove, cranial tuber olecrani). However, we do not observe many anisotropic trabeculae underneath the lesser tubercle of rhinos, whereas in horses, trabeculae underneath the greater and lesser tubercle seem similar, extending far in the diaphysis. This could be linked to the fact that the *supraspinatus* of the horse inserts on both the greater and lesser tubercles, whereas in rhinos it only inserts on the greater one. That muscle would thus have to be resisted by greatly anisotropic trabeculae underneath both tubercles in the horse, and only underneath the greater in rhinos. A comparison with tapirs would be most interesting, as their muscular arrangement regarding the insertion of the *supraspinatus* appears especially variable, with either a lone insertion on the greater tubercle or a double insertion as in horses (MacLaren, 2023; MacLaren & McHorse, 2020). The medial epicondyle of the horse also seems to present more anisotropic trabeculae than in rhinos; on this point, *D. sumatrensis* is close to the horse, which might be due to its lower body mass. In horses, only the proximal part of the ulna can be compared to that of rhinos, since the distal part is absent. The proximal parts are very similar. The fusion of the ulna with the radius in horses means that, at one point, the tension produced by the *triceps* on the tip of the ulna must be transferred to the radius. The separation of the radius and ulna is likely advantageous for rhinos, as it provides a larger surface for the forces to dissipate. This results in a situation in rhinos where the radius is specialised for body weight support and the ulna is specialised for muscle tension resistance. Trabecular BVF is the highest at contact areas and the lowest in the core of all the bones in both rhinos and horses. The main differences are in the cortical thickness, which is relatively much thinner in horses, and homogeneous throughout the diaphysis, and in the presence of a large medullary cavity in horses. In this regard, horses are close to Sumatran rhinos. Trabecular bone is also denser, with trabeculae that are more tightly packed in rhinos.

*Hippopotamus amphibius* is the closest land mammal to large rhinos in terms of body mass (1000–4500 kg; Lewison, 2011), but differs markedly in its habitat, being a semi‐aquatic species that often ‘walks’ on the bottom of water bodies (Lewison, 2011). Asiatic rhinos are good swimmers but spend much less time in the water than hippos, and to our knowledge, do not dive. Only partial sections of the humerus (and femur) of *Hippopotamus* are available (Houssaye et al., 2021); cortical thickness is similar between *H. amphibius* and rhinos (except *D. sumatrensis*), although hippos present a more homogeneous cortical thickness along the diaphysis. Rhinos and *Hippopotamus* are similar in the filling of their medullary cavities by spongious bone. Sea otters incidentally present a strikingly similar microanatomical pattern to large rhinos in their humeri, although bone shape is clearly different (compare Figure 1B with Figure 1H in Houssaye & Botton‐Divet, 2018). Indeed, a medullary cavity filled with trabeculae increases bone strength, but also reduces buoyancy for semi‐aquatic animals. Heavy weight and semiaquatic habits can lead to similar bone microanatomical adaptations, and thus, adaptations to one or the other can be difficult to detangle (Houssaye, 2009; Houssaye et al., 2016, 2021; Laurin et al., 2011).

The heaviest land mammals on Earth today are elephants. As is usual for heavy mammals, their medullary cavity is filled by trabeculae, and apparent BVF is highest underneath contact areas (Bader et al., 2025; Nganvongpanit et al., 2017). Elephant bones seem to present the same trabecular pattern as in heavy rhinos: higher BVF in the epiphysis, higher anisotropy in the metaphysis. However, their cortex appears relatively thin compared to that of rhinos, and much more tubular, with no thickening around the growth centre, which might be due to their more vertical limb posture and their absence of galloping or trotting gait, leading to less intense bending moments on their bones.

### 4.5 Benefits of the 3D approach

Exploration in 3D confirms observations from 2D sections. It was important to check that the observations from the cartographies matched those from the 2D sections. Indeed, defining the size of regions of interest (ROIs) can always lead to a bias between the need to include a sufficient number of trabeculae and the need to avoid a region too large and heterogeneous, at the risk of rendering the average values of BVF and anisotropy irrelevant. Moreover, the inclusion of different sets within a single ROI could artificially lead to low estimates of anisotropy, preventing these sets from being revealed. Finally, the cartographies only compute two parameters (bone volume fraction and anisotropy), but others are of interest as well, such as trabecular shape (plates or rods), connectivity and tightness of the trabecular mesh. The correspondence observed here between the cartographies and the 2D sections thus suggests that these maps can be relied upon. These cartographies enabled us to identify new sets of anisotropic trabeculae in various directions, which respond to at least five groups of muscles (*supraspinatus*, *infraspinatus*, superficial pectorals, *ulnaris lateralis*, digital and carpal flexors), all presenting an antigravity and/or stabilisation action. We could confirm that the radius is primarily optimised to support body weight, acting as a pillar, with highly anisotropic trabeculae. The 3D analysis allowed us to elucidate the orientation of the trabeculae in the distal part of the radius, showing that trabeculae along the caudal border of the bone are aligned with both the long axis of the bone and the joint reaction forces at rest calculated in Etienne et al. (2024), whereas trabeculae in the centre of the bone are more aligned with the vertical axis of the whole limb, perhaps resisting shocks that might arise during movement. The ulna is primarily optimised to resist the pull exerted by the *triceps brachii*, and most of its trabeculae answer to that force. Some, in the cranial border of the bone, likely transmit some joint reaction forces from the wrist to the elbow.

Our method has the advantage of allowing very dense visualisations of anisotropy in 3D (up to 3.7 million ROIs), leading to very precise descriptions. It is also very flexible: the final data set can indeed be filtered according to ROI position, BVF, degree of anisotropy and direction of anisotropy, or any combination of these variables. Colouration of anisotropy vectors according to BVF allows easy visualisation of how these two parameters covary inside a bone, and thus a better representation of the 3D structure. The comparison of the 3D cartography of the humerus with the virtual sections shows that many sets of anisotropic trabeculae were not visible on the virtual sections. Notably, set ‘d’ (very large set of proximodistally anisotropic trabeculae in the craniolateral part of the proximal humerus; Figure 3) is barely visible in the virtual sections and set ‘h’ (proximodistally anisotropic trabeculae in the lateral epicondyle) is not visible at all, even though those cover a large part of the bone, where forces exerted by capital extensor muscles (*supraspinatus*, *ulnaris lateralis*) converge. Sets ‘f’ (beneath the pectoral crest), ‘i’ (medial epicondyle) and ‘k’ (distal extremity of the lateral epicondyle) are not visible either. To be able to view them, all in virtual sections would require several additional sections, at different positions in the bones. Even when sets of anisotropic trabeculae are seen on the virtual sections, the 3D direction of the anisotropy is hard to determine, since the sections are in 2D. The use of 3D cartographies thus allows much greater precision in the characterisation of trabecular bone anisotropy. The 3D approach can refine hypotheses and give important precisions in certain areas. We were able to correlate BVF and anisotropy (Supplementary Data S6), and confirm that areas with higher BVF are usually more anisotropic, and vice versa, and that they are located underneath regions subject to high forces according to the musculoskeletal model described in Etienne et al. (2024).

Overall, we think this method could be a great complement to other methods for estimating the location and maximal strength output of muscles in extinct species, which can be complicated (Bader et al., 2022; Bates et al., 2021; Bishop et al., 2021; Demuth et al., 2022). However, to do this, one needs a clear view of what kind of muscle is associated with what kind of trabeculae in a broader sample, more representative than rhinos. One also needs to keep in mind that not all physical characteristics necessarily result from adaptation, and that developmental and structural constraints may very well alter the phenotype of an animal (Gould et al., 1979; Kivell, 2016; Seilacher, 1970). For instance, the difference usually observed between trabeculae in the epiphysis and metaphysis, with higher BVF but lower anisotropy in the epiphysis, likely results from differences in organisation of the growths centres in the diaphysis and epiphyses (Carter & Beaupré, 2000). That difference might have been co‐opted to serve a mechanical purpose, for example, denser trabeculae in the epiphyses could resist forces from various directions more easily thanks to higher BVF, and transmit them in one main direction through highly anisotropic trabecular bone in the metaphysis, but it may also be a constraint that rhinos have to deal with.

## 5 CONCLUSION

We presented here the first study focusing on the adaptive features of cortical and trabecular bone linked to heavy weight support and locomotion, in the forelimb of rhinoceroses, with an unprecedented sample size for animals this large (69 bones entirely imaged using X‐ray CT). We also used a novel approach to visualise in 3D the distribution of anisotropy and bone volume fraction inside complete bones and compared all these data with muscular dissections and a musculoskeletal model. Overall, in accordance with our hypotheses, all rhinoceroses but the lightest present very dense forelimb long bones, filled with trabecular bone. This increases the total surface of bone tissue the forces are applied to and thus decreases the stresses experienced by bone and the likelihood of fracture. *Dicerorhinus*, the lightest species, presents a different microanatomy from the others, with a thinner cortex and a lower apparent bone volume fraction. Areas with higher BVF are usually more anisotropic and vice versa and appear to be found beneath regions subjected to high forces according to the musculoskeletal model. As expected, trabeculae align with the loads they experience. This is particularly true of contact areas at the articulations, where we highlighted thick and anisotropic trabeculae, tightly packed, that can resist the joint reaction forces and transmit them to the thick cortex in the diaphysis. Our 3D analyses allow us to confirm and illustrate the great complementarity between cortical and trabecular bone. Overall, with few exceptions, anisotropic regions are similar between all species and are even similar to those observed in horses, suggesting that they all present a similar distribution of forces due to their cursorial adaptations, even if the intensity of the forces varies greatly. Extreme compactness was observed in *Diceros*, but remains unexplained. Overall, intraspecific variation is very limited, likely because the high forces due to body mass restrict the microanatomy of rhinoceroses to one optimal condition. Our (limited) comparisons with horses showed that they present more adaptations to cursoriality, such as more anisotropic trabeculae below protractor muscle insertions and at ligament insertions, presumably because they need strong ligaments to stabilise their joints when running. Conversely, rhinos are subject to more intense forces at the joints and at extensor muscle insertion areas, which could overtake anisotropic trabeculae due to flexor muscles or ligament insertions. Rhinos, with their cursorial ancestry and flexed limbs, necessitate stronger bones with relatively thicker cortices than elephants and sauropod dinosaurs that can compensate with, for instance, their columnar limb posture. Beyond a thorough understanding of the microanatomical adaptations of rhinoceros forelimb bones, in regard to their heavy weight and locomotor abilities, this study highlights the great correspondence between microanatomical features and forces acting on bones, and thus, the strong potential of studying detailed bone microanatomy to enhance our understanding of musculoskeletal adaptation in extinct taxa and thus our palaeoecological inferences.

## FUNDING INFORMATION

This work was supported by European Research Council (715300).

## Supporting information


**Supplementary Data S1** X‐ray micro‐CT acquisition and reconstruction protocol.

**Supplementary Data S2** Training dataset used to train a classifier for one bone.

**Supplementary Data S3** Example of the computation of the regions of interest (ROI) in the bones, and their correction for artificially low BVF at the edges of the bones.

**Supplementary Data S4.** Python and R code used for the study.

**Supplementary Data S5.** Intraspecific variations.

**Supplementary Data S6** – Interspecific variations of quantitative parameters.

**Supplementary Data S7.** Microanatomy of the humerus in C. simum.

**Supplementary Data S8.** 2D histograms showing the correlation between bone volume fraction and anisotropy.

## Data Availability

The data that support the findings of this study are openly available in not available at the moment (hacking) at https://3dtheque.mnhn.fr/.
