# Supplementary material for: How do forelimb long bones adapt in rhinoceroses? An in‐depth examination of their microanatomy
Source: J Anat. 2026 Jun 1;249(6):1209–38. doi: 10.1111/joa.70180 (PMC13398847; doi:10.1111/joa.70180)
Supplement: Supplementary file 4 [file JOA-249-1209-s004.docx]

Supplementary data 3 – Example of the computation of the regions of interest (ROI) in the bones, and their correction for artificially low BVF at the edges of the bones.

Fig. S3.1. Illustration of ROI position and artefact correction. A. Slice of segmented bone (in white on grey background) with one ROI represented as a blue circle. The centre of each ROI on the slice is represented as a red dot. This representation is in 2D, but ROIs are 3D spheres. B, C. Cartography of BVF in a transverse slice of a tibia before (B) and after (C) correction for the artefactually low BVF at the edges of the bone.

Table S3.1. Trabecular thickness and spacing inside the humerus of each of our species, and resulting region of interest (ROI) spacing and radius used for the cartographies of bone volume fraction of anisotropy in all the bones of that species. All values are in millimetres.

| Species | Trabecular spacing | Trabecular thickness | ROI spacing | ROI radius |
| --- | --- | --- | --- | --- |
| *C. simum* | 0.767 | 0.452 | 1.219 | 2.438 |
| *D. bicornis* | 0.682 | 0.561 | 1.243 | 2.486 |
| *D. sumatrensis* | 0.901 | 0.326 | 1.227 | 2.454 |
| *R. unicornis* | 0.666 | 0.441 | 1.107 | 2.214 |
| *R. sondaicus* | 0.659 | 0.441 | 1.1 | 2.2 |
