# Supplementary material for: How do forelimb long bones adapt in rhinoceroses? An in‐depth examination of their microanatomy
Source: J Anat. 2026 Jun 1;249(6):1209–38. doi: 10.1111/joa.70180 (PMC13398847; doi:10.1111/joa.70180)
Supplement: Supplementary file 5 [file JOA-249-1209-s005.docx]

Supplementary data 2 – Training dataset used to train a classifier for one bone.

Fig. S2.1. Manually segmented slice fragments used to train the classifier for one bone of our *C. simum* individual. All the fragments used for this classifier are included in the image. In greyscale are the original images, and in black and white below are the manually segmented versions, with bone tissue in white.
