# Supplementary material for: How do forelimb long bones adapt in rhinoceroses? An in‐depth examination of their microanatomy
Source: J Anat. 2026 Jun 1:10.1111/joa.70180. Online ahead of print. doi: 10.1111/joa.70180 (PMC13398847; doi:10.1111/joa.70180)
Supplement: Supplementary file 1 — Supplementary Data S1 X‐ray micro‐CT acquisition and reconstruction protocol. [file JOA-9999-0-s003.docx]

Supplementary data 1 – X-ray micro-CT acquisition and reconstruction protocol.

Specimens analysed in this study were imaged using X-ray micro-CT at two facilities: at the MNHN’s AST-RX platform (UMS 2700; GE phoenix∣X-ray v∣tome∣xs 240), and at the NHM’s micro-CT laboratory (Nikon HMX 225 ST system). The MNHN GE phoenix∣X-ray v∣tome∣xs 240 allowed for complete imaging of bone without particular protocol: imaging a complete bone required several acquisitions along the vertical axis, and all acquisitions were directly merged together using Phoenix datos|x, resulting in a single image stack dataset representing complete bones.
The Nikon HMX 225 ST system at the NHM had a maximum vertical stroke of approximately 40 cm, which does not allow complete imaging of the bone in a single sequence. The following protocol was used for all specimens imaged at this facility:

- Several bones were bundled together and tightly secured with cling film and tape.
- Each bundle was placed in a bucket, with the proximal heads oriented upwards.
- The sample manipulator’s vertical stroke of approximately 40 cm allowed imaging of the distal two-thirds of the bones by performing a series of two or three acquisitions (each in circular geometry), moving the sample vertically stepwise by about 80 % of the vertical field of view.
- The bundle was then flipped upside down, with the proximal heads facing downwards.
- The proximal parts of the bones were then imaged, completing full characterization of each bone.
- Each bundle of bones was therefore associated with a series of two or three acquisitions for the distal part and one complementary acquisition for the proximal part.
- Tomographic reconstruction was performed for each individual acquisition using CT-agent software (Nikon Metrology, Leuven, Belgium), resulting in 32-bit image stack datasets.
- Using a Python script developed at the NHM, the 3D histograms of all datasets corresponding to a bundle were analysed to define a common windowing for the subsequent conversion from 32-bit float to 16-bit integers (values outside the 0.02 % minimum and 99.98 % maximum percentiles were discarded).
- The distal datasets were directly merged using the NHM Python script, which assessed the vertical overlap of datasets from motor positions and refined the alignment using a least-error approach, adjusting the overlap around the theoretical value. The merged distal dataset and the isolated proximal dataset were both converted to 16-bit integers using identical windowing.
- Finally, the merged distal dataset and the proximal dataset were combined in Avizo 2019.1 (Thermo Fisher Scientific, Hillsborough, OR, USA). Because this second merging step involved both translation and rotation (the specimen having been manually inverted), we used the Geometric Transform / Register Images tool in Avizo 2019.1. Registration was performed in rigid mode and in three dimensions. After registration, the datasets were merged using the Compute / Volume Operations / Merge tool, and the final merged datasets were exported as stacks of 16-bit TIFF files.
