# Supplementary material for: How do forelimb long bones adapt in rhinoceroses? An in‐depth examination of their microanatomy
Source: J Anat. 2026 Jun 1:10.1111/joa.70180. Online ahead of print. doi: 10.1111/joa.70180 (PMC13398847; doi:10.1111/joa.70180)
Supplement: Supplementary file 4 — Supplementary Data S4. Python and R code used for the study. [file JOA-9999-0-s004.docx]

Supplementary data 4. Python and R code used for the study.

**SD4.1. Python code used to recombine the separate vector .csv files into one.** The calculations of anisotropy were done in several time to lower the RAM requirements, and the direction of the anisotropy vector had to be recombined using the following code.

#Author : Cyril Etienne

# This file is used to recombine .csv vector files exported from dragonfly.

# This is only necessary if, because of RAM requirements, the anisotropy algorithm of Dragonfly had to be run on several computation boxes,

# splitting the bone into several anisotropy cartographies.

# It is essential that the segmentated bone stack imported in dragonfly at the beginning is sliced in the same direction (usually, proximodistal)

# as the computation boxes that were created (i.e., the bone was divided into several boxes proximodistally)

# Vector files must be exported from dragonfly, in the order they created , with the name "Vect1.csv", "Vect2.csv" and so on

import os

#USER INPUT

os.chdir('D:/PATH/') #Path to the vector files

slice_size = 154*155 #width times length of an anisotropy slice

nb_fichiers = 10 #number of vector files

cropLength = 405 #number of anisotropy slices desired (corresponding to the number of BVF slices, in proximodistal direction)

print("Opening vector files")

liste_fichiers = []

for i in range(1,nb_fichiers+1):

    chemin = "Vect" + str(i)+ ".csv"

    fichier = open(chemin, "r")

    liste_fichiers.append(fichier.readlines())

    fichier.close()

    print(i)

print("Combining vector files")

header = "Unit,PosX,PosY,PosZ,VectX,VectY,VectZ\n" #First line of the CSV final file

liste_lignes = [header]

liste_lignes = liste_lignes + liste_fichiers[0][1:]

for liste in liste_fichiers[1:]:

    print(liste[1])

    liste_lignes = liste_lignes + liste[1:]

crop = slice_size*cropLength+1

liste_lignes = liste_lignes[:crop]

print("Writing final file")

with open("Vect_Complet.csv", "w") as fichier_final:

    for ligne in liste_lignes:

        fichier_final.write(ligne)

print("Done!")

**SD4.2. Python code used to create the final dataset describing the microanatomy of a bone,** with ROI position, BVF, degree of anisotropy, direction of anisotropy vector, and angle of anisotropy vector with the three anatomical directions.

# -*- coding: utf-8 -*-

#Author : Cyril Etienne

#This script combines the cartographies of bone volume fraction (BVF) and anisotropy created in the

#dragonfly software into a microanatomy .csv dataset, listing all regions of interest (ROI), their

#position, their BVF, their degree of anisotropy, the direction of the anisotropy vector and the

#angle of the anisotropy vector with the proximodistal, mediolateral and craniocaudal axes of the bone.

#It takes as input two .tiff image stacks, one for BVF and one for anisotropy, exported from dragonfly

#and giving the value of BVF and anisotropy in each ROI. The two cartographies must have been done in

#the exact same computation box with the same radius and spacing.

#It also takes as input a vector .csv file, exported from dragonfly. If the anisotropy calculation was

#split into several computation boxes to save RAM, then the vector files must be recombined using the

#previous script first.

#all ROIs outside of bone space must be set at a negative value of BVF in dragonfly before exporting.

#This is because all ROIs outside bone space are removed, but ROIs inside bone space that might have

#BVF at 0 (meduallry cavity) are kept

#It is recommended that the script is run in three steps, stopping at each point marked "STOP", to

#check that everythicg is running correctly.

#Several steps require user input before running the script, and are marked with "User input"

import numpy as np

import os

import pandas as pd

from PIL import Image

os.chdir('D:/PATH/') #Path were the folders containing the image stacks can be found. Will also be the output folder

fichier = "INDIVIDUAL_microanat.csv" #Final file name

def import_heatmap(path):

    #Imports the BVF and anisotropy image stacks

    liste_arrays = []

    for file in os.listdir(path):

        if file[-5:] == ".tiff" or file[-4:] == ".tif": #checking if .tiff

            im = Image.open(path + "/" + file)

            liste_arrays.append(np.array(im))

    liste_lists = [array.tolist() for array in liste_arrays]

    liste_finale = []

    for liste in liste_lists:

        liste_finale.append([item for sublist in liste for item in sublist])

    liste_finale = [item for sublist in liste_finale for item in sublist]

    return(liste_finale)

def remove_zeros(cible, base):

    #Removes all ROIs with specified value at 0

    liste_indexs = []

    for i in range(len(base)):

        if base[i] != 0:

            liste_indexs.append(i)

    print("Index list finished. Indexes different from 0 : " + str(len(liste_indexs)))

    cible_finale = []

    for i in liste_indexs:

        cible_finale.append(cible[i])

    return cible_finale

def remove_below_zero(cible, base):

    #Removes all ROIs with specified value below 0

    liste_indexs = []

    for i in range(len(base)):

        if base[i] >= 0:

            liste_indexs.append(i)

    print("Index list finished. Indexes above or equal to 0 : " + str(len(liste_indexs)))

    cible_finale = []

    for i in liste_indexs:

        cible_finale.append(cible[i])

    return cible_finale

#USER INPUT

print("Importing cartographies")

aniso = import_heatmap('aniso') #folder with anisotropy image stack

compac = import_heatmap('compac') #folder with BVF image stack

chemin_Vecteurs = ("Vect_Complet.csv") #Vector file

print("Removing ROIs outside bone space)")

aniso2 = remove_below_zero(aniso, compac)

compac2 = remove_below_zero(compac, compac)

print("Importing vectors")

import matplotlib.pyplot as plt

vecteurs = np.genfromtxt(chemin_Vecteurs, delimiter=',', skip_header = 1)

aniso_vect = (vecteurs[:,4]**2+vecteurs[:,5]**2+vecteurs[:,6]**2)**0.5

aniso_vect = aniso_vect.tolist()

aniso_vect2 = remove_below_zero(aniso_vect, compac)

print("Checking anisotropy correspondance (manual)")

plt.hist2d(aniso_vect2,aniso2, bins = 50)

plt.hist2d(remove_zeros(aniso2, aniso2), remove_zeros(aniso_vect2, aniso2), bins = 50)

#STOP 1 : Checking that anisotropy as in the cartography and anisotropy recalculated from vector components are equal (manual)

#This ensures that the vectors and anisotropy datasets are correctly aligned

#The output graph must be a perfect diagonal. Another check is performed afterwards

vectDirX = remove_below_zero(vecteurs[:,4].tolist(), compac)

vectDirY = remove_below_zero(vecteurs[:,5].tolist(), compac)

vectDirZ = remove_below_zero(vecteurs[:,6].tolist(), compac)

vectPosX = remove_below_zero(vecteurs[:,1].tolist(), compac)

vectPosY = remove_below_zero(vecteurs[:,2].tolist(), compac)

vectPosZ = remove_below_zero(vecteurs[:,3].tolist(), compac)

print("Creating Dataframe")

DF_complet = pd.DataFrame()

DF_complet["Compacite"] = compac2

DF_complet["Anisotropie"] = aniso2

DF_complet["AnisotropieVECT"] = aniso_vect2

DF_complet["vectPosX"] = vectPosX

DF_complet["vectPosY"] = vectPosY

DF_complet["vectPosZ"] = vectPosZ

DF_complet["vectDirX"] = vectDirX

DF_complet["vectDirY"] = vectDirY

DF_complet["vectDirZ"] = vectDirZ

print("Chechinkg anisotropy corredpondance (automatic)")

#Checking that anisotropy as in the cartography and anisotropy recalculated from vector components match. Both are rounded to 0.001

DF_complet["Egalité"] = round(DF_complet["Anisotropie"], 3) == round(DF_complet["AnisotropieVECT"], 3)

if sum(DF_complet["Egalité"]) == len(aniso2):

    print("Anisotropy matches")

    DF_complet = DF_complet.drop('AnisotropieVECT', 1)

    DF_complet = DF_complet.drop('Egalité', 1)

else:

    print("Anisotropy does not match. Error number : " + str(len(aniso2)-sum(DF_complet["Egalité"])) + " / " + str(len(aniso2)))

    print("Due to rounding errors, some errors are possible without affecting the data. If their proportion of total number of ROIs is low, they can be ignored")

    print("A data set (DF_errors) with all the erroneous ROIs will be created. It can be checked manually.")

    if input("Ignore errors ? (y/n) ") == "y":

        DF_errors = DF_complet[DF_complet["Egalité"] == False]

        DF_complet = DF_complet.drop('AnisotropieVECT', 1)

        DF_complet = DF_complet.drop('Egalité', 1)

    else:

        DF_errors = DF_complet[DF_complet["Egalité"] == False]

        raise ValueError('Anisotropy does not match between the heatmap and the vector file.')

#STOP 2 : Checking anisotropy correspondance (automatic)

print("Creating ROI indexes")

VectPosX_entier = (vecteurs[:,1]-vecteurs[0,1])/(vecteurs[0,1]-vecteurs[1,1])

VectPosX_entier = VectPosX_entier.tolist()

VectPosX_entier = [abs(round(i)) for i in VectPosX_entier]

VectPosY_entier = (vecteurs[:,2]-vecteurs[0,2])/(vecteurs[0,2]-vecteurs[max(VectPosX_entier)+3,2])

VectPosY_entier = VectPosY_entier.tolist()

VectPosY_entier = [abs(round(i)) for i in VectPosY_entier]

VectPosZ_entier = (vecteurs[:,3]-vecteurs[0,3])/(vecteurs[0,3]-vecteurs[(max(VectPosX_entier)+1)*(max(VectPosY_entier)+1),3])

VectPosZ_entier = VectPosZ_entier.tolist()

VectPosZ_entier = [abs(round(i)) for i in VectPosZ_entier]

vectPosX_entier2 = remove_below_zero(VectPosX_entier, compac)

vectPosY_entier2 = remove_below_zero(VectPosY_entier, compac)

vectPosZ_entier2 = remove_below_zero(VectPosZ_entier, compac)

DF_complet["vectIndexX"] = vectPosX_entier2

DF_complet["vectIndexY"] = vectPosY_entier2

DF_complet["vectIndexZ"] = vectPosZ_entier2

print("Computing angles between anisotropy vectors and anatomical orientations")

def angle_between(v1, v2):

    """ Returns the angle in radians between vectors 'v1' and 'v2'::

            >>> angle_between((1, 0, 0), (0, 1, 0))

            1.5707963267948966

            >>> angle_between((1, 0, 0), (1, 0, 0))

            0.0

            >>> angle_between((1, 0, 0), (-1, 0, 0))

            3.141592653589793

    """

    v1_u = v1 / np.linalg.norm(v1)

    v2_u = v2 / np.linalg.norm(v2)

    return np.arccos(np.clip(np.dot(v1_u, v2_u), -1.0, 1.0))

angleX = []

angleY = []

angleZ = []

for i in range(len(vectDirX)):

    vecteur = (vectDirX[i],vectDirY[i],vectDirZ[i])

    #print(i)

    angleZ.append(round(angle_between((0,0,1),vecteur)*57.2958,2))

    angleX.append(round(angle_between((1,0,0),vecteur)*57.2958,2))

    angleY.append(round(angle_between((0,1,0),vecteur)*57.2958,2))

DF_complet["angleX"] = angleX

DF_complet["angleY"] = angleY

DF_complet["angleZ"] = angleZ

print("Writing final file")

DF_complet.to_csv(fichier)

print("Done!")

**SD4.3. R code used to visualise the ROIs.**

#Author : Cyril Etienne

#This script takes the microanatomy file assembled previously and uses it to display trabecular anisotropy (or BVF) in 3D.

library(rgl)

setwd("C:/PATH TO FILES/")

data = read.csv("CS8029_tibia_microanat.csv") #Microanatomy file from python script

model <- vcgPlyRead("CS8029_tibia_modele.ply") #3D Model exported from dragonfly. It is recommended to decimate it below 25,000 faces

#Colour functions------------------------------------------

viridis_scale = function(c){

  r = (-11.5444*(c**5)+24.7079*(c**4)-14.4479*(c**3)+2.122875*(c**2)-0.13446*c+0.279996078)

  g = (-1.21921*(c**4)+2.267498*(c**3)-1.73326*(c**2)+1.582772*c+0.001702159)

  b = (26.2720929*(c**6)-65.3209*(c**5)+56.65624*(c**4)-19.2918*(c**3)+0.091963*(c**2)+1.386773*c+0.332663775)

  return(rgb(r,g,b))

}

plasma_scale = function(c){

  r = (-305.8*(c**2)+525.8*c+15.89)/256

  g = c

  b = (638.2*(c**3) - 1112.01*(c**2) + 374.15*c+131.1)/256

  return(rgb(r,g,b))

}

#ANISOTROPY---------------------------------

data_2 = data[data$Compacite < 0.85,] #Keeping only regions below 0.85 BVF, considered trabecular

quant = quantile(data_2$Anisotropie, 0.75); quant #Extracting the upper quartile of anisotropy in the dataset

data_2 = data_2[data_2$Anisotropie > quant,]

bin = 2 #One out of every two regions is removed, in each dimension

data_3 = data_2[data_2$vectIndexX %% bin == 0,];data_3 = data_3[data_3$vectIndexY %% bin == 0,];data_3 = data_3[data_3$vectIndexZ %% bin == 0,]

#OPTIONAL : these can be used to further filter the data

data_3 = data_3[data_3$vectPosX > 60,] #Filter according to position on X axis

data_3 = data_3[data_3$Compacite < 0.5,] #Filter according to BVF

data_3 = data_3[data_3$Anisotropie > 0.2,] #Filter according to anisotropy

#COLOURING

#According to anisotropy

teinte = (data_3$Anisotropie-min(data_3$Anisotropie))/(max(data_3$Anisotropie)-min(data_3$Anisotropie)) #Scale between min and max in dataset

couleur = viridis_scale(teinte)

#According to BVF

teinte = data_3$Compacite/0.85 #Scale between 0 and 0.85

couleur = plasma_scale(teinte)

#According to 3D direction

couleur = rgb(abs(data_3$vectDirX)/max(data_3$Anisotropie), abs(data_3$vectDirY)/max(data_3$Anisotropie),

              abs(data_3$vectDirZ)/max(data_3$Anisotropie))

#Doubling the colour vector, as segments need a colour for both points.

couleur_final = c()

for (col in couleur){

  couleur_final = c(couleur_final, rep(col,2))

}

magnif = 10 #magnification of vector length

#Displaying

open3d(windowRect=c(100,100,1500,750))

segments3d(rbind(data_3$vectPosX, data_3$vectPosX + data_3$vectDirX*magnif)/1000,

           rbind(data_3$vectPosY, data_3$vectPosY + data_3$vectDirY*magnif)/1000,

           rbind(data_3$vectPosZ, data_3$vectPosZ + data_3$vectDirZ*magnif)/1000,

           color = couleur_final, lwd = 2)

shade3d(model, col = "ivory", alpha = 0.3, addp = TRUE)

rgl.viewpoint(0, 0, fov = 0)
