# Supplementary material for: How do forelimb long bones adapt in rhinoceroses? An in‐depth examination of their microanatomy
Source: J Anat. 2026 Jun 1:10.1111/joa.70180. Online ahead of print. doi: 10.1111/joa.70180 (PMC13398847; doi:10.1111/joa.70180)
Supplement: Supplementary file 5 — Supplementary Data S5. Intraspecific variations. [file JOA-9999-0-s006.docx]

Supplementary data 5. Intraspecific variations

**SD 5.1. Qualitative comparisons**

2D sections enabled to estimate intraspecific variation. All individuals studied usually present coherent variations between their bones. That is, if an individual has a thicker cortex in its humerus compared with other individuals of its species, the other two bones usually present a thicker cortex as well. Overall, all species are also homogeneous in terms of bone size, with *D. sumatrensis* individuals having the smallest bones and *R. unicornis* the largest ones.

Most individuals of *C. simum* are similar, except for *C. simum* MNHN ZM-MO-2005-297, which is a clear outlier presenting a much thinner cortex for its bones (Fig. S5.1A-C). Regarding trabecular bone, its trabecular density is reduced as well compared to the others, with a greater spacing of the trabeculae, but its trabecular thickness appears similar. Its trabecular anisotropy, however, does not seem to differ from that of the other *C. simum* specimens. That specimen was likely affected by some kind of sickness, such as age-induced osteopenia. Mallet et al. (2019) studied that specimen’s outer bone shape and found it very similar to other *C. simum* individuals. The individual *C. simum* RBINS 1904 has a slightly higher apparent trabecular density than other specimens, in all its bones. It also has a tighter spongiosa than the others. That specimen is the only subadult in our *C. simum* sample. The other specimens (*C. simum* NHM 2018.143, *C. simum* RBINS 35028, *C. simum* NMB 8029) are extremely similar to one another.

Our four *D. bicornis* individuals are homogeneous in terms of cortical bone thickness, which is for all the bones much higher than in the other species (overall, only *C. simum* RBINS 1904 approaches the relative cortical thickness of the least dense *D. bicornis*). Their trabecular bone, however, presents significant intraspecific variation. One specimen, *D. bicornis* MNHN-ZM-AC-1936-644, is characterized by an extremely high trabecular density, particularly in its zeugopod bones, reminiscent of osteosclerosis (a pathological increase in bone density). In many parts (e.g. radial and ulnar diaphyses, olecranon; Fig. S5.1D-F), trabecular bone becomes so dense that it looks more like porous cortical bone. This makes trabecular anisotropy difficult to determine, although it seems to follow the same pattern as in other *D. bicornis* and rhinos in general. That specimen is a subadult female, that belonged to a circus upon its death. No information is known about its upbringing. The collection records of the Muséum national d’Histoire naturelle indicate that the specimen weighed 1800 kg when it entered the collection, far above the normal range of *D. bicornis* (800 – 1300 kg). Another specimen, *D. bicornis* RBINS 9714, also presents a very high trabecular density in its trabecular bone, although to a lesser extent, in the proximal radial diaphysis, distal humeral epiphysis and olecranon. That specimen is also a female. Those two, along with *D. sumatrensis* NMB 10529 are the only specimens for which we are certain that they are female, although eight specimens are of unknown sex. Another of our *D. bicornis* specimens (*D. bicornis* NMB 10594) came from a circus, but presents a more typical microanatomy. *D. bicornis* MNHN-ZM-AC-1936-644 and *D. bicornis* NMB 10594 are the only specimens in our sample coming from a circus, although four specimens are of unknown origin. All *D. bicornis* specimens have at least a slightly higher apparent trabecular density than the other species.

Five of our six *R. unicornis* individuals are extremely similar in terms of cortical bone thickness. Spongiosa tightness is on average higher than in the other species, but is slightly lower in *R. unicornis* MNHN ZM-MO-1960-59 and in *R. unicornis* NMB 7351 than in the other *R. unicornis* specimens.

As for individuals of *R. sondaicus*, they again vary little in terms of cortical thickness. *R. sondaicus* NMB 10885 has a slightly thinner cortical bone in all its bone. All three individuals are clearly subadults. Trabecular bone structure is similar in all three individuals. *R. sondaicus* overall shares a very similar microanatomy with *R. unicornis*.

Individuals of *D. sumatrensis* vary little in terms of cortical bone thickness. *D. sumatrensis* RBINS 1204 has the lowest relative cortical thickness of our *D. sumatrensis*, being the only one that visibly differs from the others, although the difference remains small. That specimen is the only one to present a distinct medullary cavity in all of its bones, albeit very small in the radius and ulna. Small, very sparse trabeculae may still be seen in these cavities; the one in the humerus is also divided by several sets of highly anisotropic trabeculae that go from the caudal to the cranial side of the bone. *D. sumatrensis* MNHN ZM-AC-1903-300 presents a distinct medullary cavity in its humerus, all other *D. sumatrensis* individuals have their medullary cavities completely filled by spongiosa, as do all other rhinos. *D. sumatrensis* NHM ZD-1948-12-20-1 is most peculiar in presenting, in its trabecular bone, small clumps of bone that are markedly thicker than surrounding trabeculae (Fig. S5.1H). These are generally between 0.5 and 1 mm in size, and are scattered all over the trabecular bone.


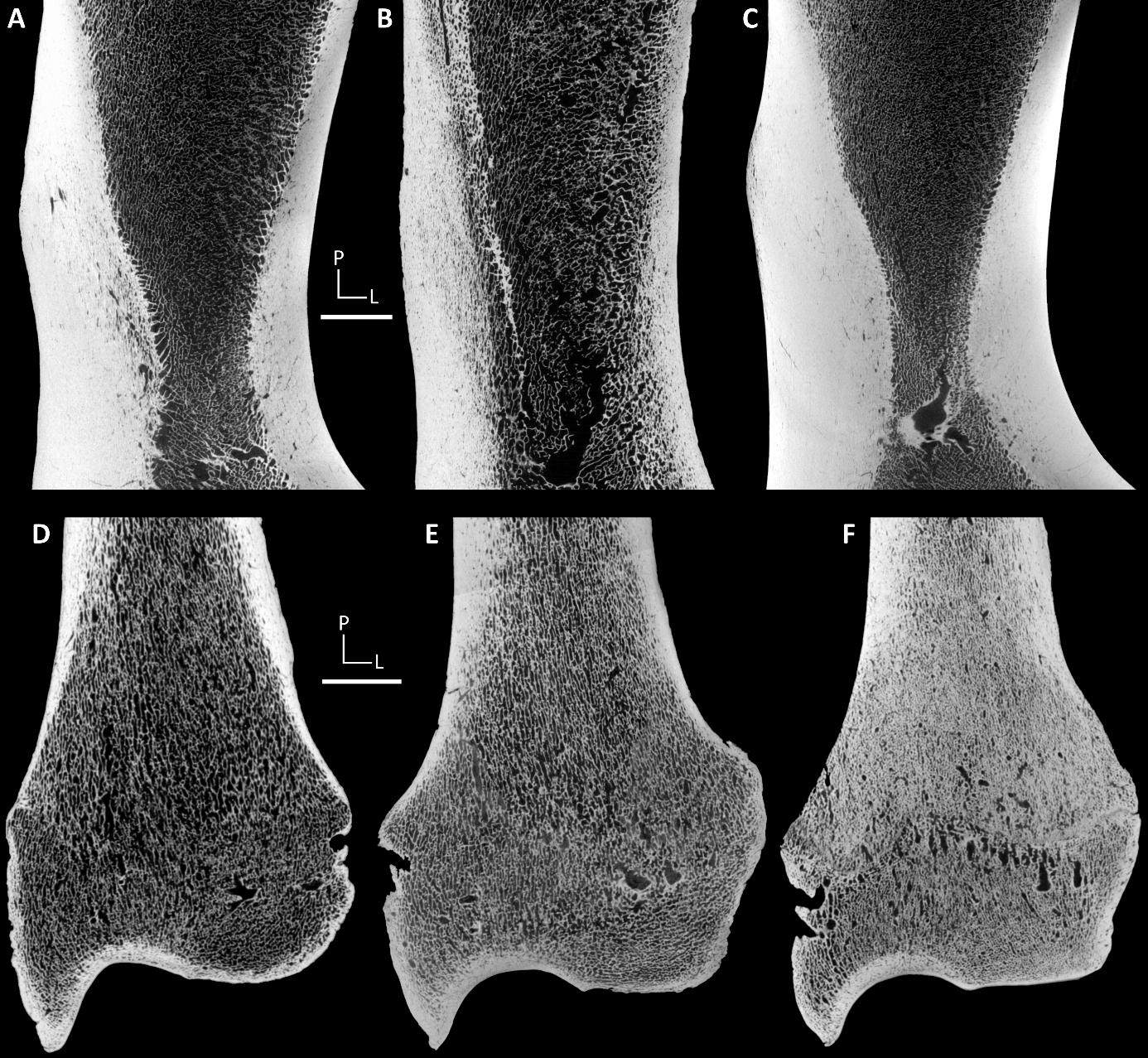


**Fig. S5.1. Intraspecific comparisons of selected features. A-C**: coronal slices in the middle of the diaphysis of the humerus of C. simum NMB 8029 (A), C. simum MNHN ZM-MO-2005-297 (B) and C. simum RBINS 1904 (C). **D-F**: coronal slices in the distal epiphysis of the radius of D. bicornis MNHN ZM-AC-1944-278 (D), D. bicornis NMB 10594 (E) and D. bicornis MNHN ZM-AC-1936-644 (F). Scale bars are two centimetres. L: lateral, P: proximal.

**SD 5.2. Impact of age and upbringing**

Ten out of 23 individuals in our sample are subadults, as indicated by the partial fusion of their epiphyses. Unfortunately, those are not evenly distributed in our sample. All of our *R. sondaicus* and five out of six of our *R. unicornis* are subadults, whereas only one specimen of each remaining species is subadult. Most of those subadults however only present a faint remnant of an epiphyseal line that was not even visible on the surface of the bone, indicating them as late subadults. It is likely our *R. unicornis* were had not reached their full size yet, but they still present longer bones than all other specimens. This makes it difficult to detangle differences due to age and differences due to species. One of such differences is that subadult specimens seem to present a tighter spongiosa, with a greater trabecular number than the adults and less clear patterns of anisotropy, although they present some variation in this regard. This is coherent with a decrease in trabecular number with age observed in other species such as horses and mice, although such a relation is not clear for trabecular number in humans (Ding et al., 2002; Glatt et al., 2007; Fürst et al., 2008; Kivell, 2016; McGivern et al., 2020). This could be due to reductions in bone volume fraction during the life of our individuals, which seems to be done through trabecular number reduction (Saparin et al., 2011). It would be ideal, of course, to precisely know the age of the specimens and to quantify parameters such as trabecular number, trabecular thickness and bone volume fraction to provide a more definite answer.

The individual *C. simum* MNHN ZM-MO-2005-297 was likely affected by some kind of sickness, resulting in a greatly reduced cortical thickness and an increase in trabecular spacing. Mallet et al. (2019) studied that specimen, and did not find morphological differences between it and other specimens (see neighbour-joining trees in supplemental information in Mallet et al., 2019). It is interesting that anisotropy does not seem to be affected, with the location and direction of anisotropic trabeculae remaining mostly the same. This is consistent with what is observed in the femur of elderly humans (Whitmarsh et al., 2019), where cortical thickness and trabecular bone mineral density decrease with age, but not the degree of anisotropy. Our specimen may have been elderly, suffering from osteopenia (a loss in bone density), or both. This illustrates the great plasticity of microanatomy compared with outer shape.

Overall, of our six specimens for which we are certain they died in captivity (two in circuses, four in zoos), three present highly visible alterations in their microanatomy: severe osteopenia in *C. simum* MNHN ZM-MO-2005-297 (Fig. S5.1A-C), osteosclerosis in *D. bicornis* MNHN ZM-AC-1936-644 (Fig. S5.1D-F), and mild osteopenia in the hindlimb of *R. unicornis* MNHN ZM-AC-1960-59. Only the later also presented outer shape alterations, in its femur only (Mallet et al., 2019). Rhinoceroses are known to suffer pathologies when kept in captivity, especially in their feet, which can result in both osteopenia and osteosclerosis (von Houwald and Flach, 1998; Galateanu et al., 2013; von Houwald, 2016). It is likely the differences we observe result from such pathologies. *D. bicornis* MNHN ZM-AC-1936-644, in particular, belonged to a circus; it is likely the substrate of its enclosure was too hard, and that its alimentation was inadequate, perhaps with too much hay replacing its natural browse. The collection records of the Muséum national d’Histoire naturelle indicate that the specimen weighed 1800 kg when it entered the collection, far above the normal range of *D. bicornis* (800 – 1300 kg). Another specimen belonged to a circus and did not present such alterations, but it may have been raised in an entirely different setting and acquired by the circus later in life. It and the two others captive bred specimens do not differ from wild-bred specimens, and in particular do not present the decrease in BVF observed in, e.g., felid long bones and armadillo vertebrae (Chirchir et al., 2022; Zack et al., 2022). Studies on trabecular BVF and captivity are still scarce, but Cottereau et al. (2022) did not find significant differences in the microanatomy of the calcaneus of boars raised in captivity or in the wild, either. Perhaps their great body weight maintains high forces and thus high BVF in the limbs of rhinoceroses, regardless of their activity levels, leading to few microanatomical differences when pathologies are not present.

### **References**

**Chirchir, H., Ruff, C., Helgen, K.M., Potts, R., 2022.** Effects of reduced mobility on trabecular bone density in captive big cats. *Royal Society Open Science* **9**, 211345. https://doi.org/10.1098/rsos.211345

**Cottereau, R., Ortiz, K., Locatelli, Y., Houssaye, A., Cucchi, T., 2022.** Can growth in captivity alter the calcaneal microanatomy of a wild ungulate? bioRxiv, 504790, ver. 5 peer-reviewed and recommended by *Peer Community in Archaeology*.

**Ding, M., Odgaard, A., Linde, F., Hvid, I., 2002.** Age-related variations in the microstructure of human tibial cancellous bone. *Journal of Orthopaedic Research* **20**, 615–621. https://doi.org/10.1016/S0736-0266(01)00132-2

**Fürst, A., Meier, D., Michel, S., Schmidlin, A., Held, L., Laib, A., 2008.** Effect of age on bone mineral density and micro architecture in the radius and tibia of horses: An Xtreme computed tomographic study. *BMC Vet Res* **4**, 3. https://doi.org/10.1186/1746-6148-4-3

**Galateanu, G., Hildebrandt, T.B., Maillot, A., Etienne, P., Potier, R., Mulot, B., Saragusty, J., Hermes, R., 2013.** One Small Step for Rhinos, One Giant Leap for Wildlife Management- Imaging Diagnosis of Bone Pathology in Distal Limb. *PLOS ONE* **8**, e68493. https://doi.org/10.1371/journal.pone.0068493

**Glatt, V., Canalis, E., Stadmeyer, L., Bouxsein, M.L., 2007.** Age-Related Changes in Trabecular Architecture Differ in Female and Male C57BL/6J Mice. *Journal of Bone and Mineral Research* **22**, 1197–1207. https://doi.org/10.1359/jbmr.070507

**Kivell, T.L., 2016.** A review of trabecular bone functional adaptation: what have we learned from trabecular analyses in extant hominoids and what can we apply to fossils? *Journal of Anatomy* **228**, 569–594. https://doi.org/10.1111/joa.12446

**Mallet, C., Cornette, R., Billet, G., Houssaye, A., 2019.** Interspecific variation in the limb long bones among modern rhinoceroses—extent and drivers. *PeerJ* **7**, e7647. https://doi.org/10.7717/peerj.7647

**McGivern, H., Greenwood, C., Márquez-Grant, N., Kranioti, E.F., Xhemali, B., Zioupos, P., 2020.** Age-Related Trends in the Trabecular Micro-Architecture of the Medial Clavicle: Is It of Use in Forensic Science? *Frontiers in Bioengineering and Biotechnology* 7.

**von Houwald, F., 2016.** Causes and prevention of foot problems in Greater one-horned rhinoceros *Rhinoceros unicornis* in zoological institutions. *International Zoo Yearbook* **50**, 215–224. https://doi.org/10.1111/izy.12116

**von Houwald, F., Flach, E.J., 1998.** Prevalence of chronic foot disease in captive greater one-horned rhinoceros (*Rhinoceros unicornis*). *European Association of Zoo- and Wildlife Veterinarians* Second Scientific Meeting 323–327.

**Whitmarsh, T., Otake, Y., Uemura, K., Takao, M., Sugano, N., Sato, Y., 2019.** A cross-sectional study on the age-related cortical and trabecular bone changes at the femoral head in elderly female hip fracture patients. *Sci Rep* **9**, 305. https://doi.org/10.1038/s41598-018-36299-y

**Zack, E.H., Smith, S.M., Angielczyk, K.D., 2022.** Effect of captivity on the vertebral bone microstructure of xenarthran mammals. *The Anatomical Record* **305**, 1611–1628. https://doi.org/10.1002/ar.24817
