# Supplementary material for: How do forelimb long bones adapt in rhinoceroses? An in‐depth examination of their microanatomy
Source: J Anat. 2026 Jun 1:10.1111/joa.70180. Online ahead of print. doi: 10.1111/joa.70180 (PMC13398847; doi:10.1111/joa.70180)
Supplement: Supplementary file 6 — Supplementary Data S6 – Interspecific variations of quantitative parameters. [file JOA-9999-0-s001.docx]

Supplementary data 6 – Interspecific variations of quantitative parameters


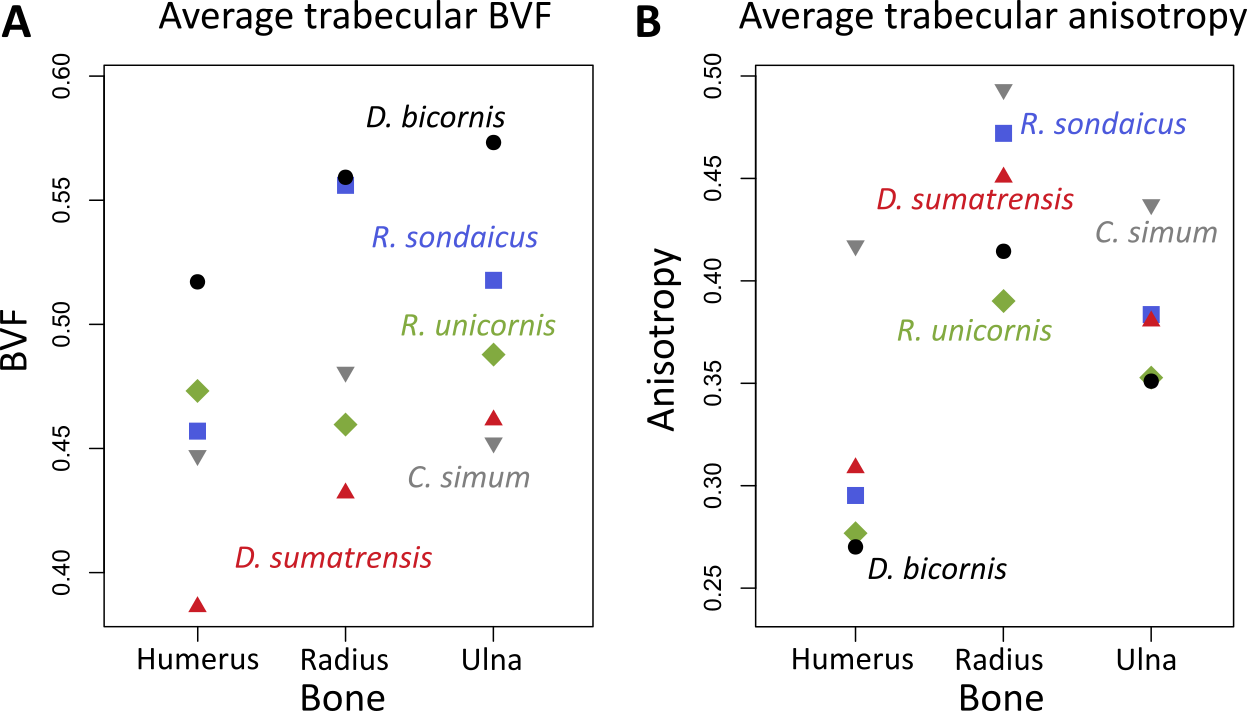


Fig S6.1. Average trabecular bone volume fraction and anisotropy across all bones studied quantitatively.


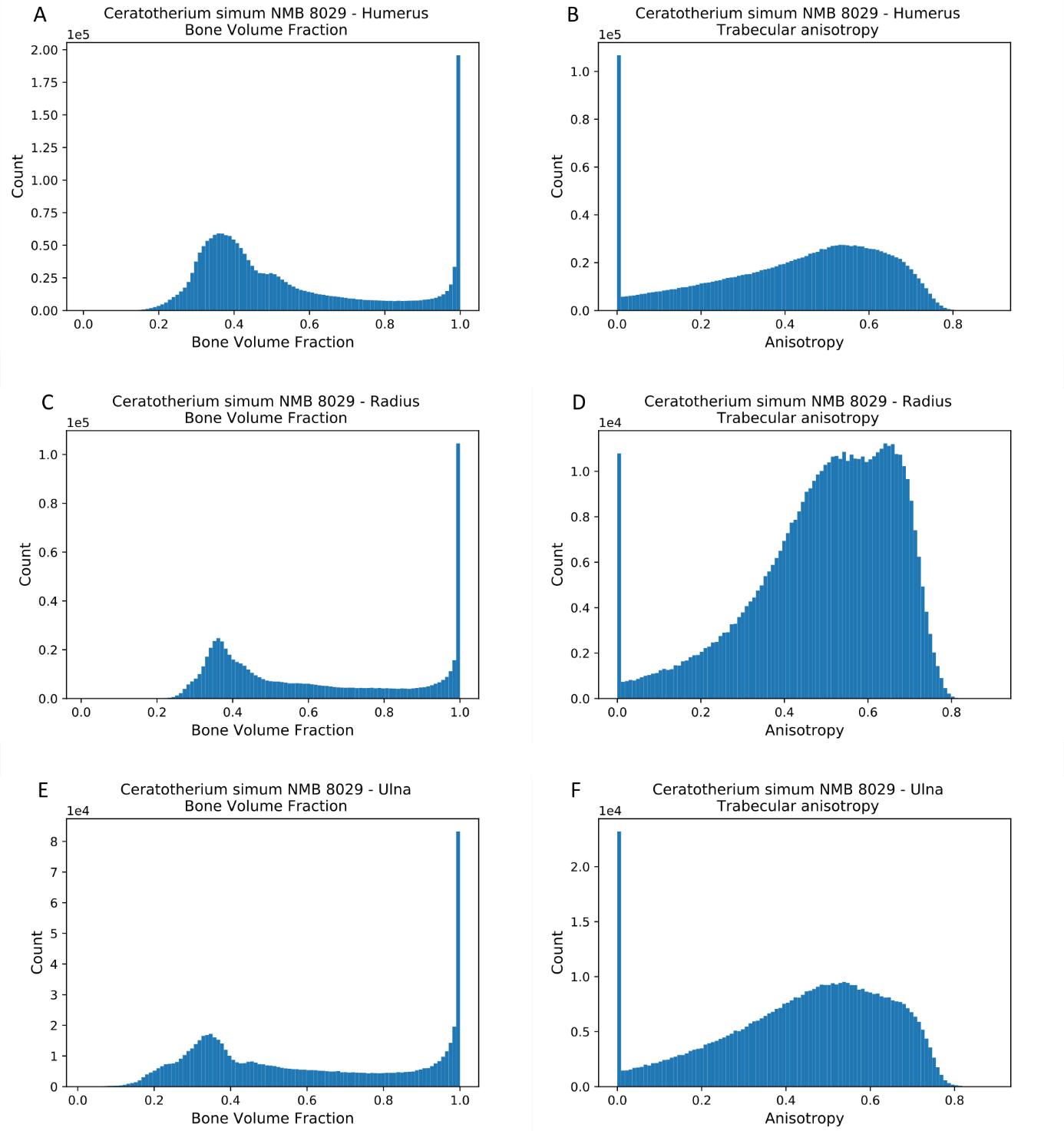


Fig S6.2. Bone volume fraction (BVF; A, C, E) and anisotropy (B, D, F) distribution in the three forelimb long bones of our studied *C. simum* individual (A, B: humerus, C, D: radius, E, F: ulna). Anisotropy considers only trabecular bone regions of interest, with a BVF value below 0.85.


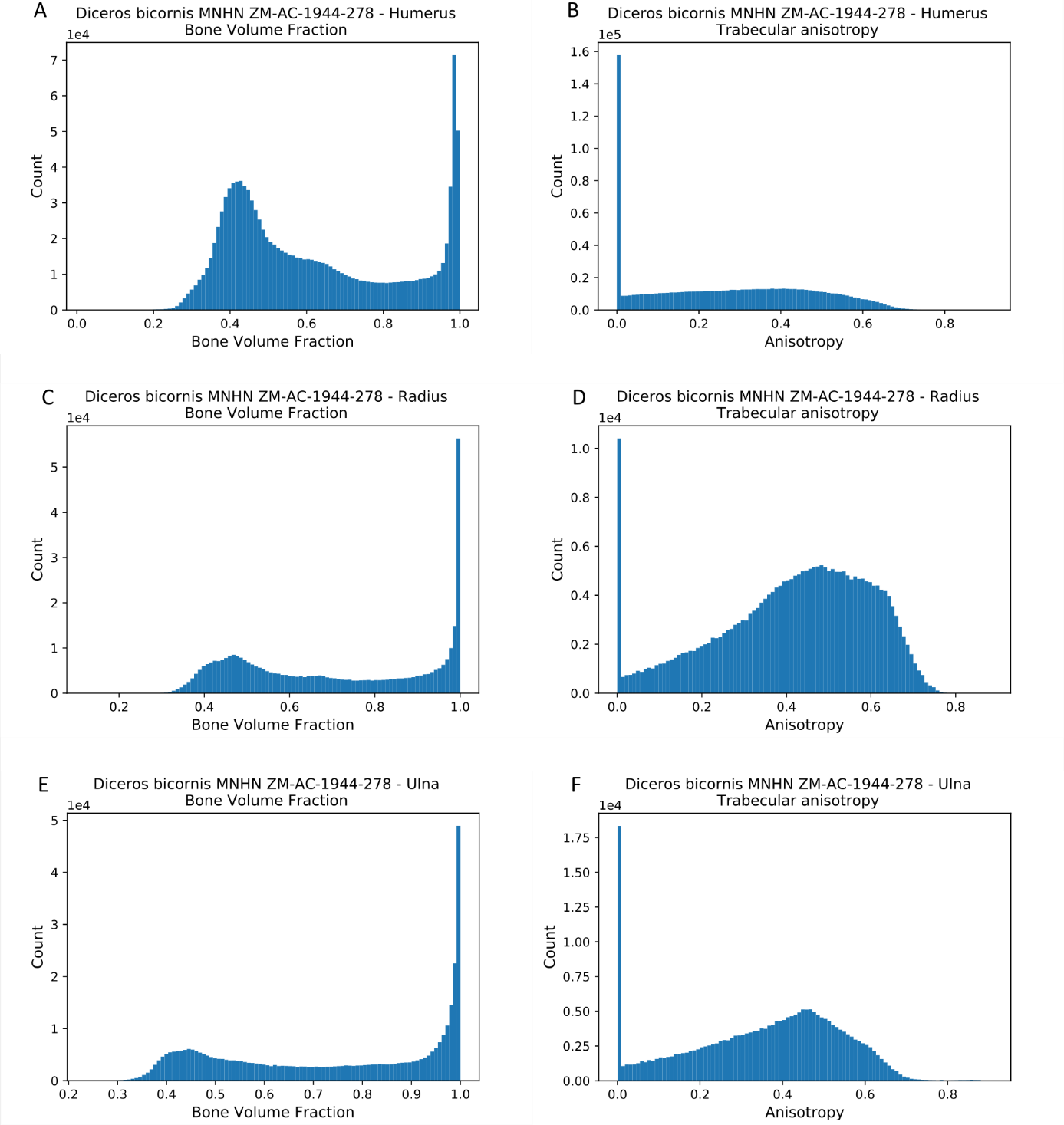


Fig S6.3. Bone volume fraction (BVF; A, C, E) and anisotropy (B, D, F) distribution in the three forelimb long bones of our studied *D. bicornis* individual (A, B: humerus, C, D: radius, E, F: ulna). Anisotropy considers only trabecular bone regions of interest, with a BVF value below 0.85.


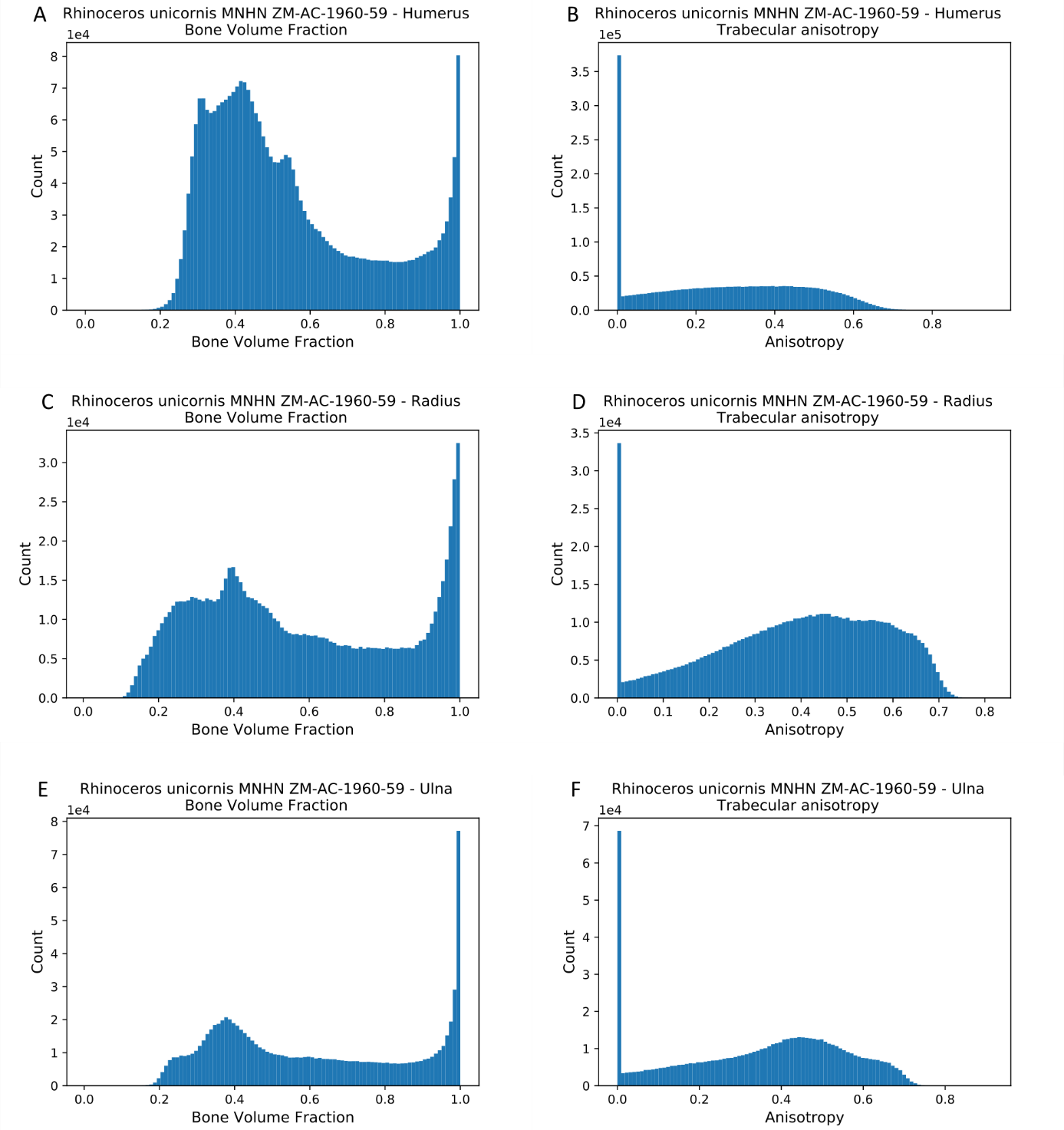


Fig S6.4. Bone volume fraction (BVF; A, C, E) and anisotropy (B, D, F) distribution in the three forelimb long bones of our studied *R. unicornis* individual (A, B: humerus, C, D: radius, E, F: ulna). Anisotropy considers only trabecular bone regions of interest, with a BVF value below 0.85.


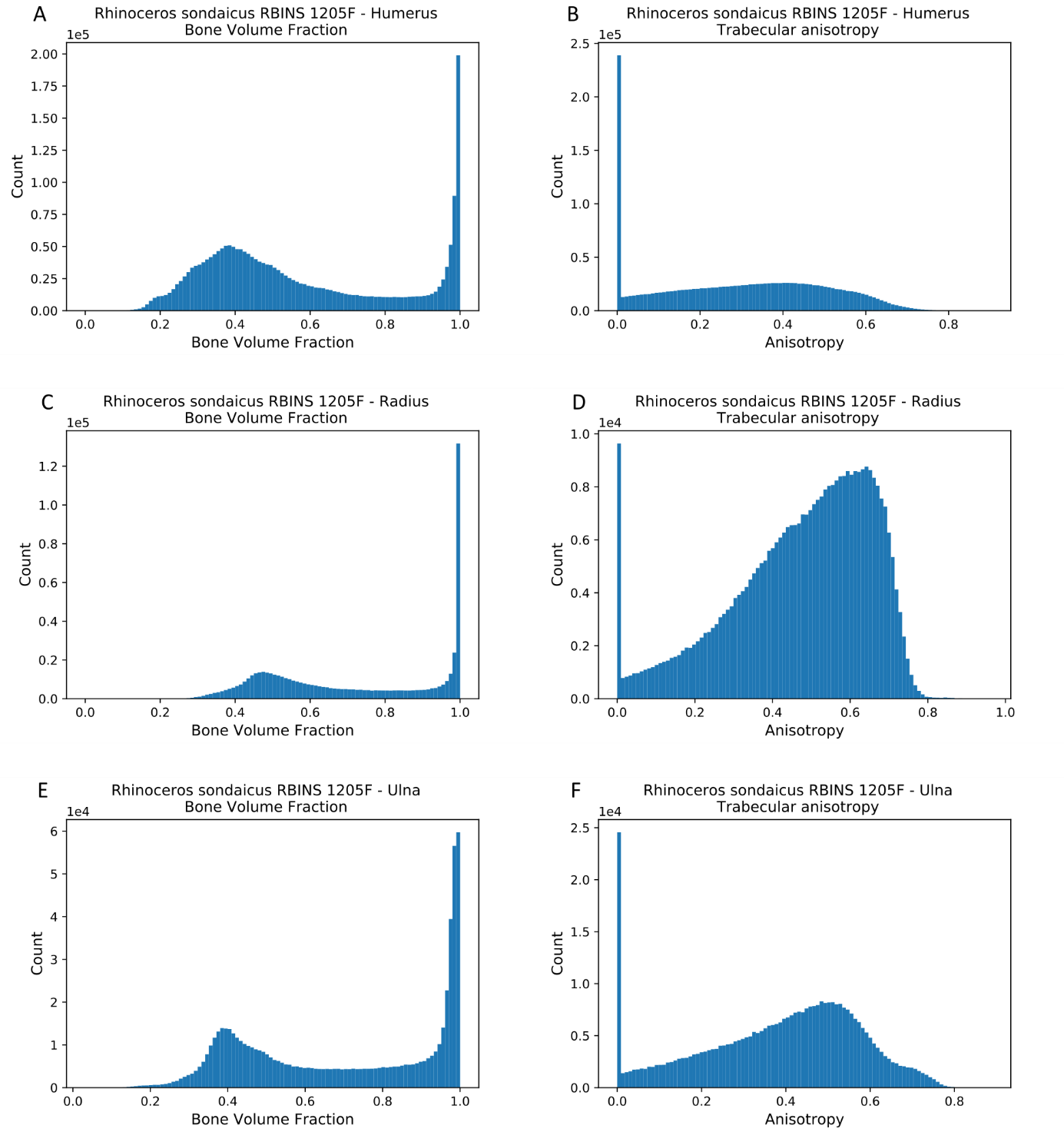


Fig S6.5. Bone volume fraction (BVF; A, C, E) and anisotropy (B, D, F) distribution in the three forelimb long bones of our studied *R. sondaicus* individual (A, B: humerus, C, D: radius, E, F: ulna). Anisotropy considers only trabecular bone regions of interest, with a BVF value below 0.85.


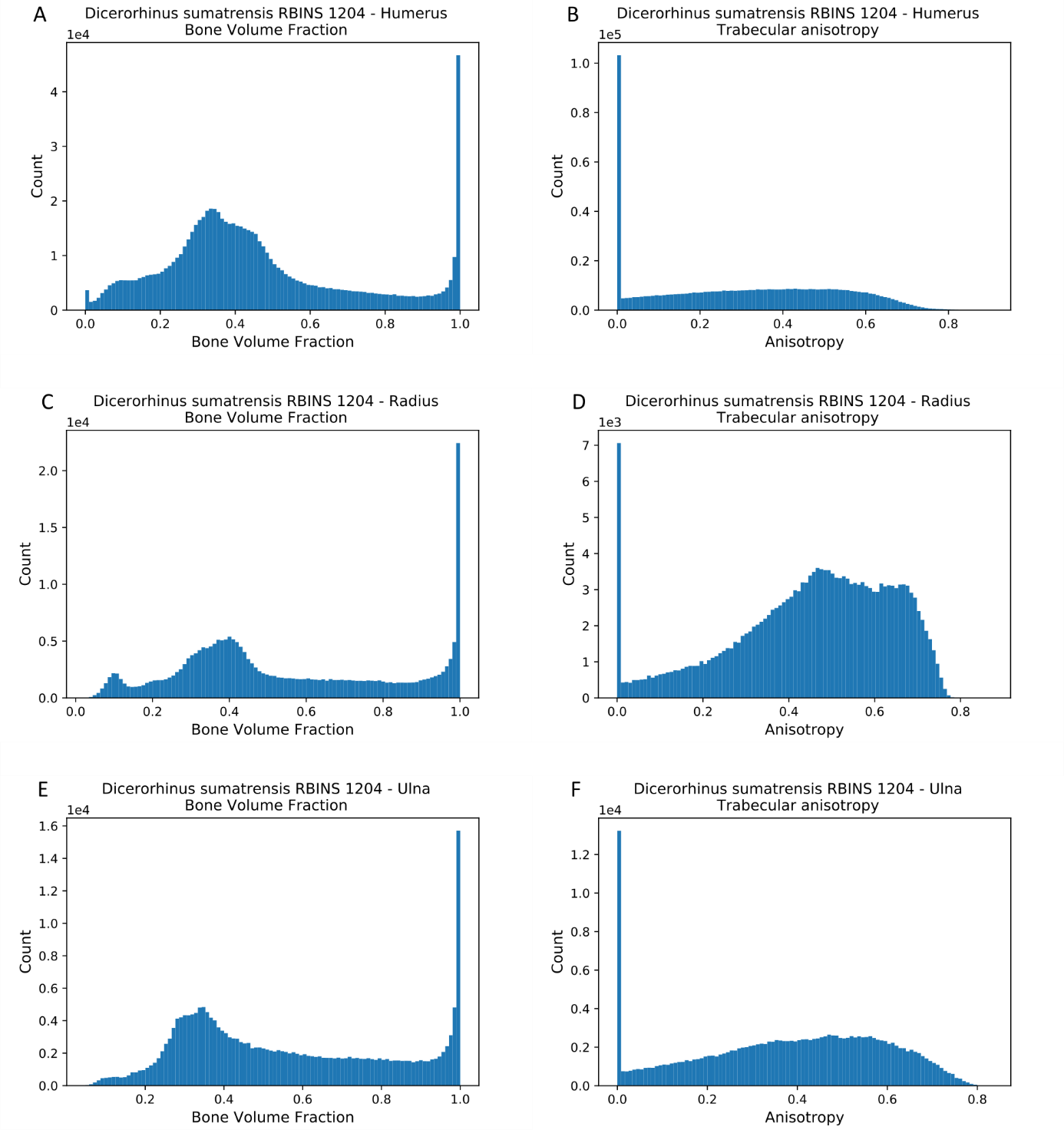


Fig S6.6. Bone volume fraction (BVF; A, C, E) and anisotropy (B, D, F) distribution in the three forelimb long bones of our studied *D. sumatrensis* individual (A, B: humerus, C, D: radius, E, F: ulna). Anisotropy considers only trabecular bone regions of interest, with a BVF value below 0.85.

## **Interspecific variations of quantitative parameters**

*Diceros bicornis* presents overall a higher average trabecular BVF for all bones, while *Dicerorhinus sumatrensis* has the lowest average trabecular density, with the exception of the ulna, for which *C. simum* has the lowest average (Fig S6.1). Averages give a simple picture of the trabecular BVF of each bone, but it may be a too simple metric, as it tells us nothing about the distribution of the values. Comparing the distribution of BVF in each individual bone is more informative (Figs. S6.2-6). The general case, for each bone, is to observe two main peaks for BVF: one around 1, corresponding to ROIs inside cortical bone, and one between ~0.3-0.5, corresponding to the most frequent BVF for trabecular bone. The trabecular peak of the histogram usually rises sharply around ~0.2 to 0.4, in a pattern similar to a gaussian curve. It then lowers more progressively, presenting more regions between 0.5-0.8 BVF than expected if the distribution kept following a gaussian curve. Those regions of trabecular bone with relatively high BVF could correspond to regions subject to higher forces that require more bone to spread the forces on, whereas regions close to the mode of the gaussian-like left side of the histogram probably correspond to a baseline state of trabecular BVF in rhinos.

As expected, most rhinos have no ROIs with BVF below 0.15, indicating that all ROIs present at least 15% of bone tissue, meaning their medullary cavities are entirely filled with trabecular bone (Fig S6.2-6). *D. sumatrensis*’ bones are exception, being characterised by much more frequent values of BVF under 0.15 (Fig. S6.6). The humerus even has a small peak at 0 BVF, indicating a medullary cavity. Those values under 0.15 are usually not in continuity with the trabecular peak, but seem to form a peak of their own. This perhaps indicates regions subject to a much lower stress, below a certain threshold that would mean almost all the trabeculae in this region will be resorbed (as they are in most mammals that present a medullary cavity devoid of bone). This is consistent with the sections. Overall, considering qualitative comparisons of the sections, we could have expected D. bicornis to present even denser trabecular bone, but the specimen we chose seemed the least dense of the species. It still presents the densest BVF for the three bones. Conversely, neither C. simum nor R. unicornis have the densest trabecular bone, as could have been expected from their greater body mass than the others’, which is in line with qualitative comparisons from the sections.

The average of trabecular anisotropy shows more consistence than that of BVF (Fig. S6.1B). *C. simum*always has the highest anisotropy, followed, with more variations, by *R. sondaicus* and *D. sumatrensis*, then *D. bicornis*, and *R. unicornis*. Bones also present consistent patterns: the radius has the highest average BVF in all species, followed by ulna, and humerus. Regarding the distribution of anisotropy, all histograms are quite similar. The general case is again to have two peaks, one at 0 anisotropy and one around 0.5-0.6 (Figs. S6.2-6). The frequency of ROIs at 0 anisotropy is the lowest in the radius and the highest in the humerus, in line with the averages, indicating that the number of ROIs at 0 anisotropy is the main driver of the lower averages observed in the humerus than in the radius an ulna. Indeed, the values of the second peak of the histogram do not vary much between the different bones and species, except for C. simum which usually presents slightly higher values. The second peak does not follow a gaussian distribution; it usually falls more sharply on the right side. Regions at 0 anisotropy are likely not subject to intense directional loads, whereas regions close to the maximal value of anisotropy probably experience a great load in one particular direction, in accordance with the theory of trabecular bone adaptation.
