# Supplementary material for: How do forelimb long bones adapt in rhinoceroses? An in‐depth examination of their microanatomy
Source: J Anat. 2026 Jun 1:10.1111/joa.70180. Online ahead of print. doi: 10.1111/joa.70180 (PMC13398847; doi:10.1111/joa.70180)
Supplement: Supplementary file 7 — Supplementary Data S7. Microanatomy of the humerus in C. simum. [file JOA-9999-0-s002.docx]

Supplementary data 7. Microanatomy of the humerus in C. simum.


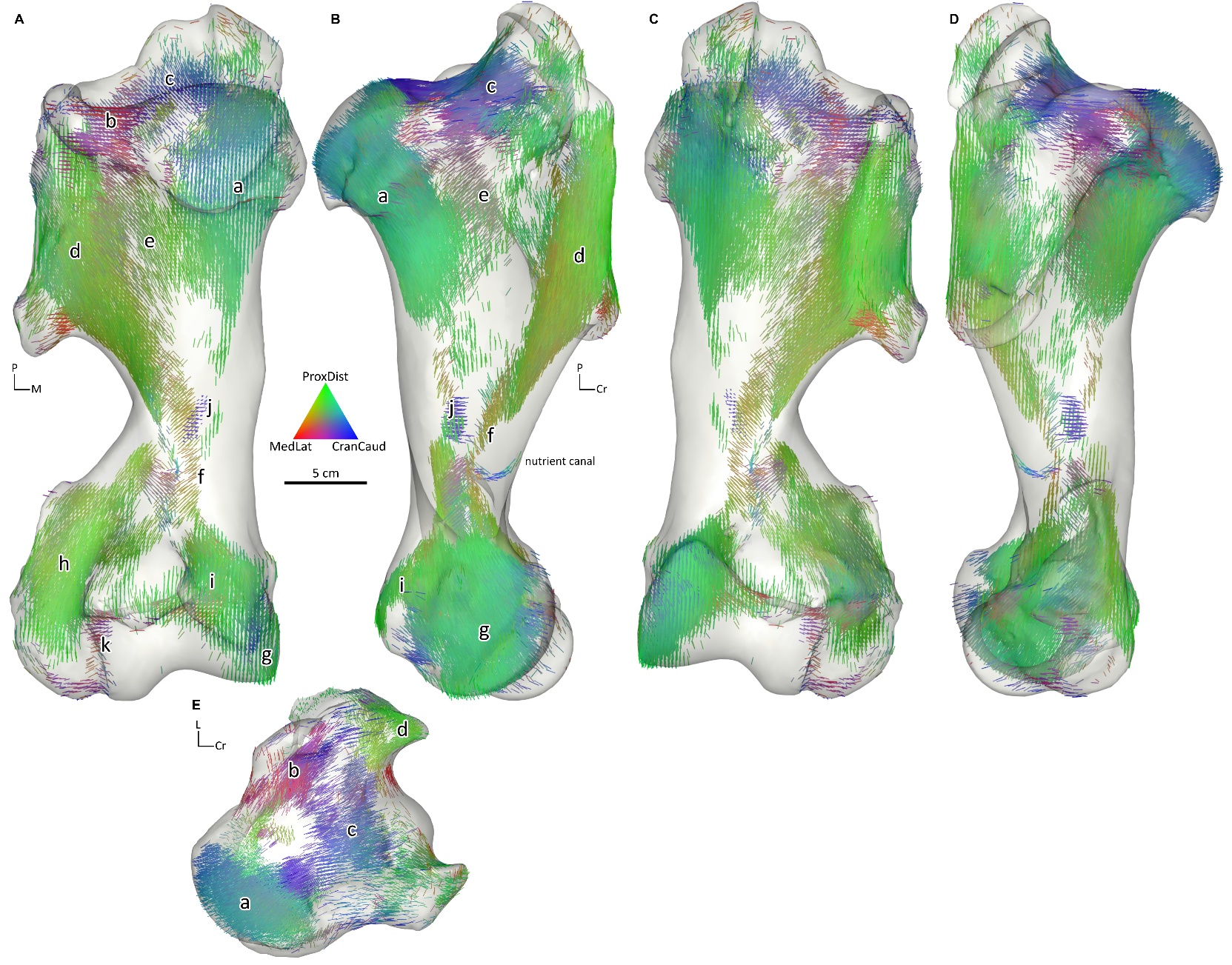


Fig. S7.1. **A.** 3D cartographies of the 25% most anisotropic trabecular ROIs, in caudal (**A**), medial (**B**), cranial (**C**), lateral (**D**), and proximal (**E**) views. ROIs are represented as vectors pointing in the direction of the anisotropy, and are additionally colour-coded according to their direction. Small-case letters indicate sets of anisotropic trabeculae described in the main text. P: proximal, M: medial, Cr: cranial.
