# Supplementary material for: How do forelimb long bones adapt in rhinoceroses? An in‐depth examination of their microanatomy
Source: J Anat. 2026 Jun 1:10.1111/joa.70180. Online ahead of print. doi: 10.1111/joa.70180 (PMC13398847; doi:10.1111/joa.70180)
Supplement: Supplementary file 8 — Supplementary Data S8. 2D histograms showing the correlation between bone volume fraction and anisotropy. [file JOA-9999-0-s005.docx]

Supplementary data 8. 2D histograms showing the correlation between bone volume fraction and anisotropy.


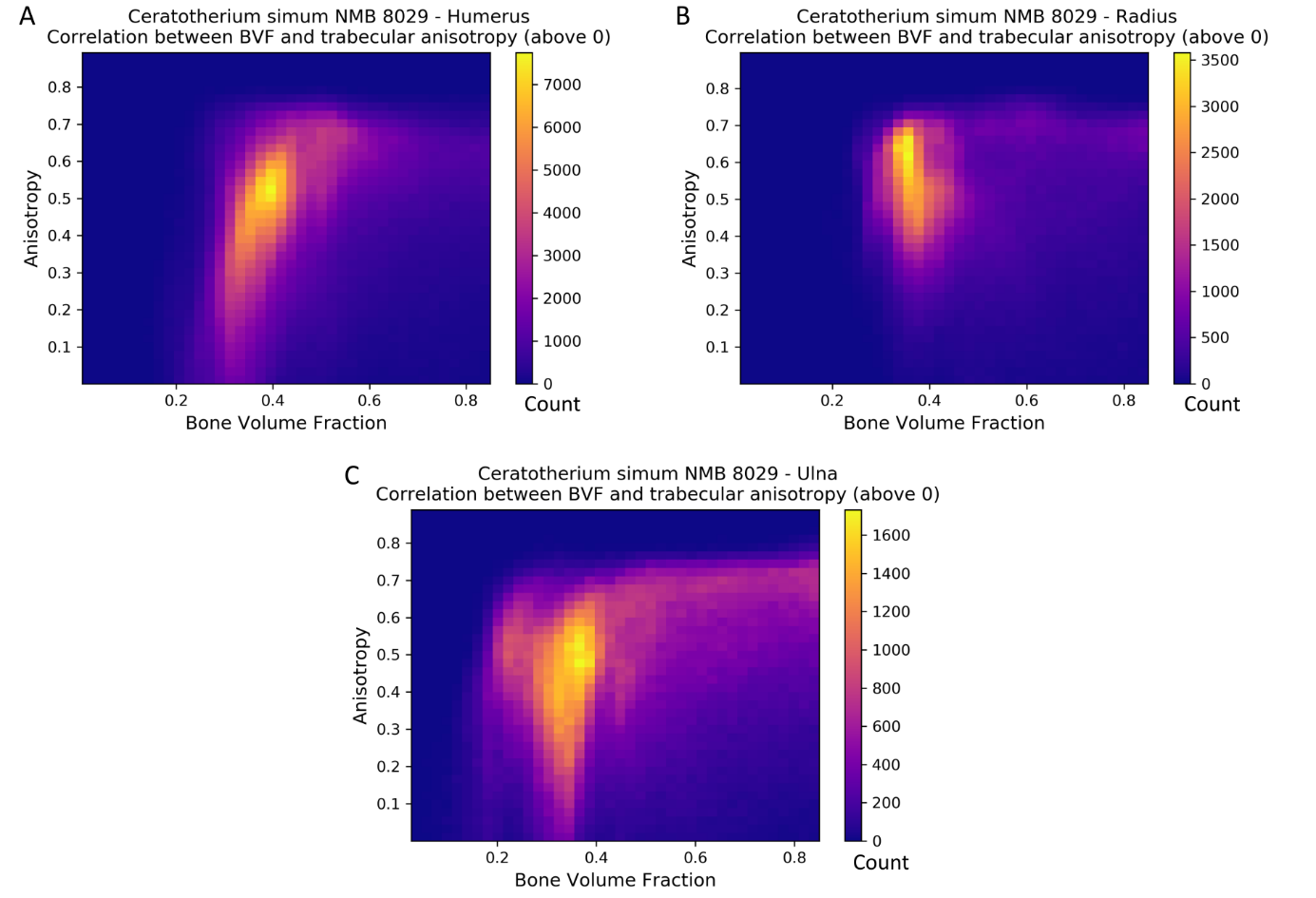


Fig. S8.1. 2D histogram showing the correlation between bone volume fraction and trabecular anisotropy in our studied *C. simum*, for humerus (A), radius (B), and ulna (C). Only ROIs with an anisotropy value above 0 are shown, in order to avoid excessive saturation of the values.


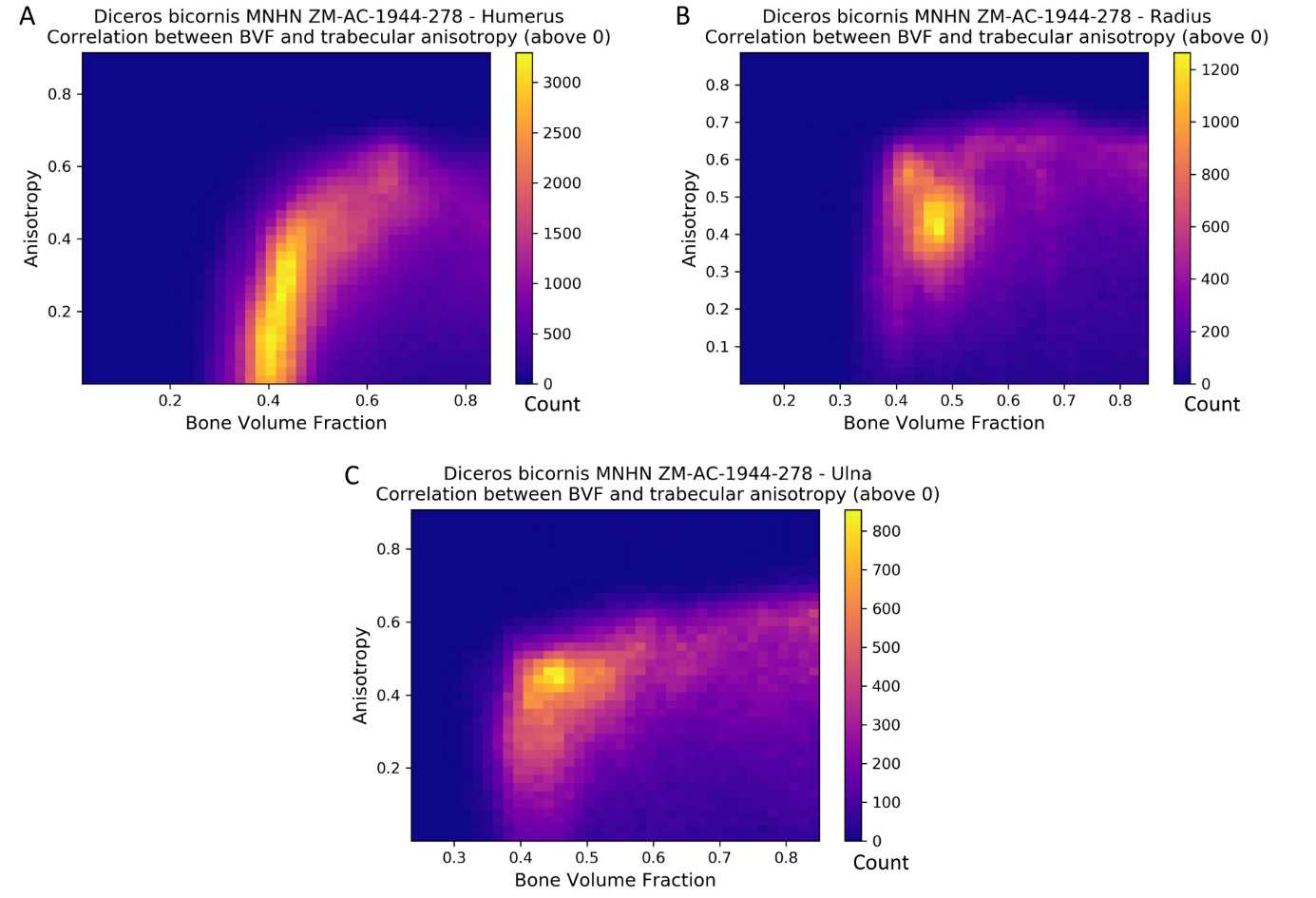


Fig. S8.2. 2D histogram showing the correlation between bone volume fraction and trabecular anisotropy in our studied *D. bicornis*, for humerus (A), radius (B), and ulna (C). Only ROIs with an anisotropy value above 0 are shown, in order to avoid excessive saturation of the values.


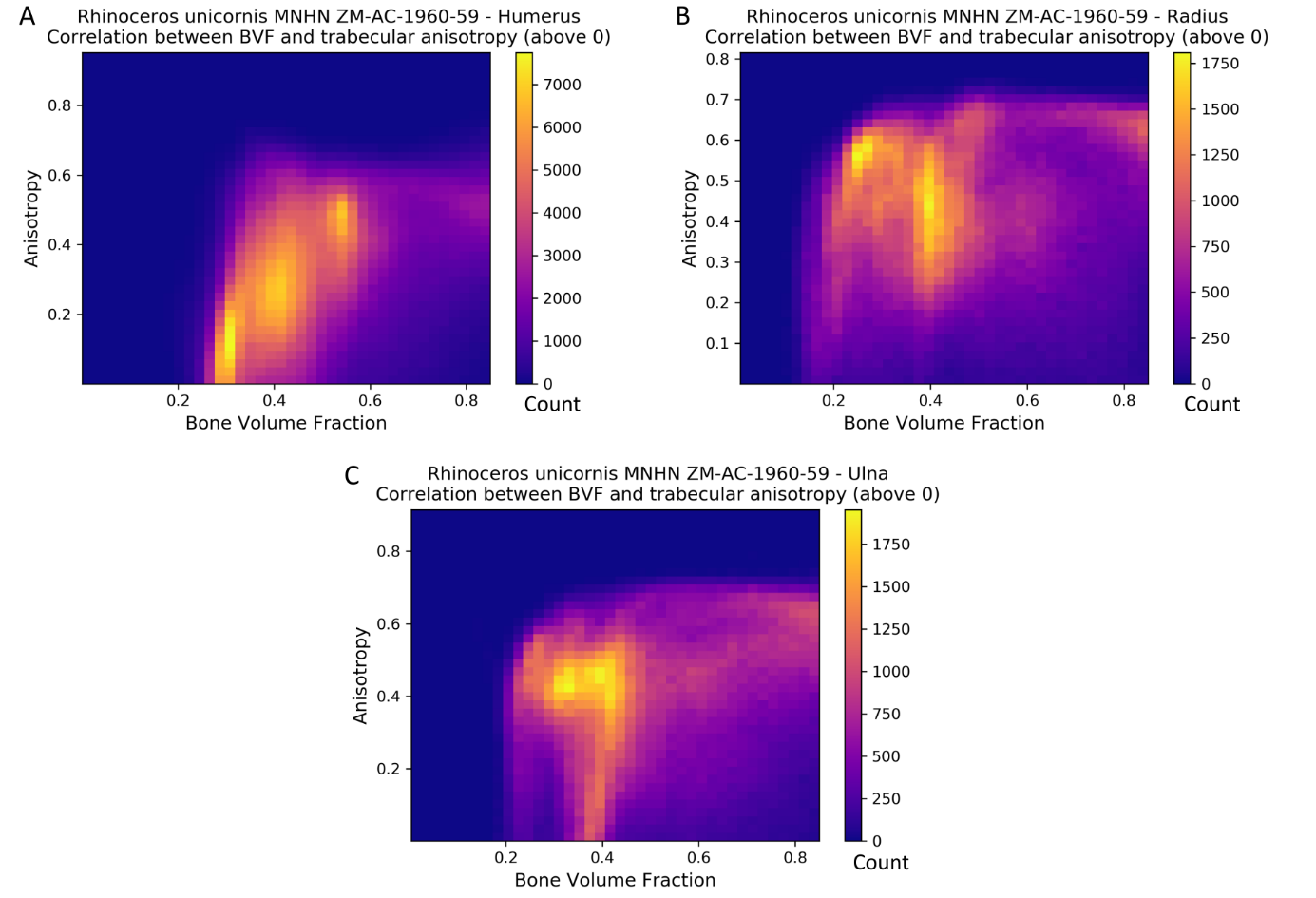


Fig. S8.3. 2D histogram showing the correlation between bone volume fraction and trabecular anisotropy in our studied *R. unicornis*, for humerus (A), radius (B), and ulna (C). Only ROIs with an anisotropy value above 0 are shown, in order to avoid excessive saturation of the values.


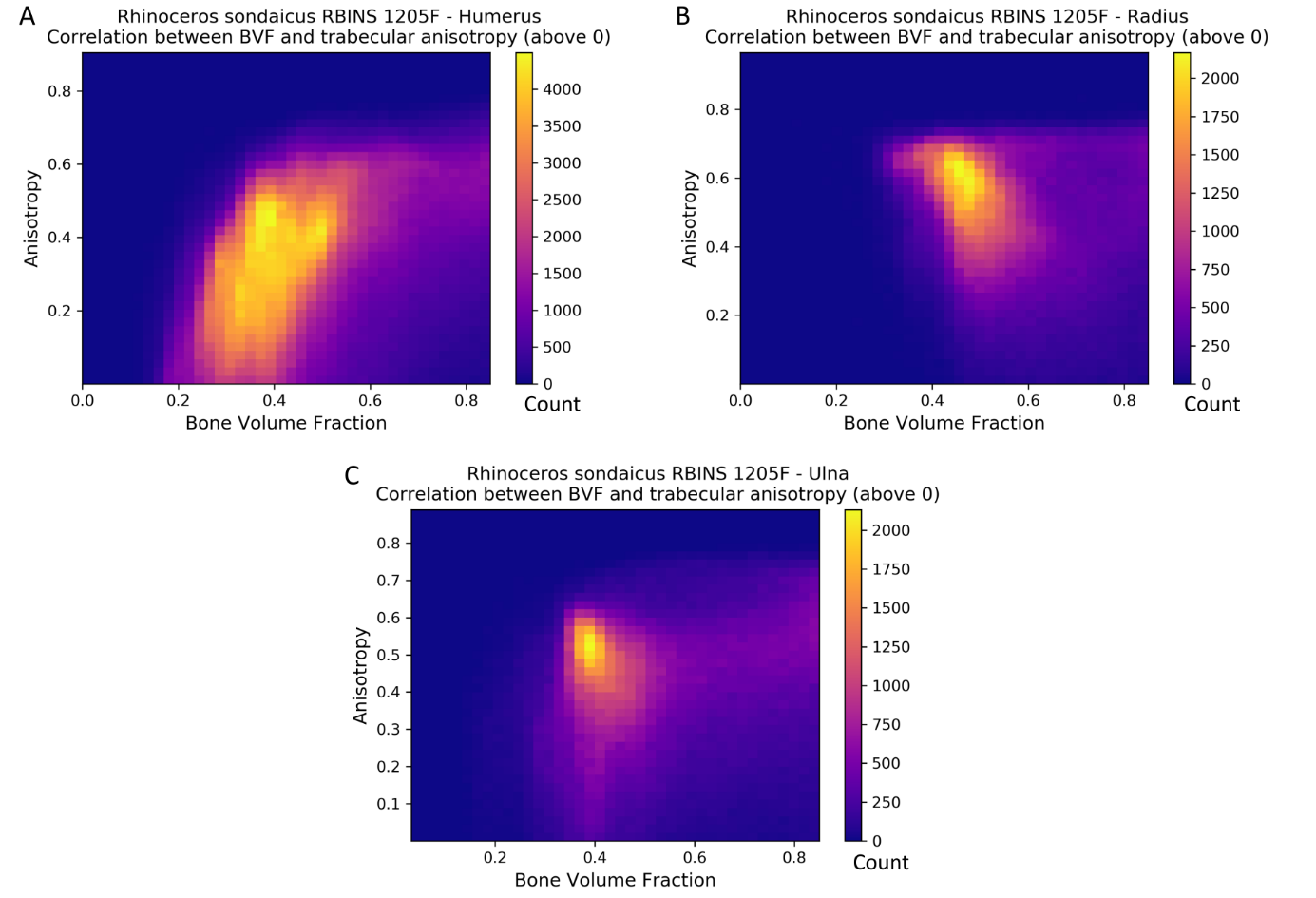


Fig. S8.4. 2D histogram showing the correlation between bone volume fraction and trabecular anisotropy in our studied *R. sondaicus*, for humerus (A), radius (B), and ulna (C). Only ROIs with an anisotropy value above 0 are shown, in order to avoid excessive saturation of the values.


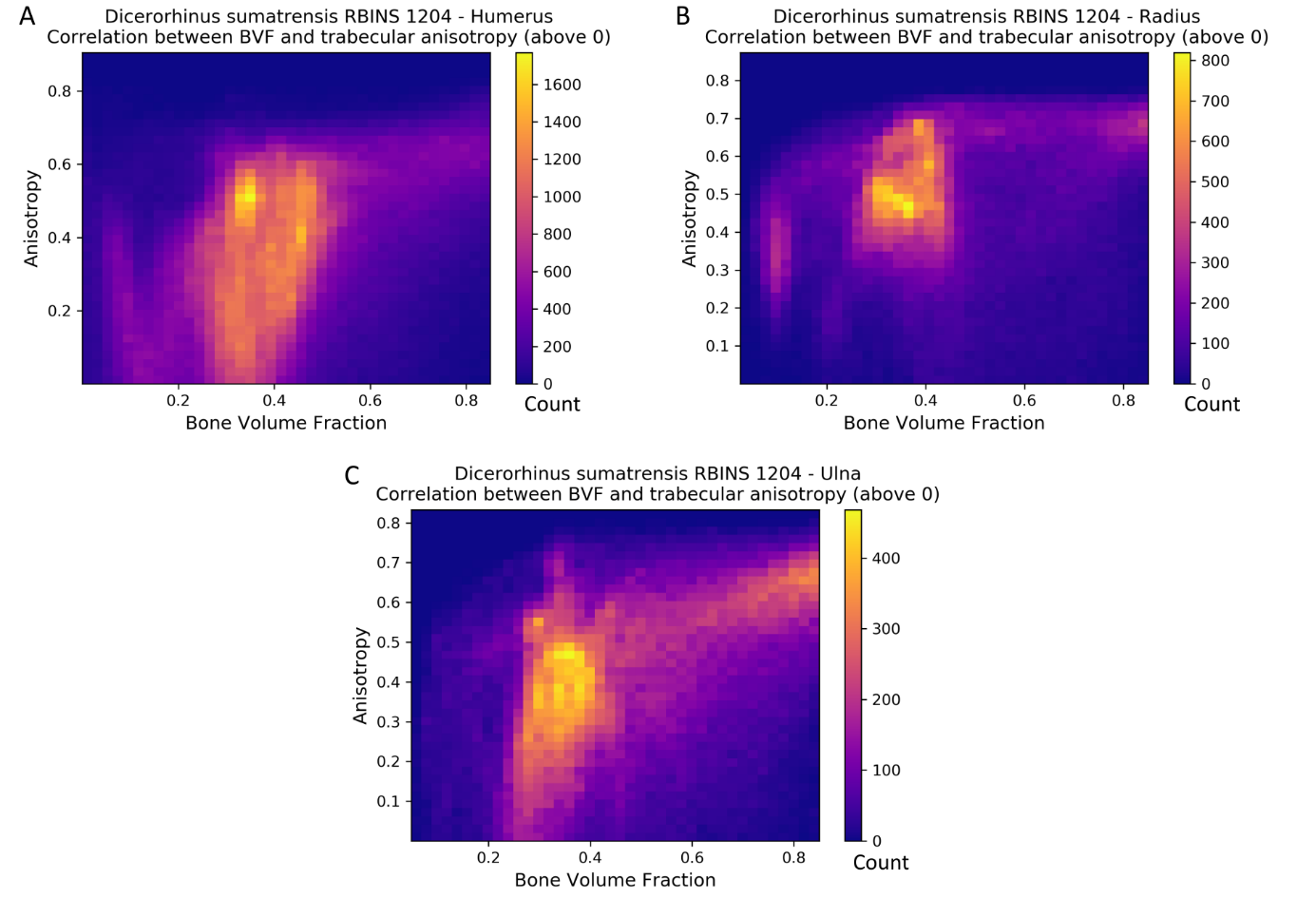


Fig. S8.5. 2D histogram showing the correlation between bone volume fraction and trabecular anisotropy in our studied *D. sumatrensis*, for humerus (A), radius (B), and ulna (C). Only ROIs with an anisotropy value above 0 are shown, in order to avoid excessive saturation of the values.
